# Preparing Effective Narrative Evaluations for the Medical School Performance Evaluation (MSPE)

**DOI:** 10.15766/mep_2374-8265.11277

**Published:** 2022-10-04

**Authors:** April O. Buchanan, Lisa Strano-Paul, Kris Saudek, Carla Lupi, Lee Jones, Mary Jo Wagner, Donna Elliott

**Affiliations:** 1 Professor, Department of Pediatrics, and Associate Dean for Medical Education, University of South Carolina School of Medicine Greenville; Pediatric Hospitalist, Prisma Health; 2 Professor, Department of Internal Medicine, and Assistant Dean for Clinical Education, Renaissance School of Medicine at Stony Brook University; 3 Associate Professor, Department of Pediatrics, and Associate Program Director, Pediatric Residency Program, Medical College of Wisconsin; 4 Professor, Department of Clinical Sciences, and Associate Dean for Assessment and Evaluation, Kaiser Permanente Bernard J. Tyson School of Medicine; 5 Professor, Department of Psychiatry, and Dean for Medical Education, Georgetown University School of Medicine; 6 Professor, Department of Emergency Medicine, Central Michigan University College of Medicine; Chief Academic Officer/DIO, CMU Medical Education Partners; 7 Professor, Department of Pediatrics and Medical Education; Chair, Department of Medical Education; and Vice Dean for Medical Education, Keck School of Medicine of the University of Southern California

**Keywords:** Narrative Assessment, Evaluation, Assessment, Competency-Based Medical Education (Competencies, Milestones, EPAs), Faculty Development

## Abstract

**Introduction:**

In 2016, the AAMC Medical School Performance Evaluation (MSPE) Task Force issued recommendations to standardize the MSPE but did not address the quality of the written narratives in that document. Narrative evaluations are hampered by code words, polite rhetoric, and bias to the detriment of students. To address this, the AAMC's Group on Student Affairs and Group on Educational Affairs convened an expert group to consider the state of narratives in the MSPE and develop resources to improve their quality.

**Methods:**

A series of interactive workshops was developed and presented at an AAMC webinar and national meetings. A presentation outlining challenges and possible approaches to improvement was followed with large-group discussion and/or small-group breakout activity to analyze and improve upon sample clinical comments and create summary clerkship paragraphs. The initial webinar used polling questions and free-text prompts to gather feedback for future workshops. Anonymous survey responses were collected at the end of each subsequent workshop to determine perceived effectiveness and potential utility at participants’ institutions.

**Results:**

Over 680 administrators, faculty, and staff participated in the webinar or in one of four national-level workshops. Respondents agreed that the modules would be useful in faculty development and wanted to replicate their learning at their own institutions for overall better impact on the quality of MSPE narratives.

**Discussion:**

This resource addresses an important gap in the medical education literature. A variety of stakeholders affirmed that these workshops have value in training writers to improve their narrative comments for the MSPE.

## Educational Objectives

After participating in the session, attendees should be able to:

1. Describe the core components of an effective narrative evaluation.
2. Compose a narrative evaluation that provides useful information for students and faculty, with attention to mitigating bias.
3. Construct a summative clerkship narrative evaluation for the Medical School Performance Evaluation (MSPE) that is consistent with AAMC MSPE guidelines.

## Introduction

Narratives for the Medical School Performance Evaluation (MSPE) and letters of recommendation are the only written information submitted by medical schools on behalf of a student that give program directors valuable information about the student's competencies that grades and test scores cannot convey. To provide the substrate of these narratives, faculty and residents in medical schools across the country are required to complete clinical written evaluations of medical students, often with little training. In addition, clerkship directors are tasked with creating summary paragraphs for each clerkship to include in the MSPE and often struggle with deciding which comments to include and how to best represent the student's abilities. The literature reports that student narratives are hampered by polite rhetoric, code words, and bias that negatively impacts students and likely is the result of limited insight and faculty development in writing high-quality narratives.^1–9^ A national survey of program directors reported that across specialties, the majority of respondents valued the narrative comments from the clerkships more than the grade itself.^10^ Interestingly, only a minority of respondents reported trusting the subjective information within the MSPE.^10,11^ With USMLE Step 1 moving from numerical to pass/fail reporting, the medical education community expects a greater value from the written components of the residency application, including the MSPE.^12^ Residency directors have also expressed a desire for greater transparency of comments, specifically, comments that address areas of improvement.^10^ Many program directors advocate improved specificity of comments about clinical performance and the addition of a framework for comments such as competencies,^12^ entrustable professional activities (EPAs),^13^ or RIME (reporter/interpreter/manager/educator).^14,15^

To address the above concerns and enhance the transmission of useful narrative information in the MSPE, the AAMC's Group on Student Affairs (GSA) and Group on Educational Affairs (GEA) initiated a Constituent Collaborative Project to build upon the 2016 guidelines from the MSPE Task Force.^16^ GEA and GSA leadership and staff further refined the goals of the project to include providing programs with more accurate and consistent information about medical students, moving the focus from purely quantitative to qualitative measures of student performance, explicating students’ competencies and professional characteristics, and minimizing bias in the residency application process.

Through a solicitation and selection process, an expert group was assembled to consider the current state of narrative feedback in the MSPE and develop resources to improve the quality of narrative evaluations. Selections were made based on expertise, region, stakeholder status, and level of interest. The Writing Effective Narrative Feedback for the MSPE Working Group convened in 2019. The group began its work with a literature review and search for extant materials. Searching *MedEdPORTAL* using the terms *medical student performance evaluation, MSPE, narrative comments,* and *clerkship comments* returned no citations of existing faculty development resources. A literature review of PubMed was also low yield in terms of faculty development resources but high yield in terms of publications illuminating problems with narratives. Narratives in their current state are lacking in specificity, transparency, and equity, confirming the gaps identified by the AAMC and a need for improvement.^1–9^

Based on these deficits identified in the literature, the working group created faculty development materials utilizing the conceptual framework of deliberate practice in order to develop skill expertise in drafting effective narrative evaluations. In addition to education for faculty and residents who complete student written evaluations, the materials also provide clerkship directors deliberate practice using a structured approach in synthesizing the written clinical evaluations into a final clerkship summative narrative for the MSPE.

## Methods

### Development and Curricular Context

Members of the working group were assigned goals for development and dissemination of the materials according to their areas of expertise with the intention of creating activities for deliberate practice that filled gaps for specificity, transparency, and equity. The group met monthly to review progress, fostering a collaborative environment where all members contributed at an elevated level.

Workshop participants needed to have working familiarity with the ACGME competencies, EPAs, and PRIME (professionalism/reporter/interpreter/manager/educator) frameworks. Facilitators had to have experience with documenting direct observation of clinical performance and with composing summary paragraphs for the MSPE.

### Description of Workshop/Webinar

Using its expertise in MSPE development and educational best practices, informed by the literature review, the work group structured this resource to provide participants with opportunities to utilize common frameworks (i.e., ACGME competencies, EPAs, RIME, PRIME) to illustrate how individual students uniquely demonstrate their clinical learning and to assemble those narratives into a coherent clerkship paragraph. Within these frameworks, the workshop also invited participants to deal with the challenges of presenting gaps in learning and behavior, framing responsiveness to feedback as a strength rather than a reference to deficiency, and identifying bias in written comments. Polling questions were designed to foster participant awareness of others’ roles and institutional practice. Major components of the workshops were as follows:

1. Background information on the project, featuring perspectives from both undergraduate and graduate medical education, challenges in writing narratives, and elements of good narratives (including optional polling questions for engagement): PowerPoint slide deck, Appendix A; workshop facilitator guide, Appendix B.
2. Common frameworks for structuring effective narrative evaluations: Appendix A.
3. Sample clinical narratives for practice in identification of ACGME competencies, EPAs, PRIME elements, and knowledge/skills/attitudes: Appendix A; handout, Appendix C.
4. Sample clinical narratives with an opportunity for participants to suggest improvements, with exemplars to reinforce the section: Appendix A; handout, Appendix D; facilitator guide, Appendix E.
5. Sample narrative comments for participants use in creation of a clerkship summary paragraph, with preamble instructions: handout, Appendix F; facilitator guide, Appendix G.
6. Session feedback/workshop evaluation: Appendix H.

### Implementation

The workshop was presented at multiple national meetings beginning in March 2020. Due to the impact of the COVID-19 pandemic, presentation and dissemination were delayed until October 2020, when we offered an AAMC national webinar for the medical education community. In fall 2020 and spring 2021, sessions were held using a virtual platform at national meetings for medical educators, including the AAMC GSA National Meeting, the Council on Medical Student Education in Pediatrics (COMSEP) National Meeting, the Alliance for Academic Internal Medicine (AAIM) Annual Meeting, and the AAMC GEA Combined Regional Meetings.

The 1-hour webinar largely used a unidirectional presentation approach, with questions posed in the chat section. The webinar covered all of elements 1–3 above.

For the workshops, all of which occurred remotely and included clerkship directors or others writing summary paragraphs, all material in elements 1–6 above was delivered, with activity formats adapted for audience size. Audiences ranged from 18 to 165, with session lengths of 60–90 minutes.

Activity 1 (Appendix A) was used in the large-group interactive mode to ensure that all participants had exposure to the spectrum of examples offered for the competency frameworks. Appendix C allowed for this activity to be done as a breakout group.

Activities 2 and 3 were done in small-group breakouts, linking the handouts in the chat. Appendix B provided a facilitator guide for the slides and specific instructions and options for breakout activities.

For activity 2, faculty and resident rewrite practice, facilitators could choose to review specific examples and solicit feedback from the audience or use a handout (Appendix D) and allow participants to improve individual narratives within small groups. Since these sessions were done virtually with large audiences, four of the presentations opted to present narratives with suggested improvements (included in slides in Appendix A), and one of the longer sessions with smaller numbers of participants opted for breakout groups, linking Appendix E in the chat and then sharing on the screen in the breakouts. The latter allowed the facilitators to better determine overall participation and achievement of Educational Objective 2.

Activity 3, the creation of a clerkship summary paragraph, was the most complex of the three activities. Groups were assigned to complete one of the two cases on the handout and then share it with the larger group. All five of the workshops/presentations included clerkship directors or others combining narrative comments into a summary clerkship paragraph; therefore, we included all the sections described and also the slides on writing summative clerkship paragraphs. For this portion of the workshop, a handout (Appendix F) was linked in the chat and displayed for breakout groups (alternatively, it could be given on paper for in-person sessions). Sample narratives were provided, and participants were asked to create a clerkship summary paragraph for inclusion in the MSPE. Participants reflected on the core components of an effective narrative evaluation covered earlier in the session (Educational Objective 1) and actively labeled these components. The facilitator guide for this portion of the session (Appendix G) was shared with the audience upon completion of the activity, allowing them to reflect on achievement of Educational Objective 3.

### Evaluation Strategy

The main strategies employed in the initial webinar were polling questions and feedback provided by participants in the chat. Based on this feedback, evaluation questions were developed for the GEA and GSA presentations. These evaluations were collected anonymously at the end of each workshop through an electronic survey form posted in the chat to measure the effectiveness of the activity. Responses were analyzed by the working group to further refine the materials.

As described above, breakout groups allowed facilitators to actively engage participants and ensure attainment of all three Educational Objectives. Evaluation for Kirkpatrick's level 1 (reaction) included evaluating participation, how modules could be helpful at institutions, and which sections were most helpful. In considering Kirkpatrick's level 2 (learning), evaluation included directed observation by facilitators of participants engaging in revising sample narratives and creating final clerkship summary paragraphs using the recommended format.^17^

An additional evaluation form previously developed by a team of pediatric medical educators with peer review was completed at the longer, interactive COMSEP workshop to better understand whether the deliberate practice resulted in individual acquisition of new knowledge and skills and was a worthwhile investment in participants’ professional development. This form could also be used by institutions in evaluating the workshop with their audiences (Appendix H).

## Results

### Participant Characteristics and Experience With the MSPE

The workshops were conducted a total of five times during the fall of 2020 and spring of 2021 in a series of interactive faculty development sessions:

• The invitations for the AAMC webinar in October 2020 went to all members of the GSA and GEA, in addition to general AAMC membership. This audience included senior associate deans for medical education, associate and assistant deans for curriculum and academic affairs, assistant and associate deans for student affairs, career advisors, clinician educators, and staff. A total of 403 participants attended this webinar.
• The AAMC GSA National Meeting was held in April 2021. At this session, there were 165 participants, including student affairs faculty, administration, and staff.
• The COMSEP National Meeting in April 2021 had 40 participants, including pediatric clerkship directors, teaching faculty, and administrators.
• The AAIM Annual Meeting in April 2021 included 18 participants who were internal medicine clerkship directors.
• The AAMC GEA Combined Regional Meetings in April 2021 had a total of 60 participants, including administrators, faculty, and staff from UME, GME, and CME.

The webinar, GSA, and GEA presentations included polling questions whose results are shown in Table 1. The total number of participants was recorded for the GSA, but the number of polling question respondents was not, so that number is missing from the table.

**Table 1.**
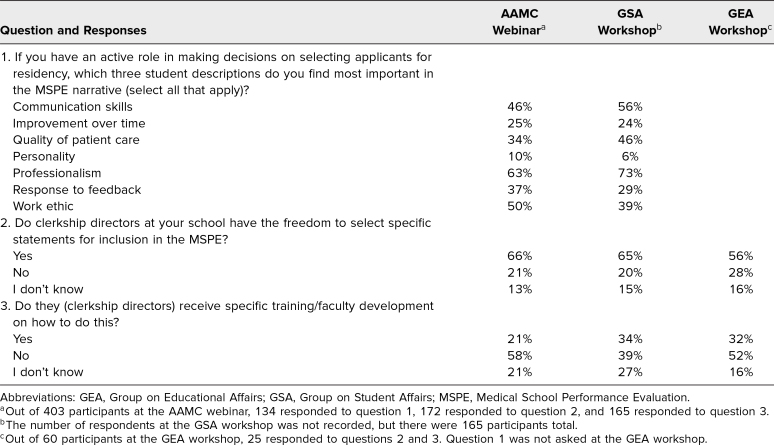
Participant Responses to Polling Questions From the AAMC Webinar and GEA and GSA Workshops

**Figure 1.**
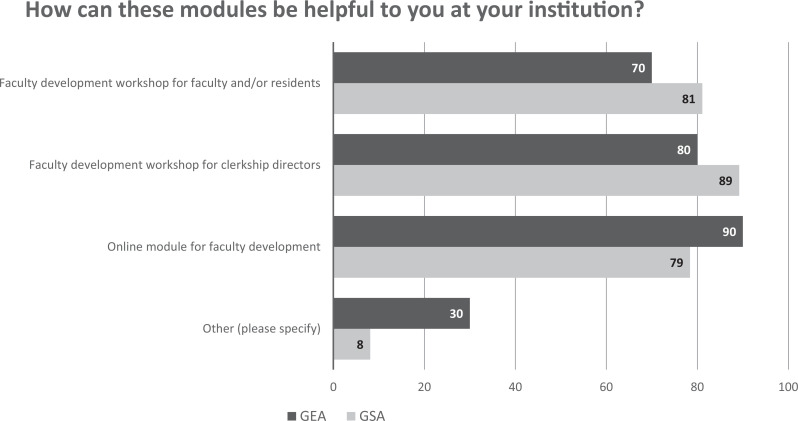
Percentage of GEA (*n* = 10/60) and GSA (*n* =37/165) respondents who indicated how the modules could be helpful for their institution. Abbreviations: GEA, Group on Educational Affairs; GSA, Group on Student Affairs.

**Figure 2.**
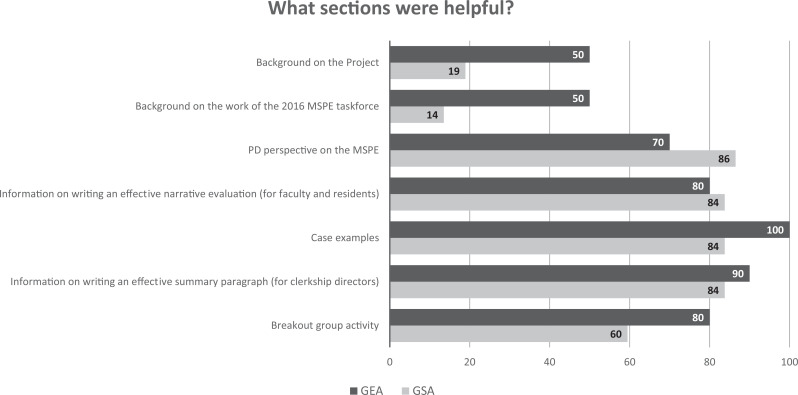
Percentage of GEA (*n* = 10/60) and GSA (*n* = 37/165) respondents who selected which sections of the workshop were helpful. Respondents could select all that applied. Abbreviations: GEA, Group on Educational Affairs; GSA, Group on Student Affairs; MSPE, Medical School Performance Evaluation; PD, program director.

Participants at both the GSA and GEA meetings indicated that this workshop/module would be useful in faculty development for faculty and/or residents, in faculty development for clerkship directors, and as an online module (Figure 1). Other suggested utilizations of the workshop included development for student affairs administrators, clerkship coordinators, and other medical school staff. Participants found the program director perspective, guidance on writing a clinical narrative and a clerkship summary, and case examples to be highly useful (Figure 2). In addition, feedback from the AAIM presentation rated the effectiveness of the workshop (18/18 participants) as 4.2 on a 5-point Likert scale (1 = *very dissatisfied,* 5 = *very satisfied*). At the COMSEP meeting, the overall rating of the workshop's effectiveness was 4.6 on a 5-point Likert scale (1 = *strongly disagree,* 5 = *strongly agree*; Table 2).

**Table 2.**
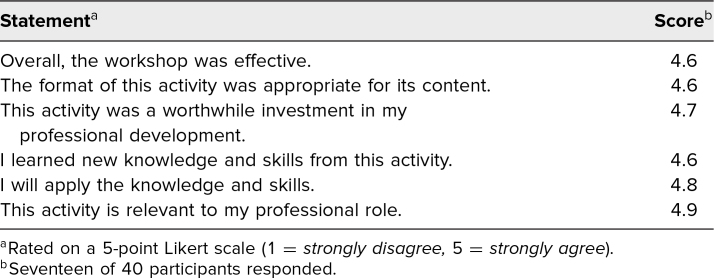
Evaluation Results From the Council on Medical Student Education in Pediatrics Workshop

Highlights of representative comments included the following:

• “It is always helpful to keep hearing we need to make note of student weaknesses and their improvement despite student protests to remove such comments.”
• “Practical information that anyone who helps summarize what goes into the MSPE should be exposed to. This clearly is not being done routinely by individual medical schools (at least based on what everyone in the workshop mentioned) so I found this workshop to be very useful.”
• “The case-based examples and breakout groups were helpful—having the opportunity to re-structure examples for both good and sub-par students in a group setting provided a great opportunity for team-based learning.”

Kirkpatrick's level 1 (reaction) was measured through participation and evaluation, which demonstrated that participants found the workshop engaging, favorable, and relevant. In considering Kirkpatrick's level 2 (learning), each facilitator was able to review the groups’ participation and work on the screen during the breakouts while participants revised the sample narratives and final clerkship summary paragraphs.^17^ There was clear demonstration of attained skills as participants were able to reword narratives and write paragraphs using the information learned in the session. As far as behavior and impact, the overall goal of this project and dissemination was that participants take what they learned from the sessions and replicate it at their own institutions for overall better impact on the quality of narratives in the MSPE.

## Discussion

The Writing Effective Narrative Feedback for the MSPE Working Group has addressed an important gap in the medical education community as identified by the AAMC and as affirmed by the attendance at different iterations of the workshop. This work comes at a critical point as USMLE is moving to pass-fail scoring in 2022, further limiting quantitative data and making other data in the MSPE (and the residency application overall) of greater importance. Results of the surveys from participants attending the sessions were uniformly positive and underscored the need for resources to foster the skills required to produce clinical narratives and summary clerkship paragraphs. In addition, respondents’ comments supported the overall utility of individuals providing training to faculty and residents, as well as the helpfulness of an online module/workshop that could be used by individuals across various locations.

The work group's development of all parts of the project and full engagement contributed to the project's overall success. The effort was supported by AAMC staff members and allowed for a synthesis of expertise from a nationally representative group of expert stakeholders. An extensive literature review informed the creation of needed resources to support faculty and resident development in this area.

The implementation of this project was impacted by the COVID-19 pandemic. Initial sessions were submitted to and accepted at national and regional conferences that were later canceled or converted to virtual platforms. Even though the literature has described less robust engagement in online workshops,^18^ our group was able to pivot and adapt the materials to virtual sessions with strong faculty participation. While the initial webinar provided easy dissemination of information, it was clear that participants wanted more interactive activities and practice. Additional iterations utilized modules for small-group interaction, creating a more structured opportunity for participants to achieve Educational Objectives 2 and 3. Additional time was also spent creating facilitator guides with examples that not only represented overall positive comments but also addressed a systematic approach for negative comments and offered an opportunity to describe student response to feedback, a quality valued by residency program directors. One option for use of this workshop at individual institutions would be for participants to bring some of their own (redacted) narratives and rewrite them as part of activity 2; the same scenario could apply to clerkship directors bringing a series of clinical narratives to compose clerkship summary paragraphs, with feedback from facilitators, for activity 3.

In terms of our evaluation strategy, we wish we had gathered more information from the initial large webinar group, but time was limited. The GSA and GEA interactive workshops did provide useful feedback supporting both the need for the workshop at individual institutions and the effectiveness of deliberate practice, along with clear feedback from facilitators to participants, in achieving goals. After utilizing the evaluation form (Appendix H), which included additional questions about achieving learning objectives and applying knowledge and skills, in the COMSEP interactive workshop, we believe this form would be most beneficial for individuals to use at their own institutions. In addition, it would be interesting to know if, in the future, participants change the way they write their narratives and sustain this change.

Overwhelmingly, participants valued the virtual live presentation and suggested making it even longer. The appeal of adapting our work for asynchronous use, a strategy endorsed by participants in our online synchronous workshops, is increased accessibility to a larger group of participants who have variation in their daily schedules. For those faculty wishing to offer a live, in-person workshop, the materials require no modification.

The work group convened to tackle the most subjective portion of the MSPE and create a module for faculty development in writing high-quality narratives, with attention to mitigating bias. If the MSPE is truly to evolve into the trustworthy document medical educators call for, processes allowing for increased transparency in the UME-to-GME handoff must be coupled with the mindset that learners all have gaps and that student coachability and response to feedback are critical assets rather than deficits. We believe this workshop provides a framework for incorporating these perspectives and supports faculty and clerkship directors in the required skills.

## Appendices


Narrative Evaluations for the MSPE.pptxFacilitator Guide.docxActivity 1.docxActivity 2.docxActivity 2 Facilitator Guide.docxActivity 3.docxActivity 3 Facilitator Guide.docxEvaluation Form.docx
*All appendices are peer reviewed as integral parts of the Original Publication.*
