# Supplementary material for: Preparing Effective Narrative Evaluations for the Medical School Performance Evaluation (MSPE)
Source: MedEdPORTAL. 2022 Oct 4;18:11277. doi: 10.15766/mep_2374-8265.11277 (PMC9529862; doi:10.15766/mep_2374-8265.11277)
Supplement: Supplementary file 1 — Narrative Evaluations for the MSPE.pptxFacilitator Guide.docxActivity 1.docxActivity 2.docxActivity 2 Facilitator Guide.docxActivity 3.docxActivity 3 Facilitator Guide.docxEvaluation Form.docx [file mep_2374-8265.11277-s001.zip › A. Narrative Evaluations for the MSPE.pptx]

## Slide 1
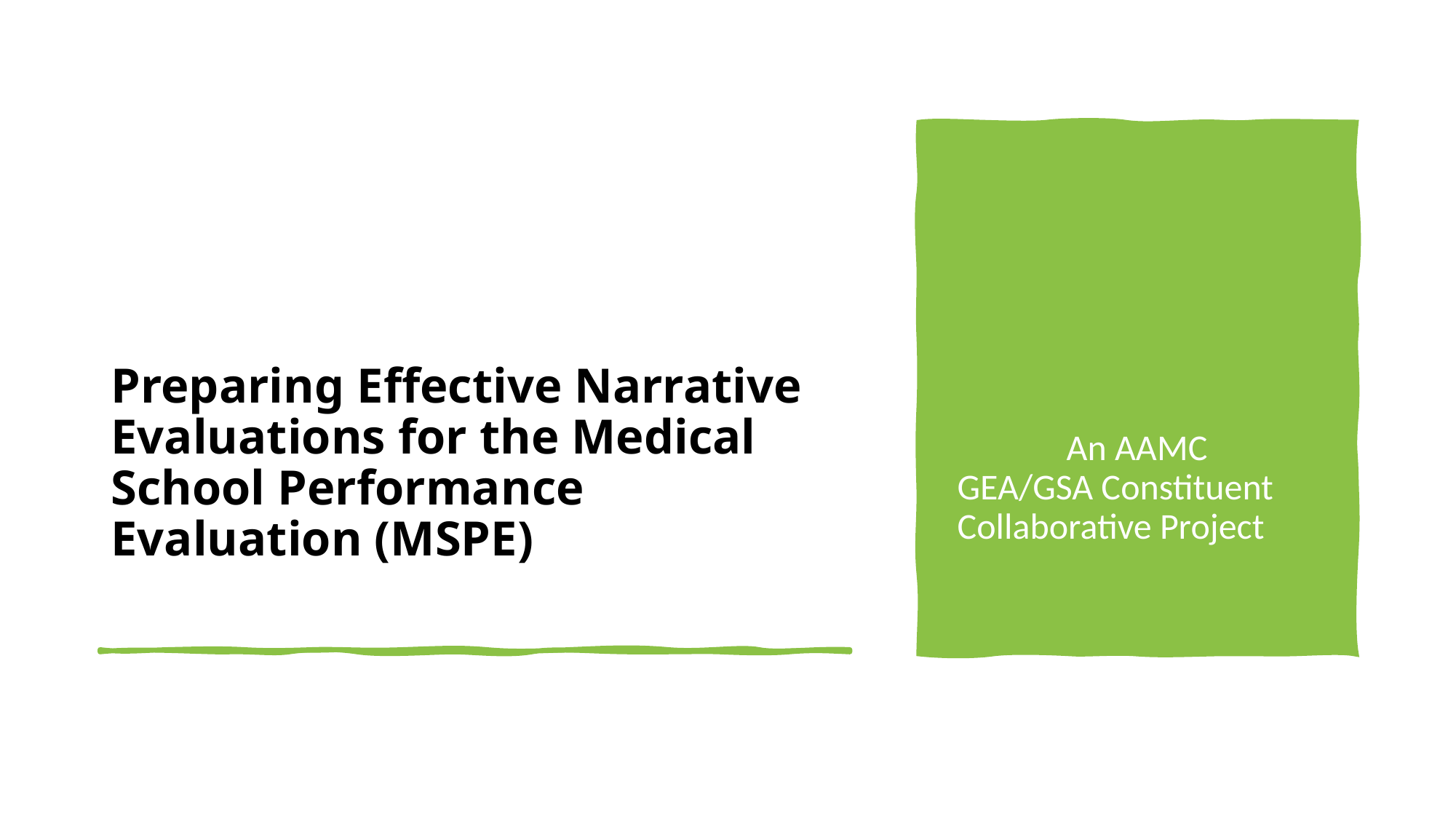

# Preparing Effective Narrative Evaluations for the Medical School Performance Evaluation (MSPE)
	An AAMC GEA/GSA Constituent Collaborative Project

## Slide 2
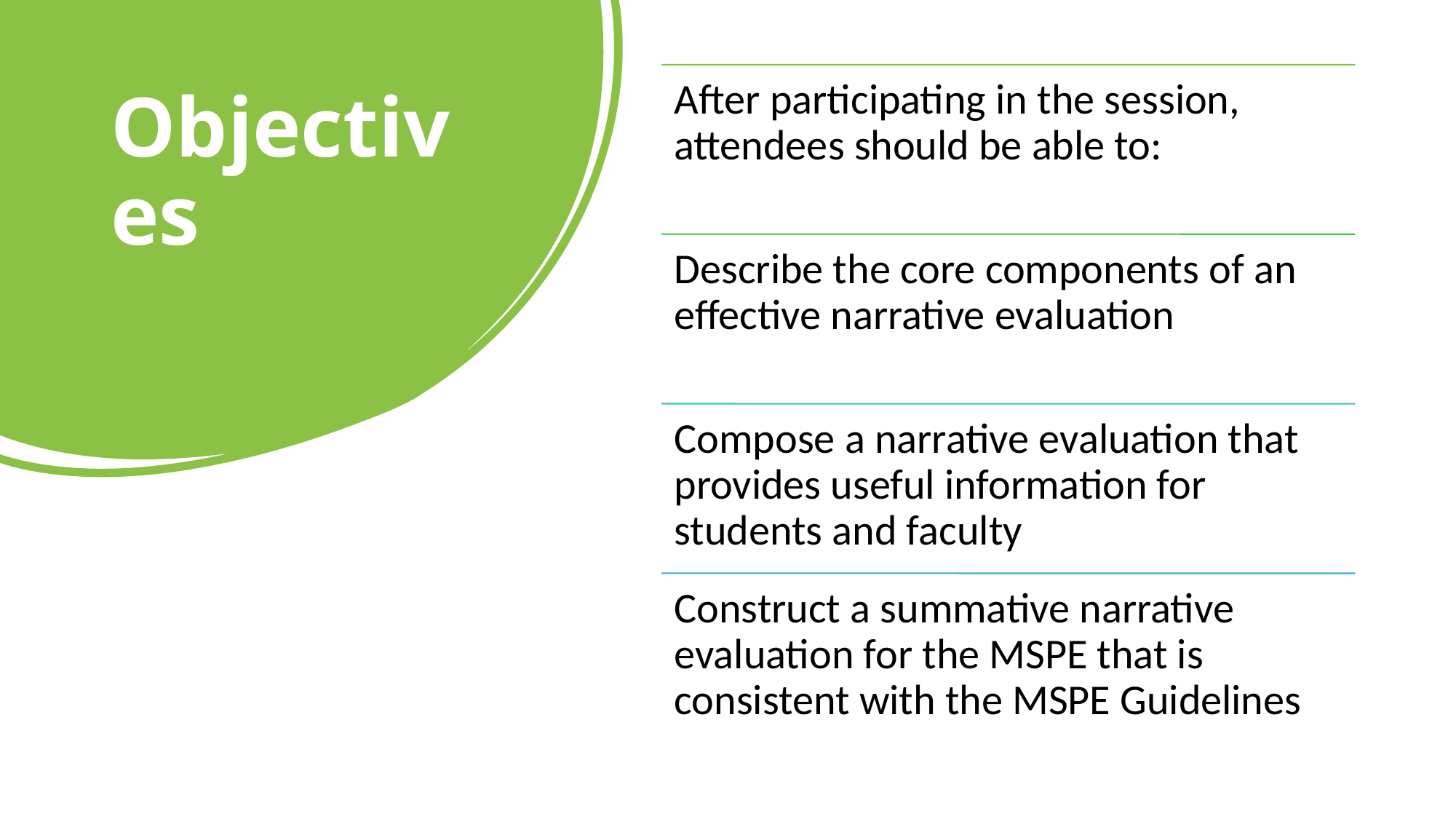

# Objectives

## Slide 3
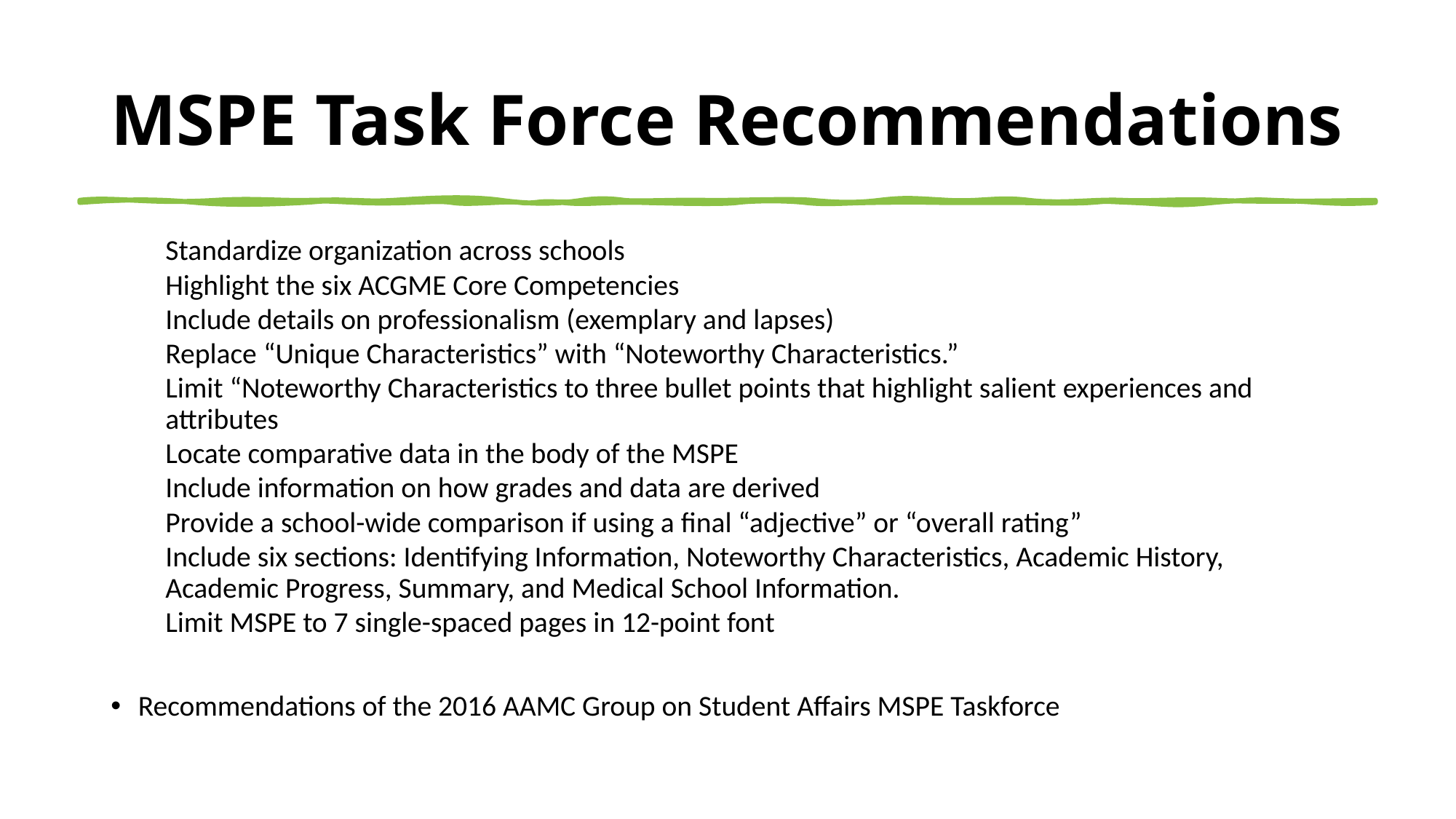

# MSPE Task Force Recommendations
Standardize organization across schools
Highlight the six ACGME Core Competencies
Include details on professionalism (exemplary and lapses)
Replace “Unique Characteristics” with “Noteworthy Characteristics.”
Limit “Noteworthy Characteristics to three bullet points that highlight salient experiences and attributes
Locate comparative data in the body of the MSPE
Include information on how grades and data are derived
Provide a school-wide comparison if using a final “adjective” or “overall rating”
Include six sections: Identifying Information, Noteworthy Characteristics, Academic History, Academic Progress, Summary, and Medical School Information.
Limit MSPE to 7 single-spaced pages in 12-point font
Recommendations of the 2016 AAMC Group on Student Affairs MSPE Taskforce

## Slide 4
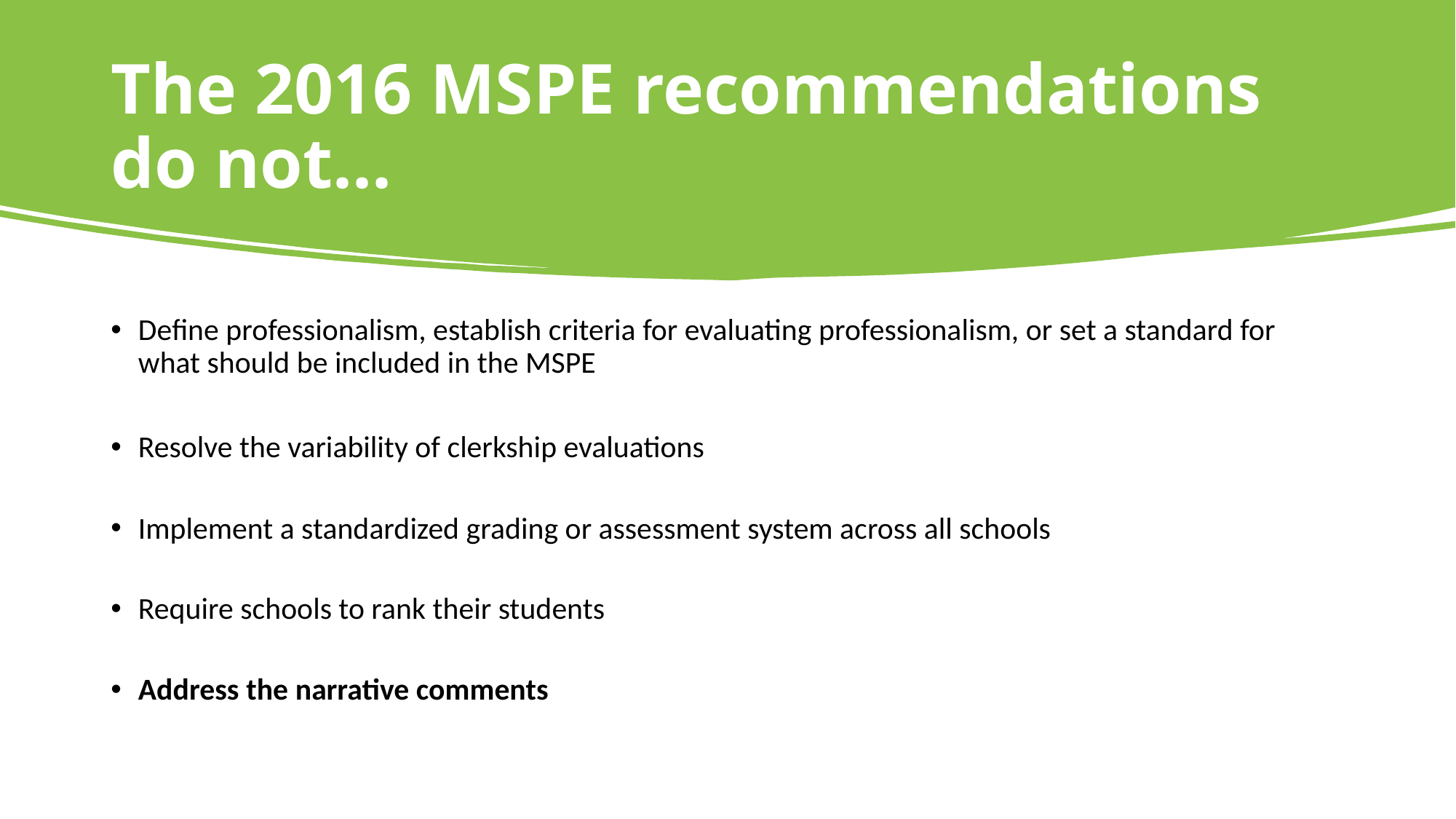

The 2016 MSPE recommendations do not…
Define professionalism, establish criteria for evaluating professionalism, or set a standard for what should be included in the MSPE
Resolve the variability of clerkship evaluations
Implement a standardized grading or assessment system across all schools
Require schools to rank their students
Address the narrative comments

## Slide 5
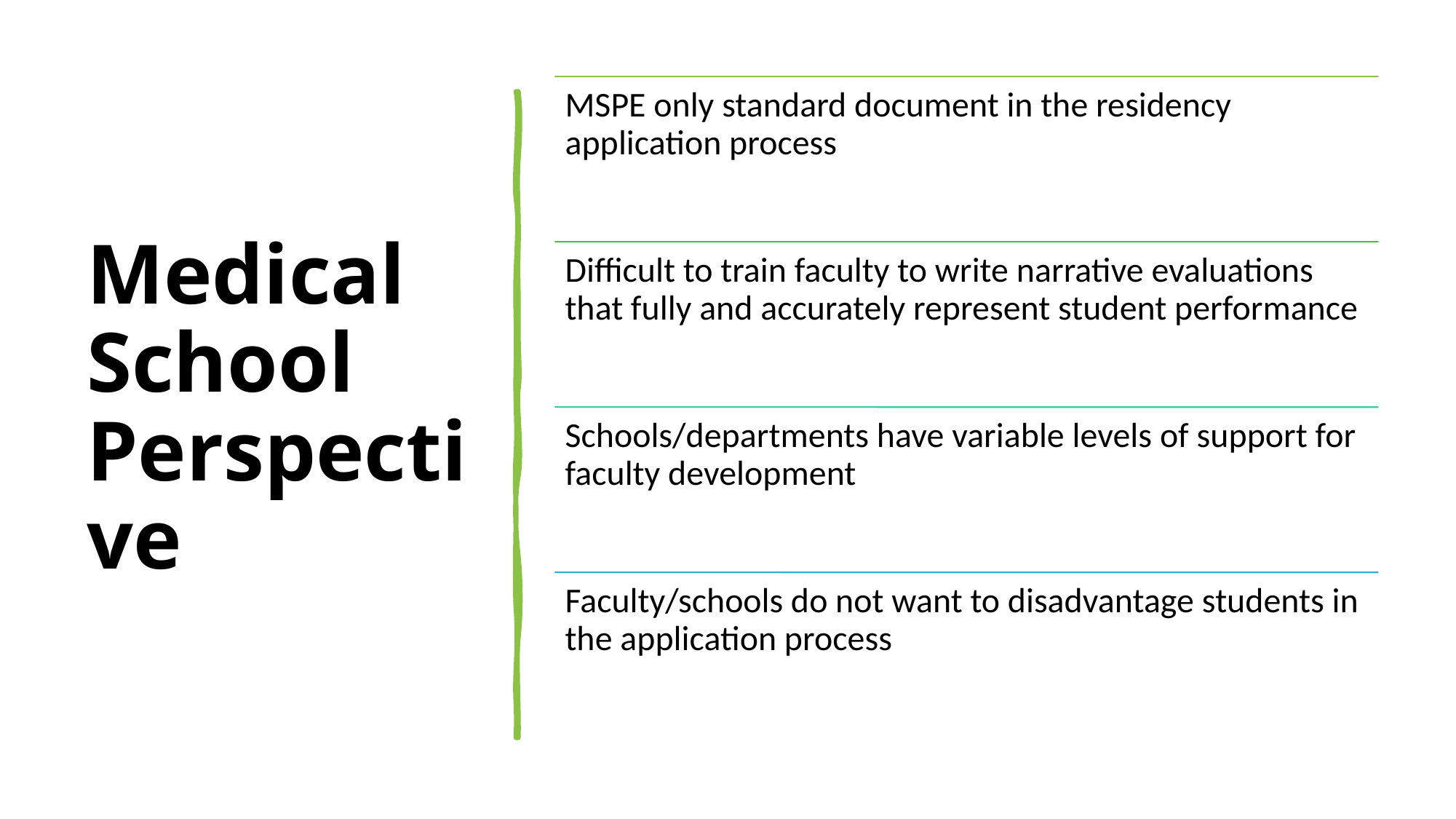

# Medical School Perspective

## Slide 6
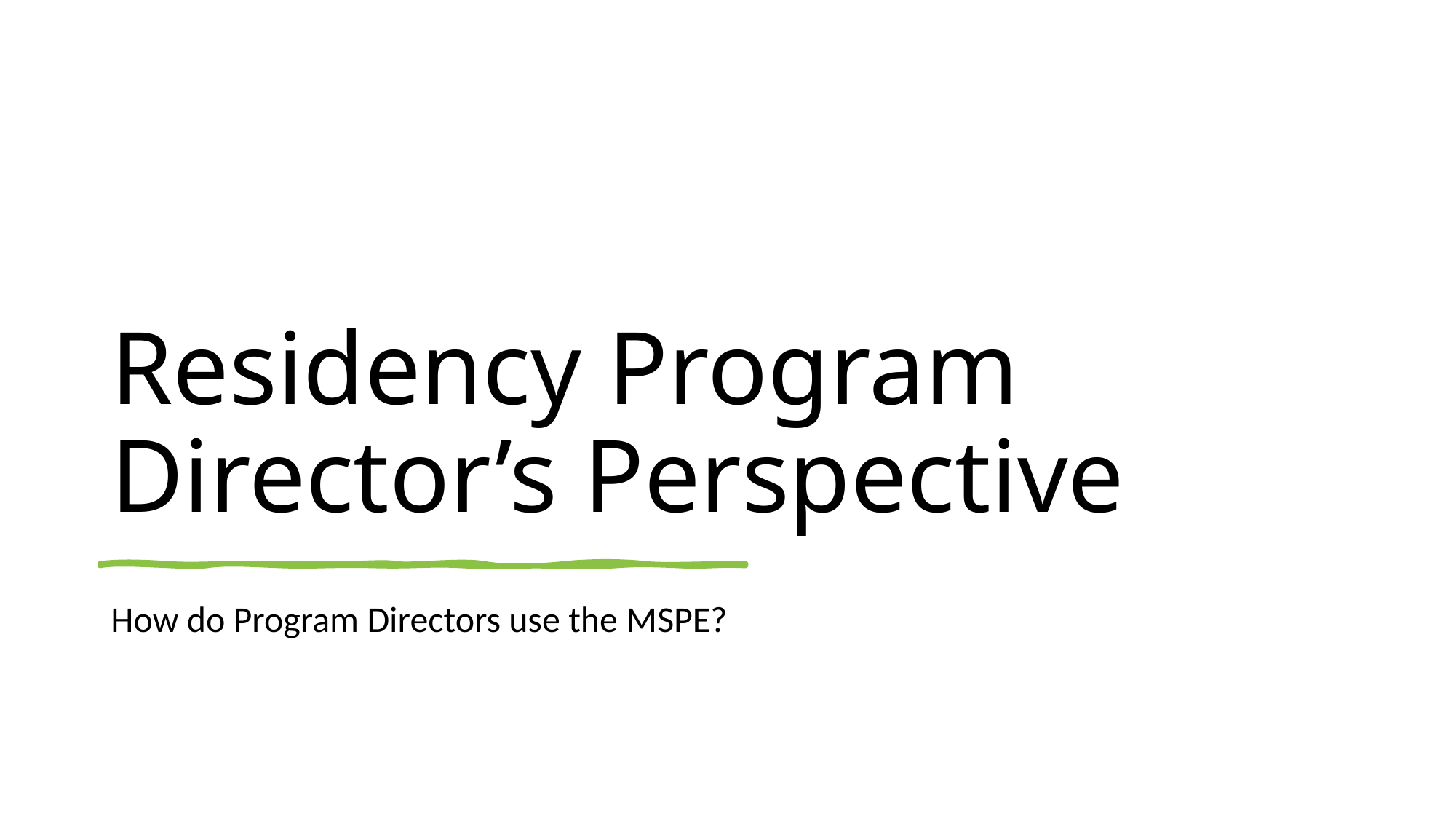

# Residency Program Director’s Perspective
How do Program Directors use the MSPE?

## Slide 7
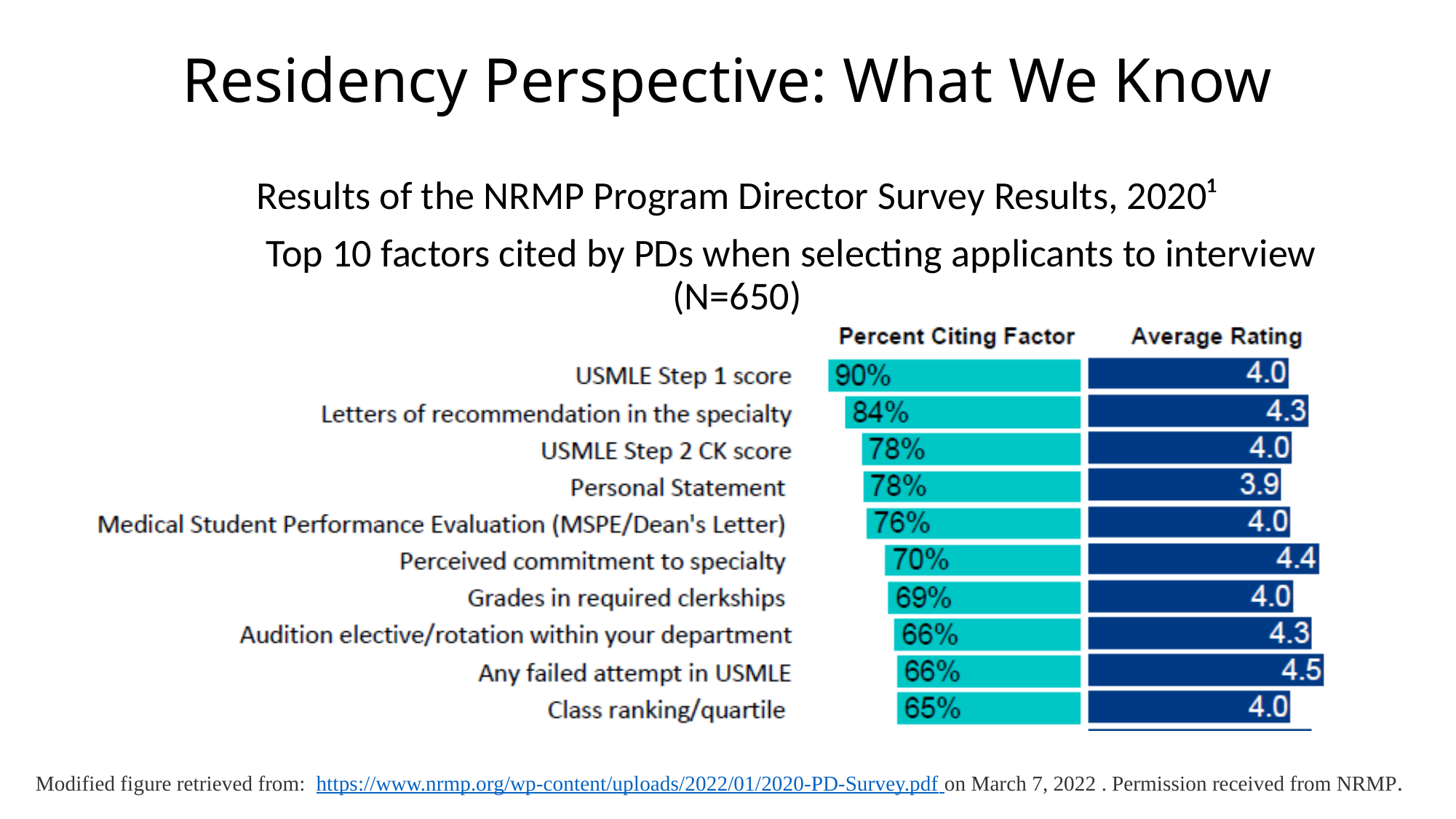

# Residency Perspective: What We Know
Results of the NRMP Program Director Survey Results, 2020¹
	Top 10 factors cited by PDs when selecting applicants to interview (N=650)
Modified figure retrieved from: https://www.nrmp.org/wp-content/uploads/2022/01/2020-PD-Survey.pdf on March 7, 2022 . Permission received from NRMP.

## Slide 8
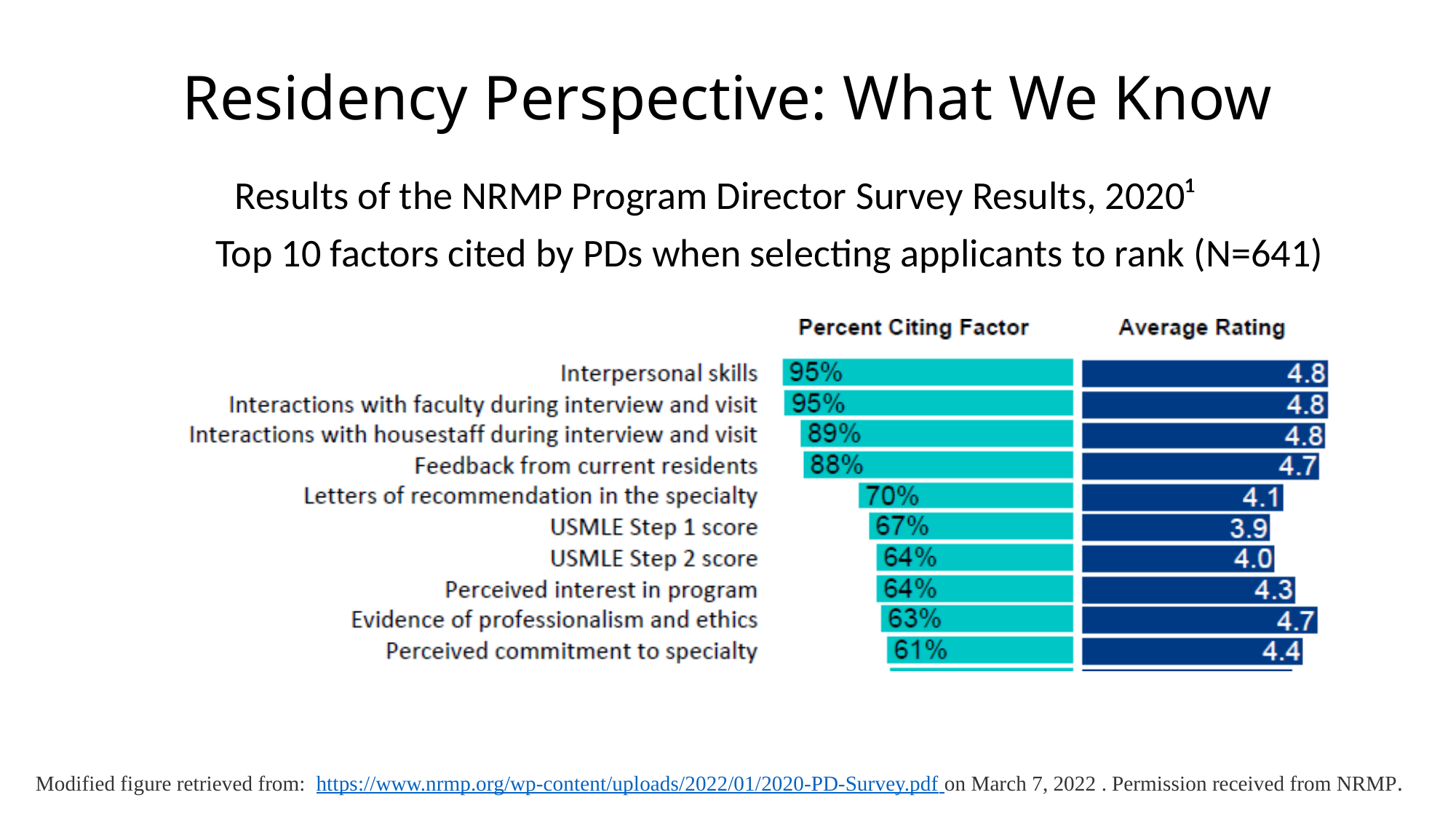

# Residency Perspective: What We Know
Results of the NRMP Program Director Survey Results, 2020¹
	Top 10 factors cited by PDs when selecting applicants to rank (N=641)
Modified figure retrieved from: https://www.nrmp.org/wp-content/uploads/2022/01/2020-PD-Survey.pdf on March 7, 2022 . Permission received from NRMP.

## Slide 9
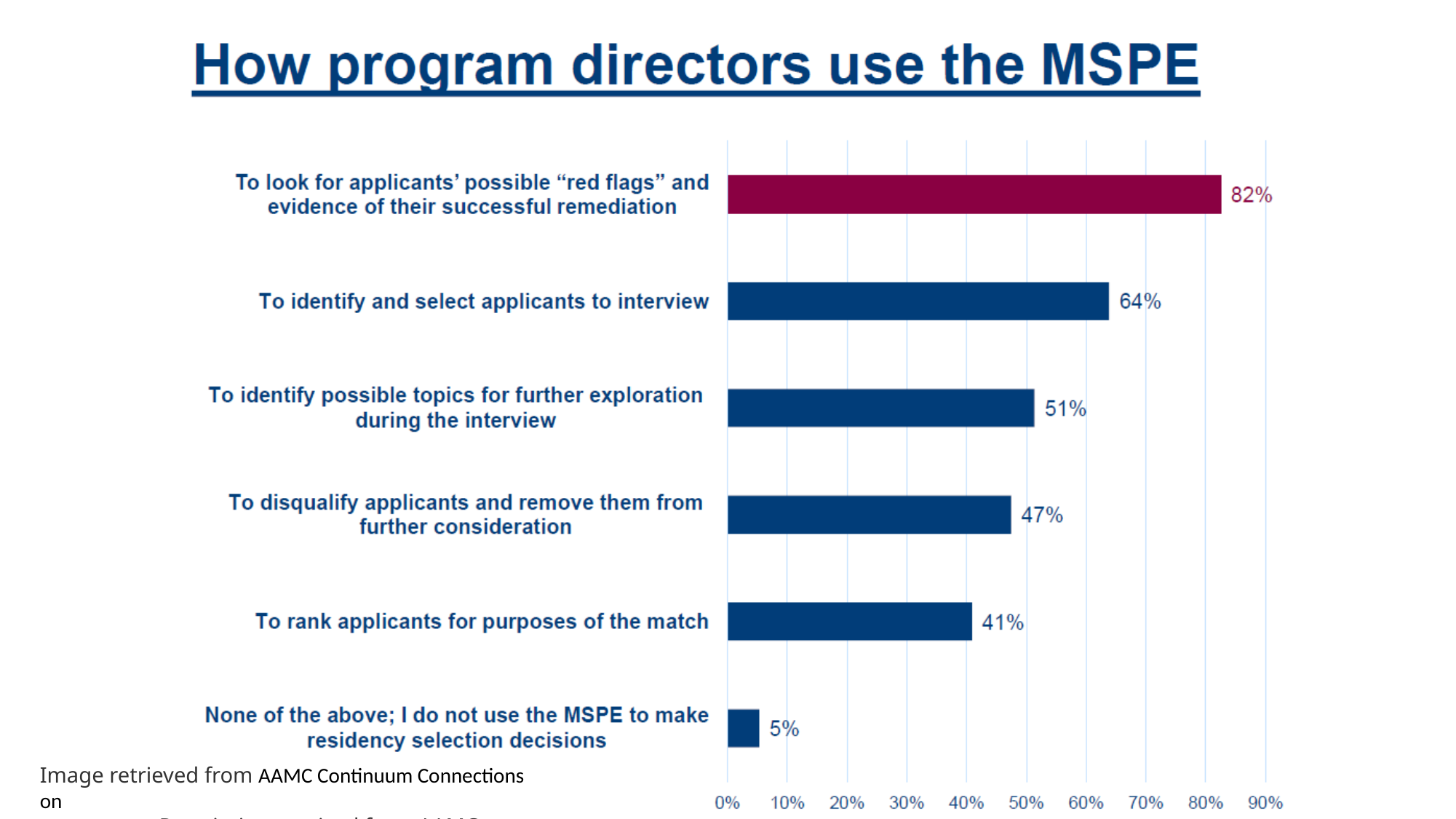

Image retrieved from AAMC Continuum Connections on
Nov 10 2021. Permission received from AAMC.

## Slide 10
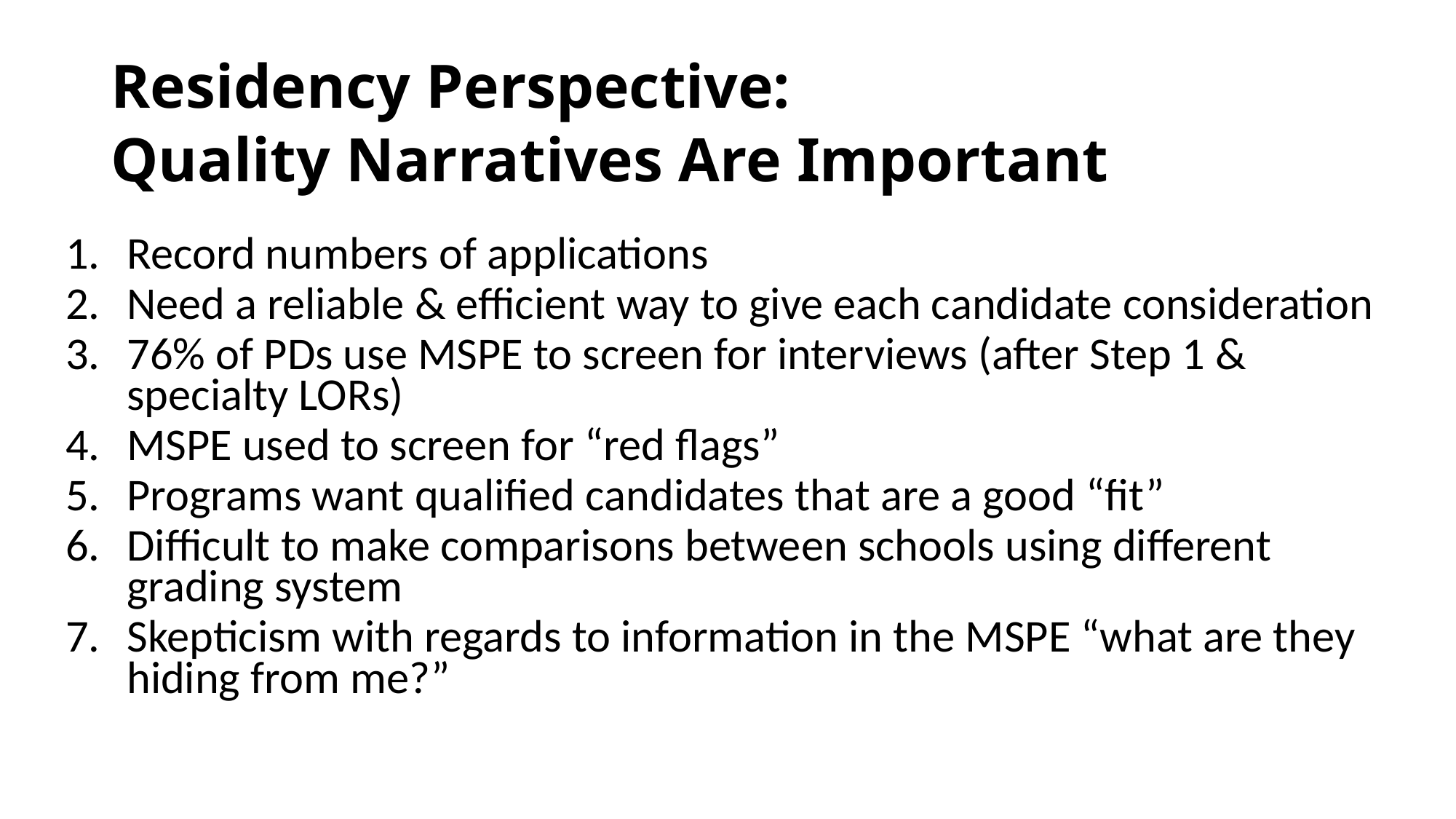

# Residency Perspective: Quality Narratives Are Important
Record numbers of applications
Need a reliable & efficient way to give each candidate consideration
76% of PDs use MSPE to screen for interviews (after Step 1 & specialty LORs)
MSPE used to screen for “red flags”
Programs want qualified candidates that are a good “fit”
Difficult to make comparisons between schools using different grading system
Skepticism with regards to information in the MSPE “what are they hiding from me?”

## Slide 11
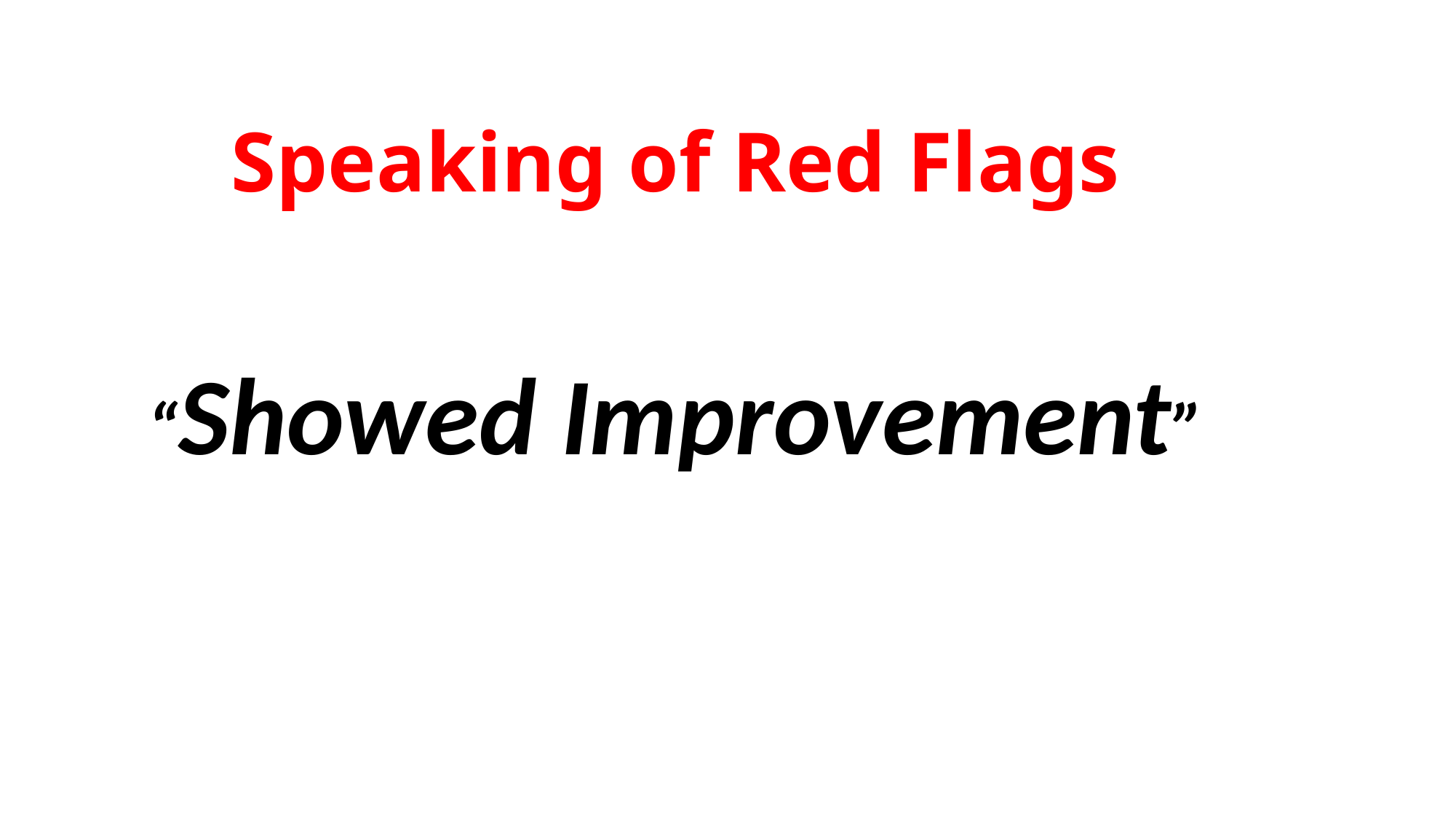

# Speaking of Red Flags
“Showed Improvement”

## Slide 12
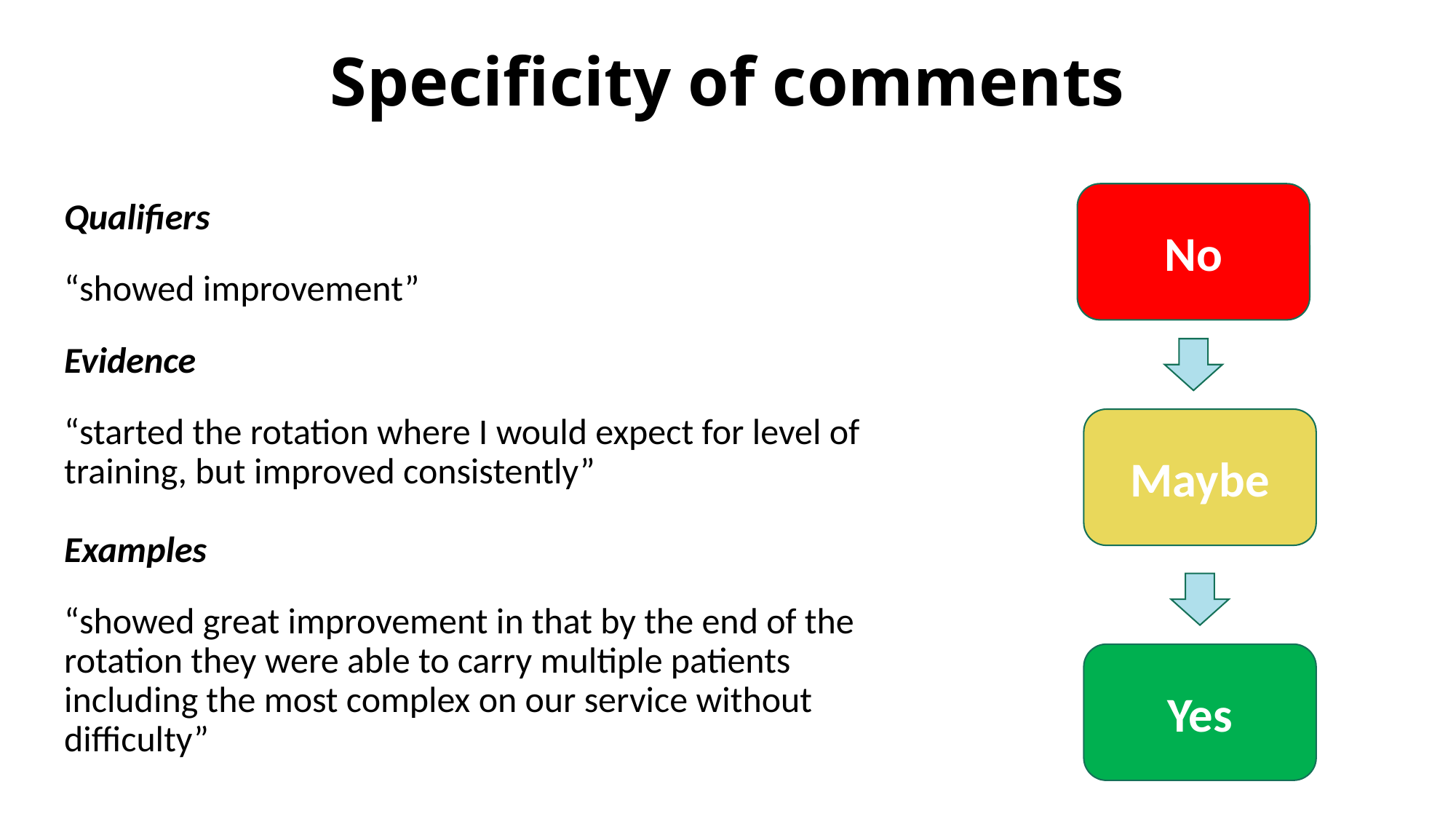

# Specificity of comments
Qualifiers
“showed improvement”
Evidence
“started the rotation where I would expect for level of training, but improved consistently”
Examples
“showed great improvement in that by the end of the rotation they were able to carry multiple patients including the most complex on our service without difficulty”
No
Maybe
Yes

## Slide 13
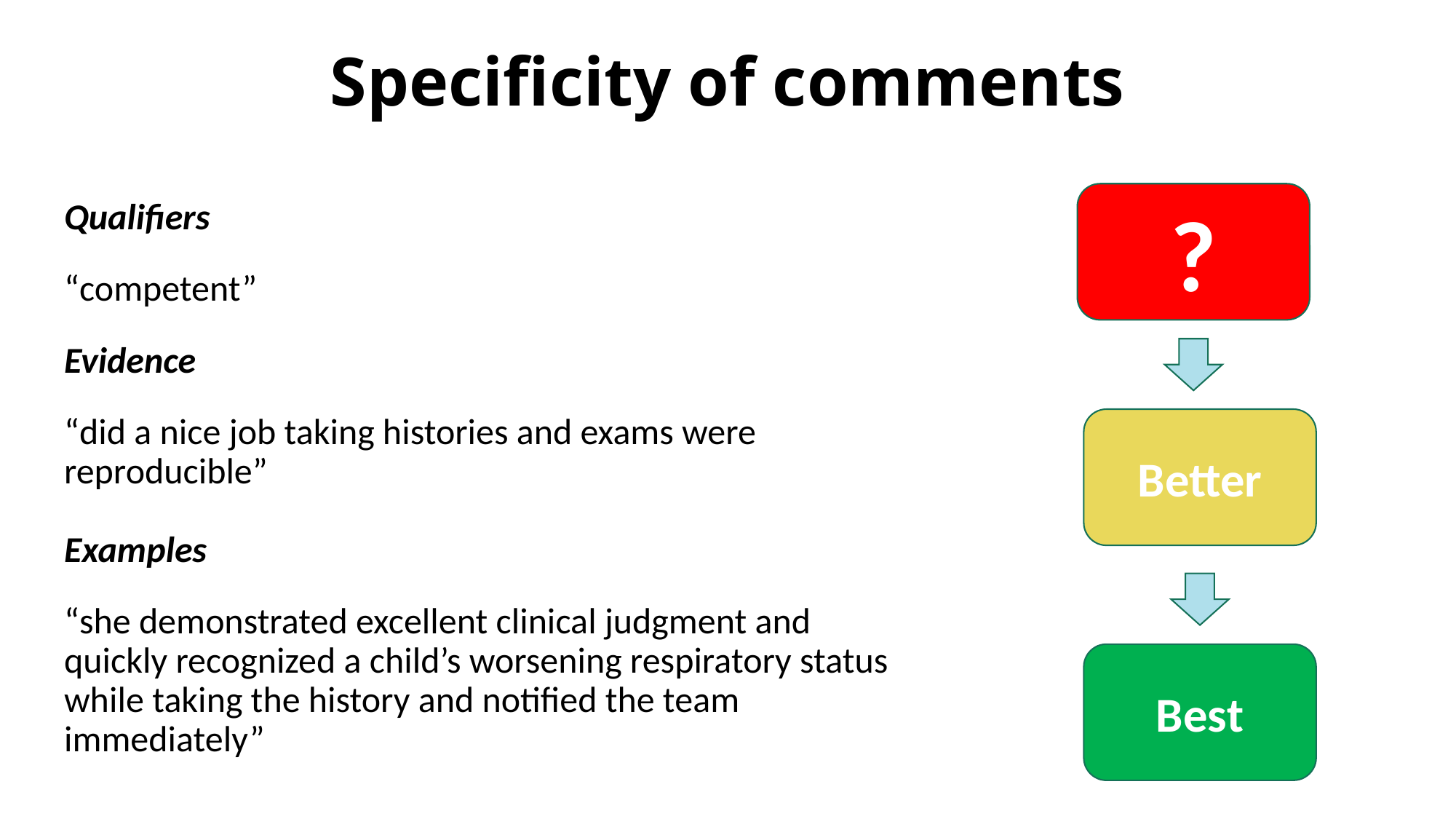

# Specificity of comments
Qualifiers
“competent”
Evidence
“did a nice job taking histories and exams were reproducible”
Examples
“she demonstrated excellent clinical judgment and quickly recognized a child’s worsening respiratory status while taking the history and notified the team immediately”
?
Better
Best

## Slide 14
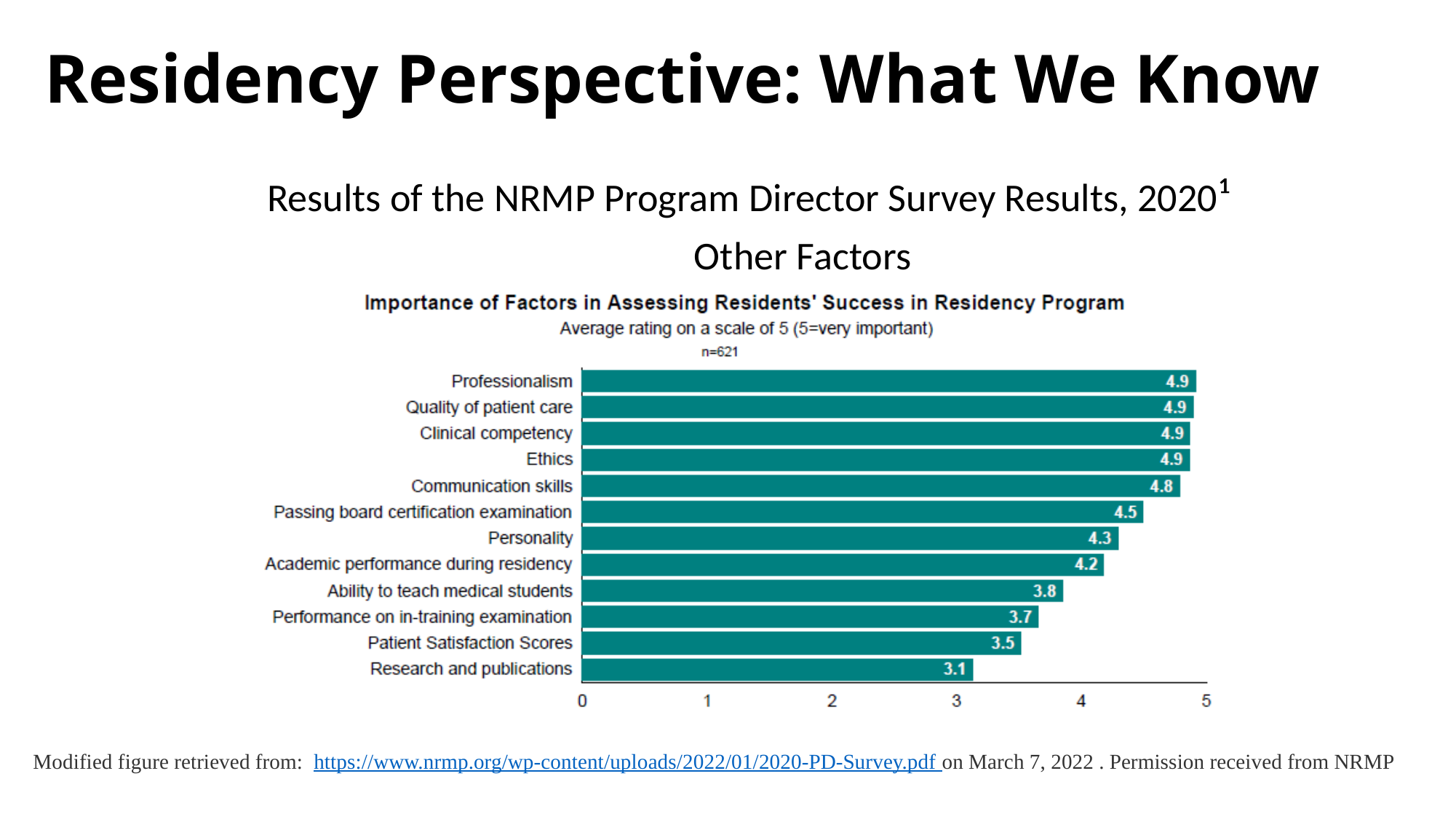

# Residency Perspective: What We Know
Results of the NRMP Program Director Survey Results, 2020¹
	Other Factors
Modified figure retrieved from: https://www.nrmp.org/wp-content/uploads/2022/01/2020-PD-Survey.pdf on March 7, 2022 . Permission received from NRMP

## Slide 15
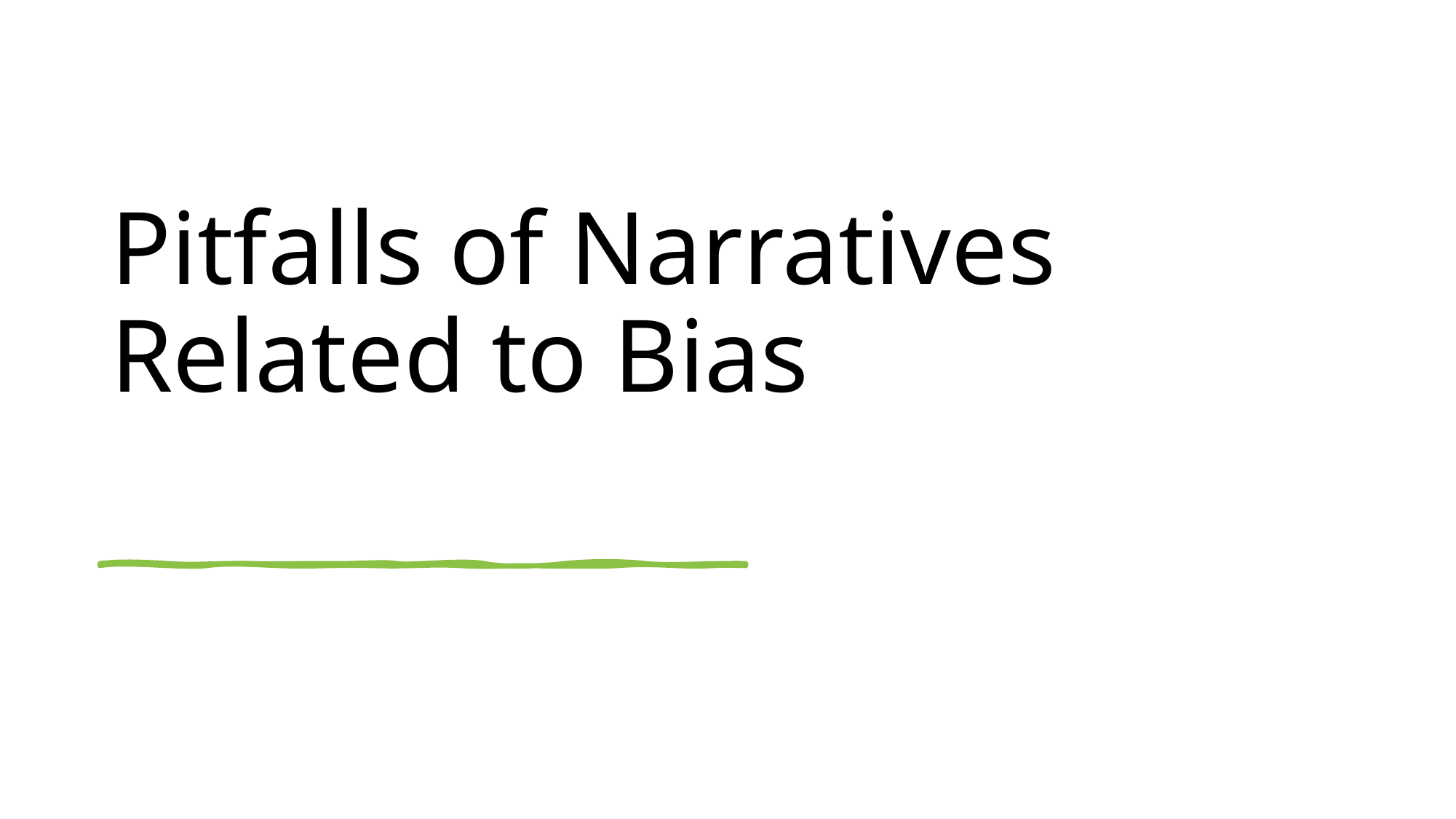

# Pitfalls of Narratives Related to Bias

## Slide 16
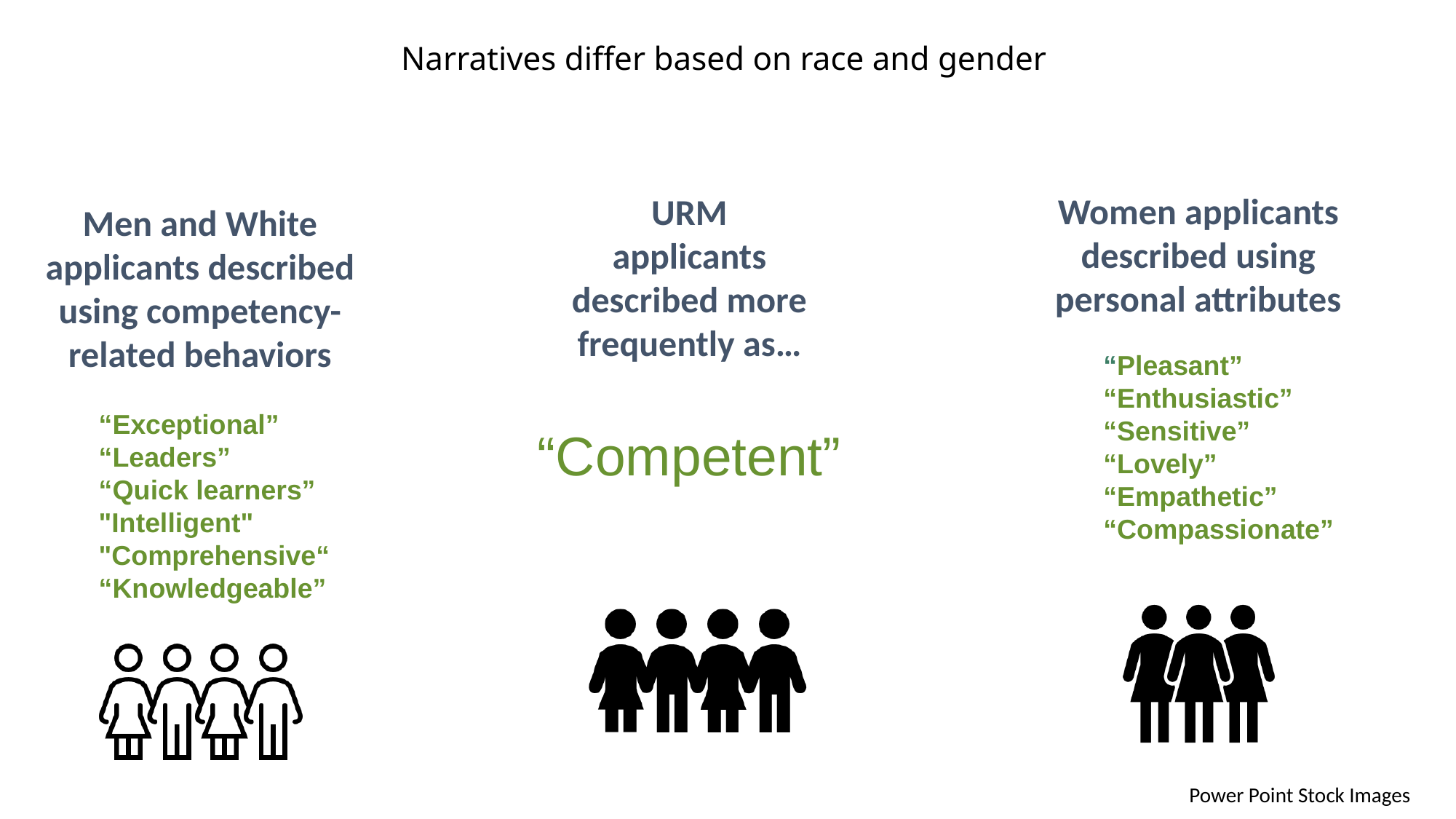

# Narratives differ based on race and gender
Women applicants described using personal attributes
URM applicants described more frequently as…
Men and White applicants described using competency-related behaviors
 “Pleasant”
 “Enthusiastic”
 “Sensitive”
 “Lovely”
 “Empathetic”
 “Compassionate”
“Exceptional”
“Leaders”
“Quick learners”
"Intelligent"
"Comprehensive“
“Knowledgeable”
“Competent”
Power Point Stock Images

## Slide 17
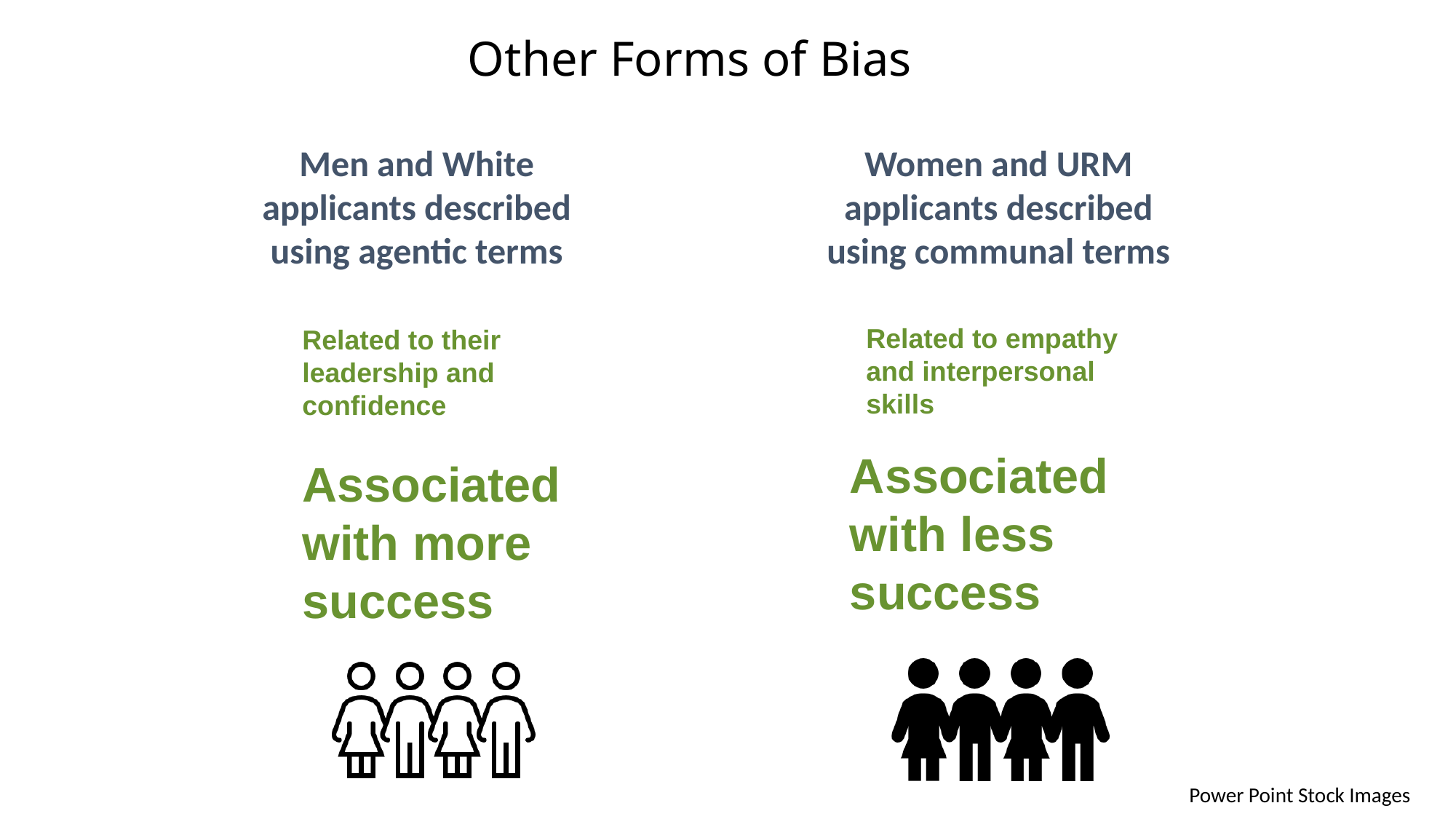

# Other Forms of Bias
Men and White applicants described using agentic terms
Women and URM applicants described using communal terms
Related to empathy and interpersonal skills
Related to their leadership and confidence
Associated with more success
Associated with less success
Power Point Stock Images

## Slide 18
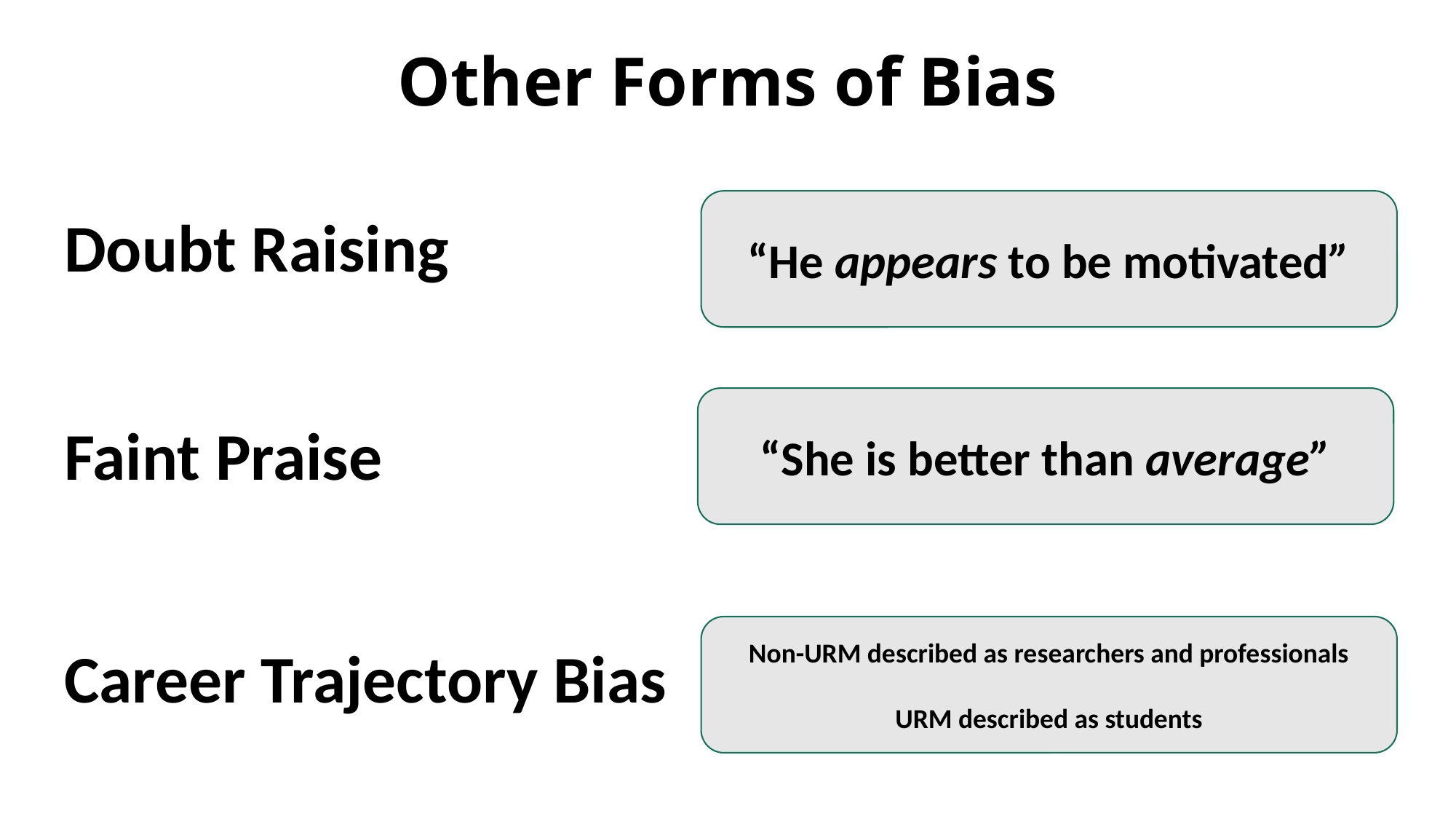

# Other Forms of Bias
“He appears to be motivated”
Doubt Raising
Faint Praise
Career Trajectory Bias
“She is better than average”
Non-URM described as researchers and professionals
URM described as students

## Slide 19
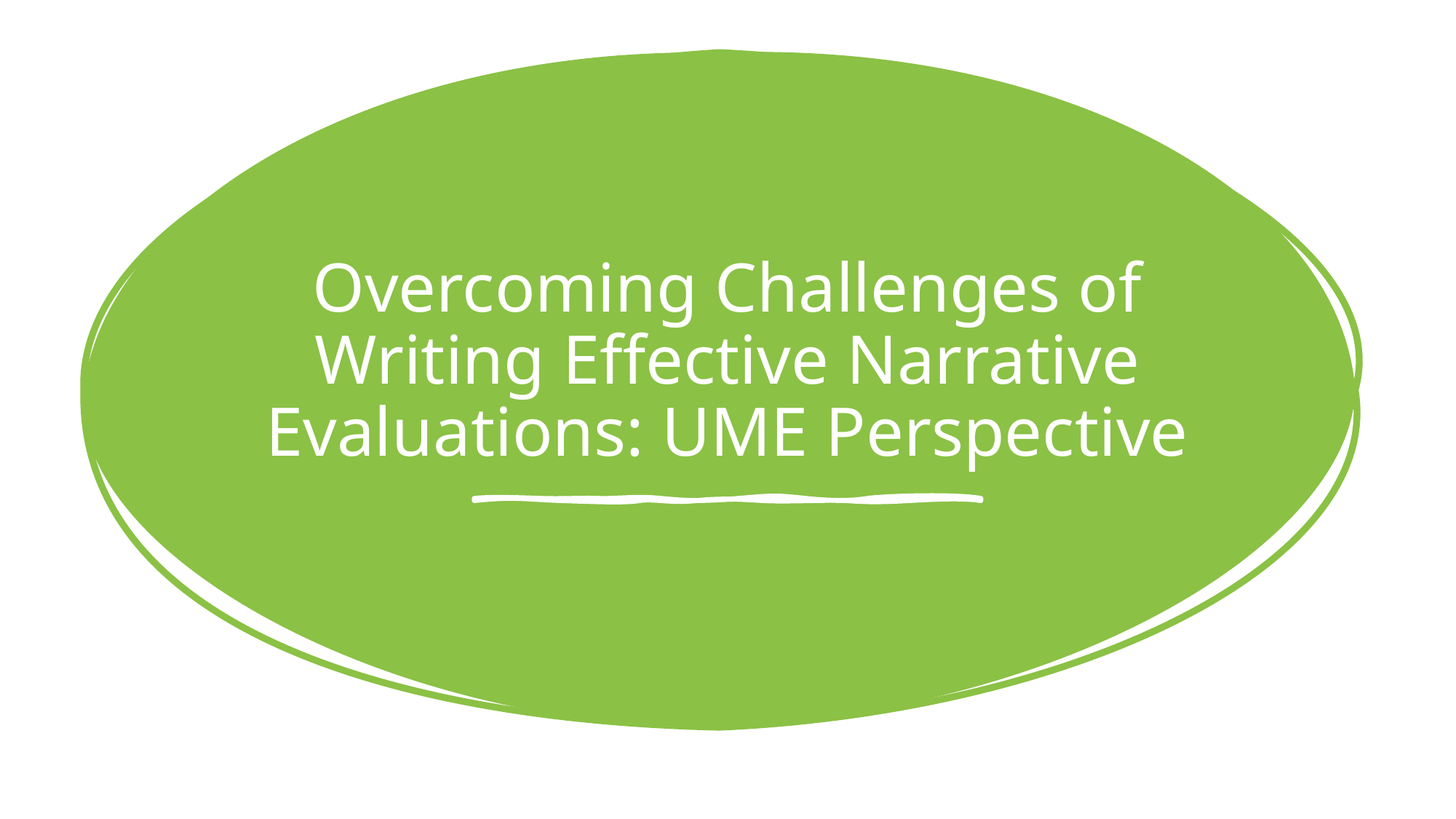

# Overcoming Challenges of Writing Effective Narrative Evaluations: UME Perspective

## Slide 20
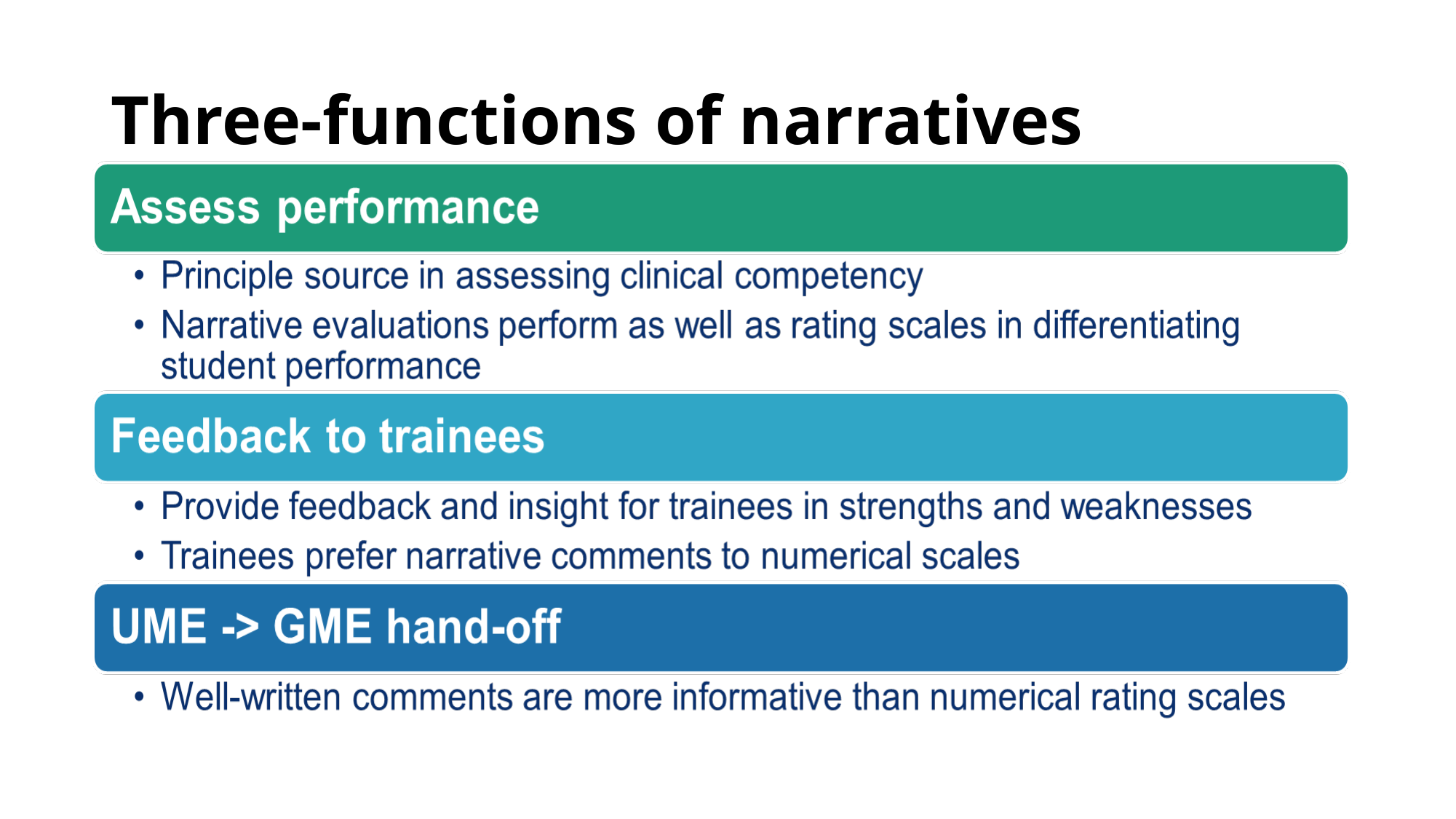

# Three-functions of narratives

## Slide 21
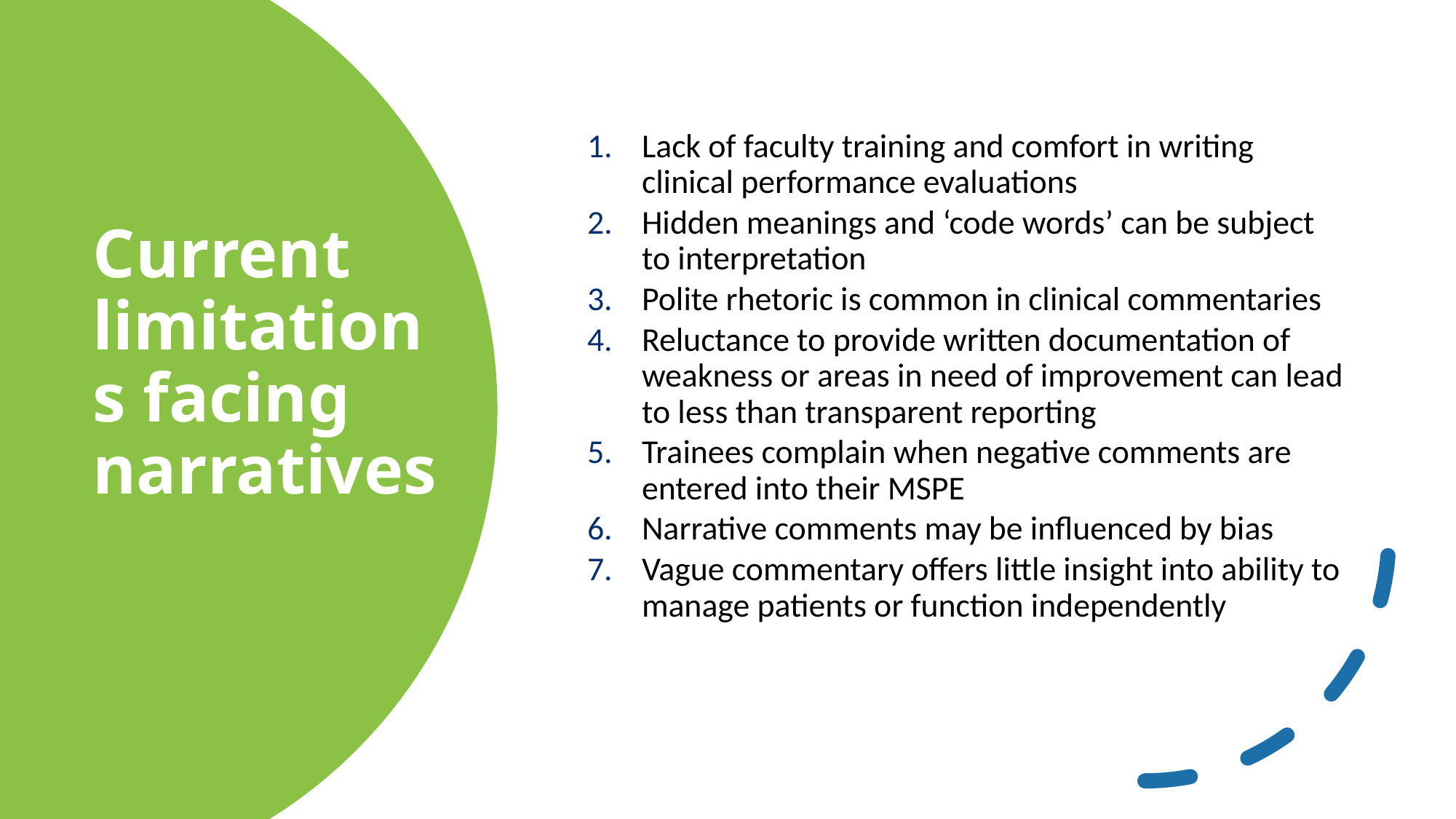

Lack of faculty training and comfort in writing clinical performance evaluations
Hidden meanings and ‘code words’ can be subject to interpretation
Polite rhetoric is common in clinical commentaries
Reluctance to provide written documentation of weakness or areas in need of improvement can lead to less than transparent reporting
Trainees complain when negative comments are entered into their MSPE
Narrative comments may be influenced by bias
Vague commentary offers little insight into ability to manage patients or function independently
# Current limitations facing narratives

## Slide 22
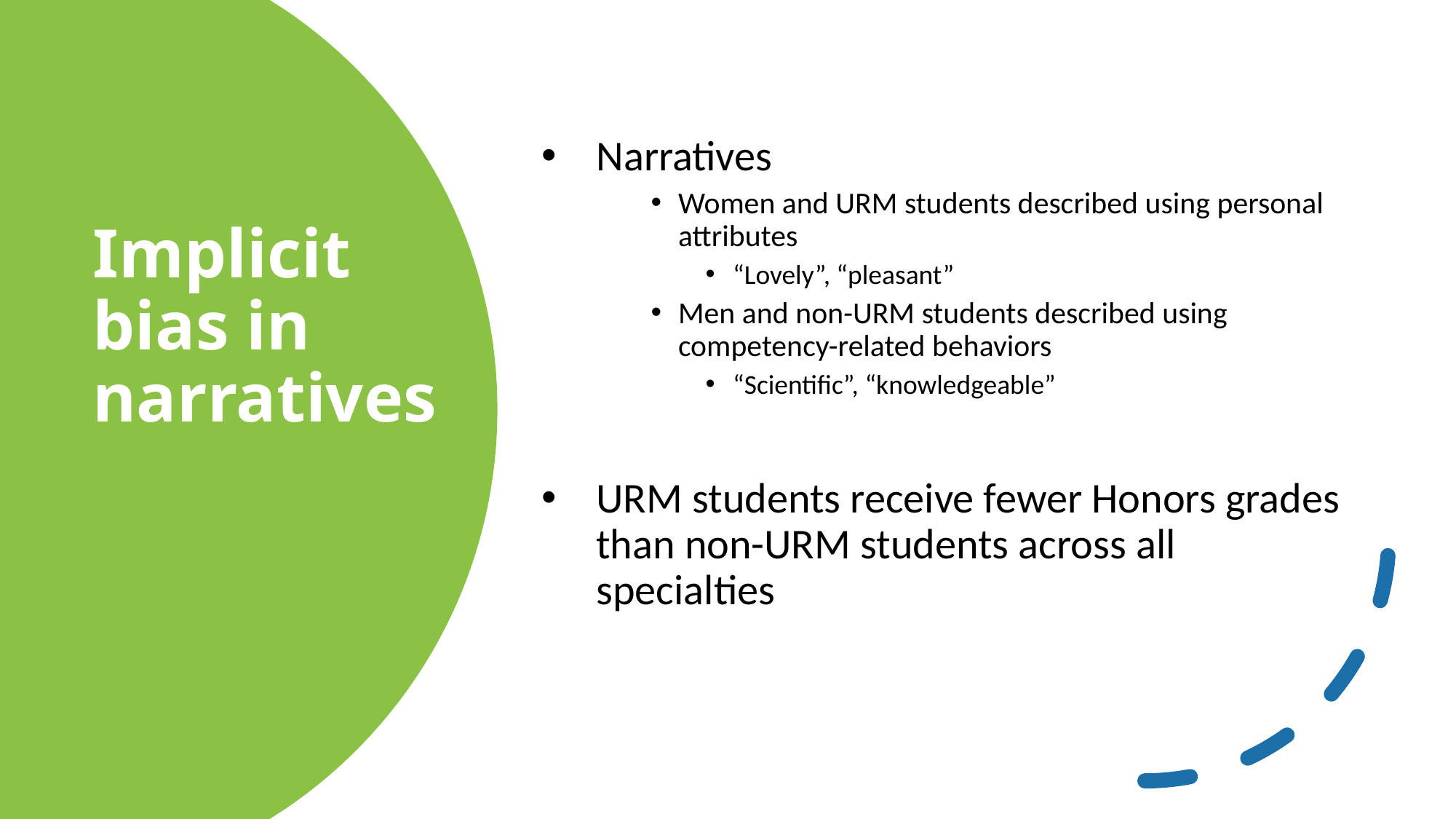

Narratives
Women and URM students described using personal attributes
“Lovely”, “pleasant”
Men and non-URM students described using competency-related behaviors
“Scientific”, “knowledgeable”
URM students receive fewer Honors grades than non-URM students across all specialties
# Implicit bias in narratives

## Slide 23
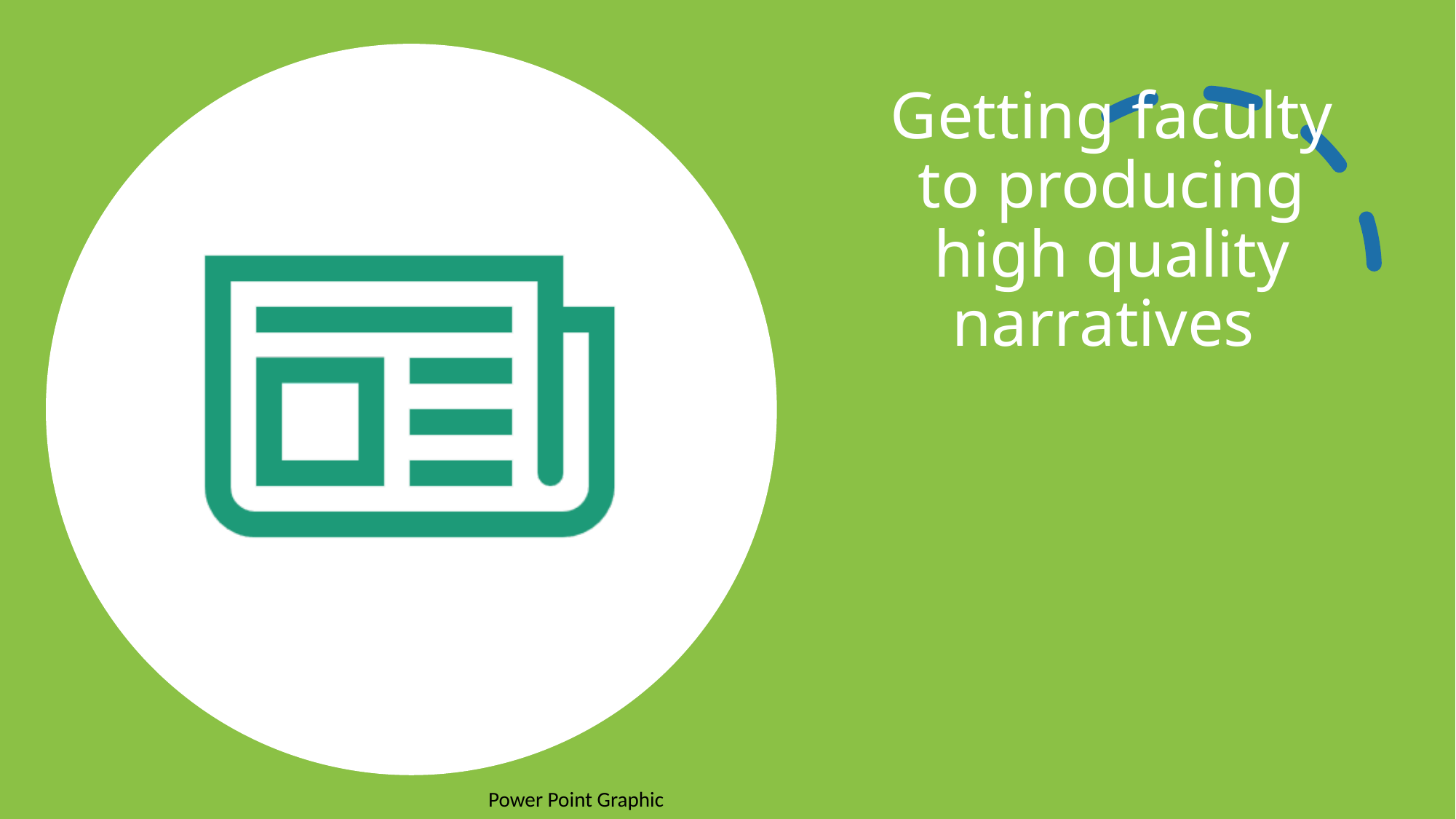

# Getting faculty to producing high quality narratives
Power Point Graphic

## Slide 24
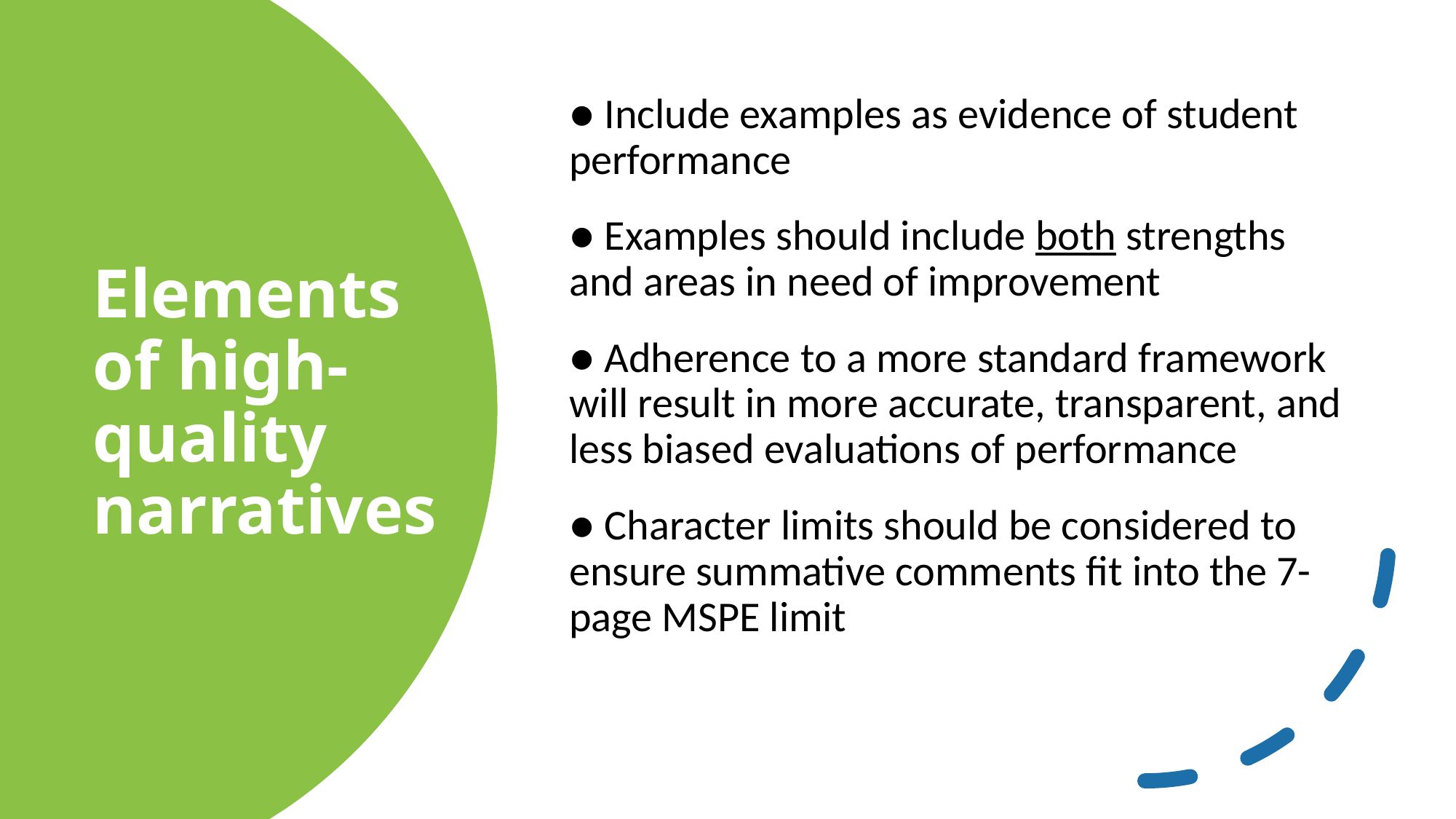

● Include examples as evidence of student performance
● Examples should include both strengths and areas in need of improvement
● Adherence to a more standard framework will result in more accurate, transparent, and less biased evaluations of performance
● Character limits should be considered to ensure summative comments fit into the 7-page MSPE limit
# Elements of high-quality narratives

## Slide 25
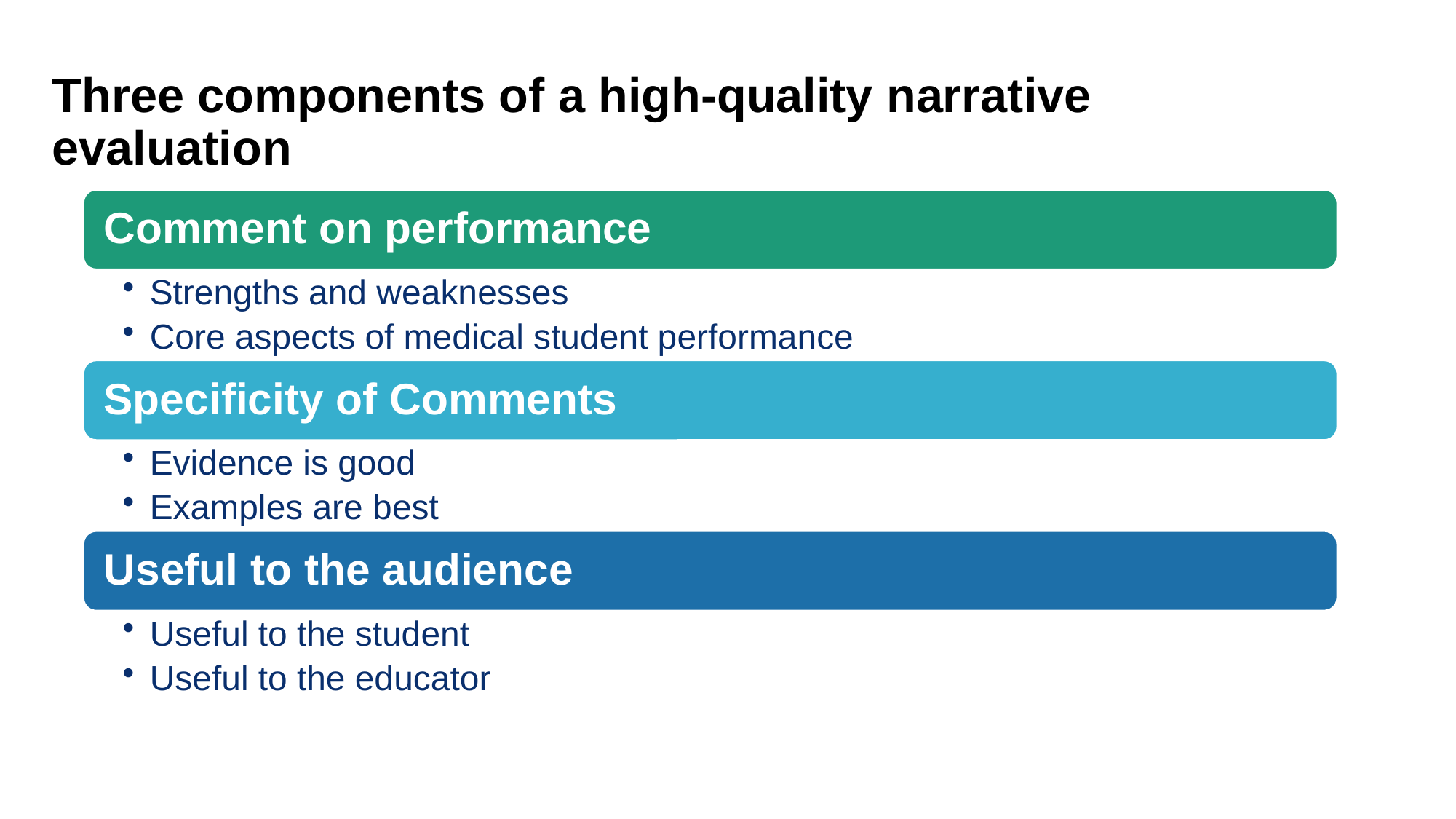

# Three components of a high-quality narrative evaluation

## Slide 26
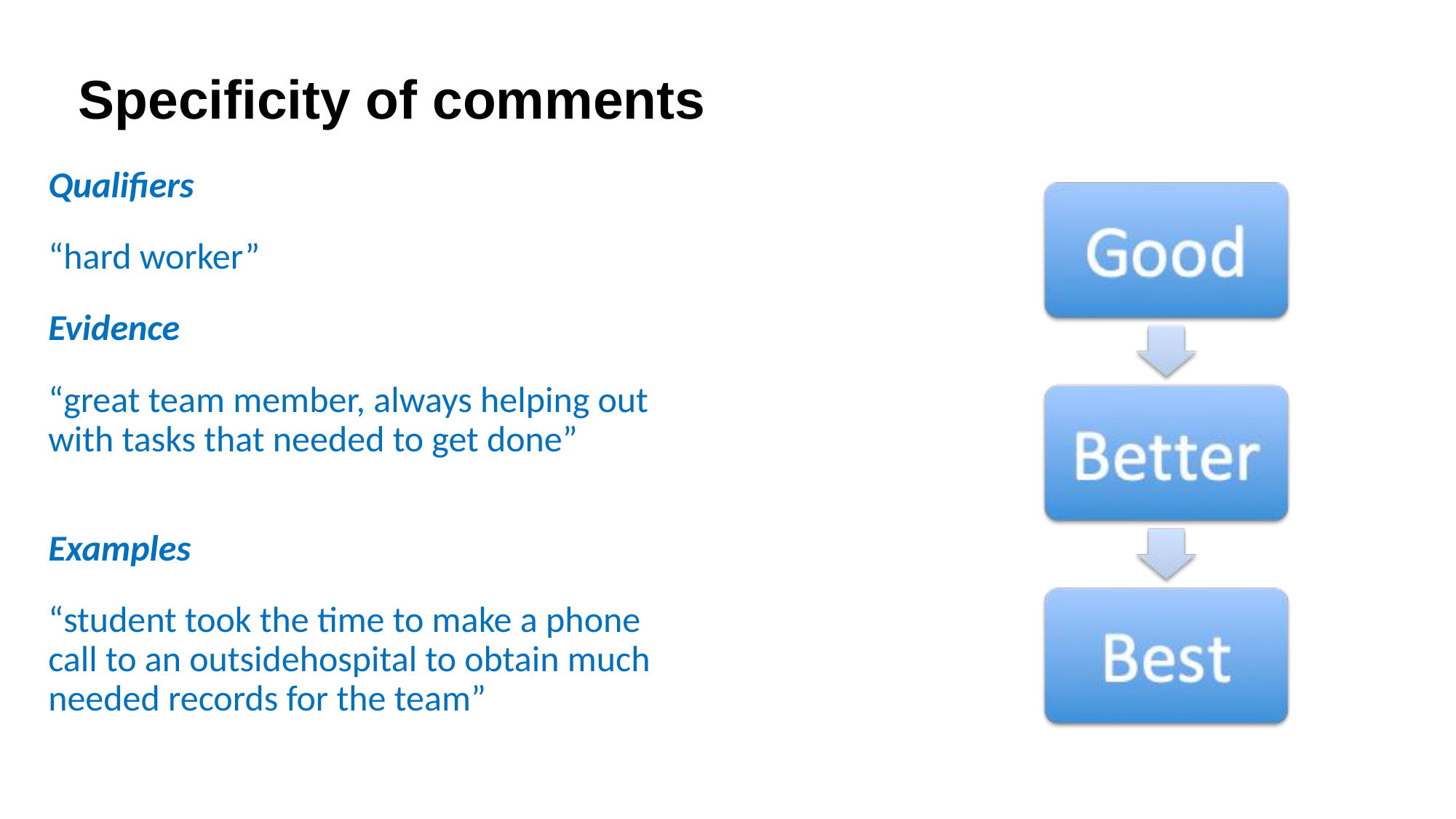

# Specificity of comments
Qualifiers
“hard worker”
Evidence
“great team member, always helping out with tasks that needed to get done”
Examples
“student took the time to make a phone call to an outsidehospital to obtain much needed records for the team”

## Slide 27
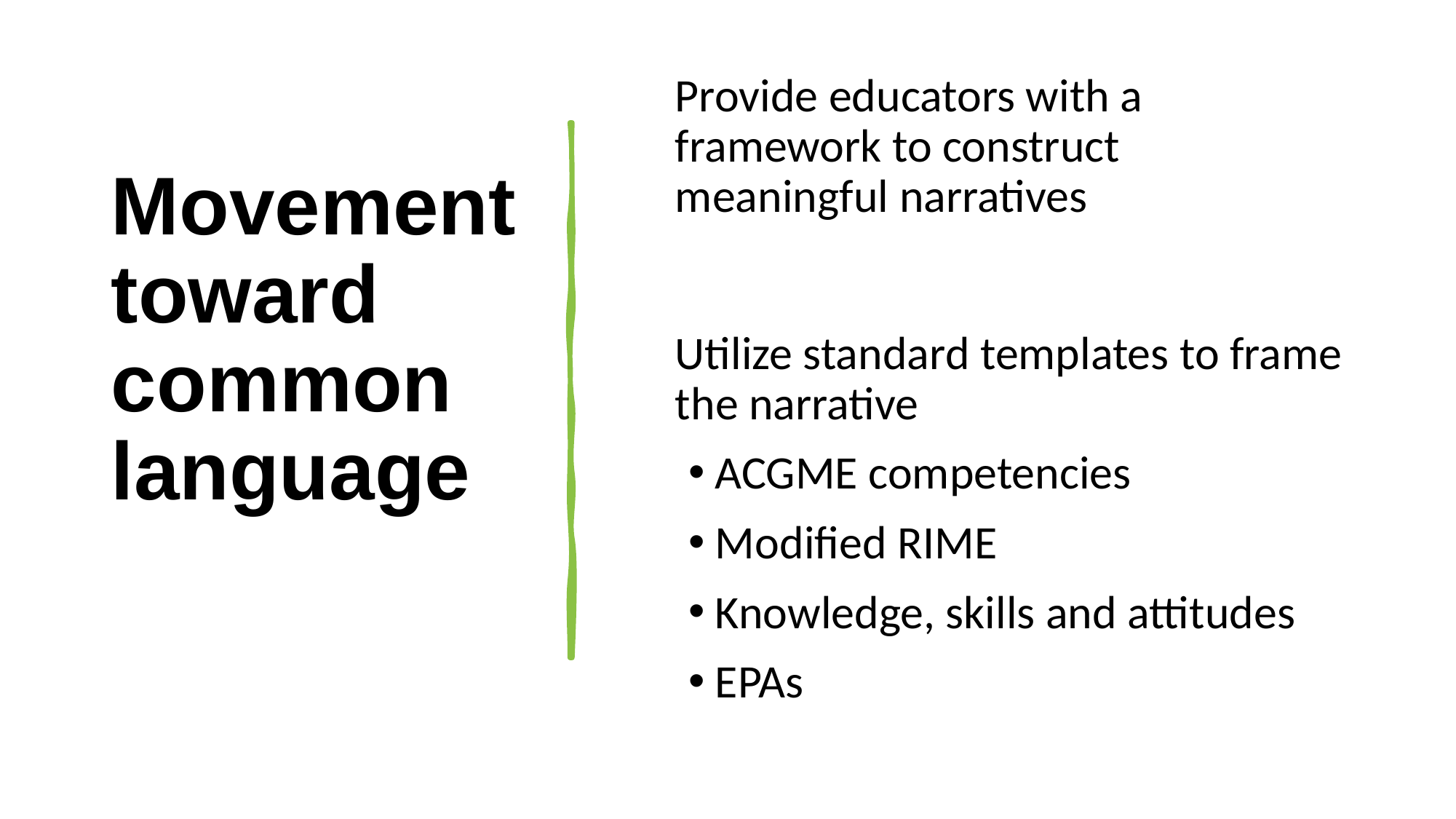

# Movement toward common language
Provide educators with a framework to construct meaningful narratives
Utilize standard templates to frame the narrative
ACGME competencies
Modified RIME
Knowledge, skills and attitudes
EPAs

## Slide 28
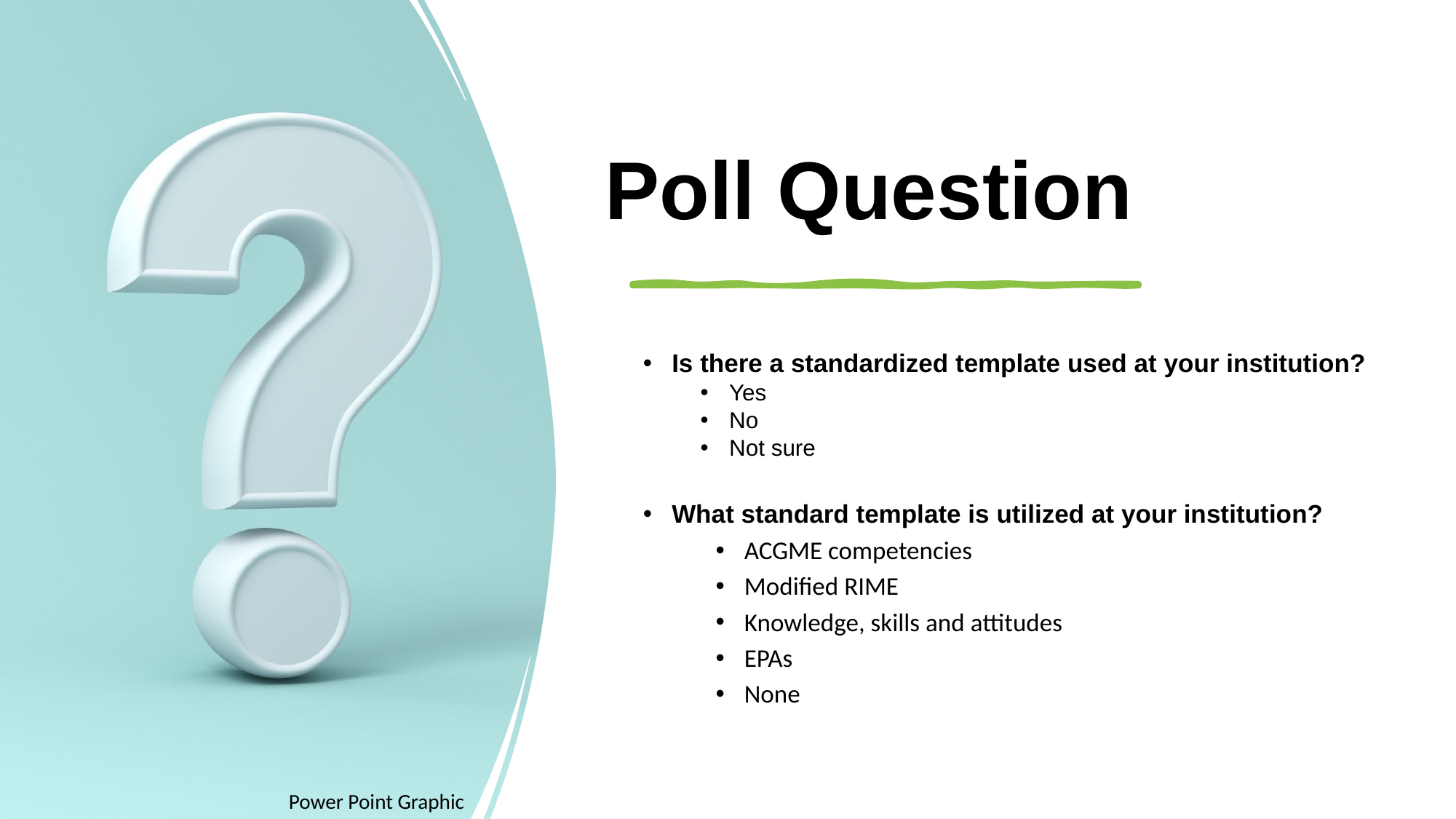

# Poll Question
Is there a standardized template used at your institution?
Yes
No
Not sure
What standard template is utilized at your institution?
ACGME competencies
Modified RIME
Knowledge, skills and attitudes
EPAs
None
Power Point Graphic

## Slide 29
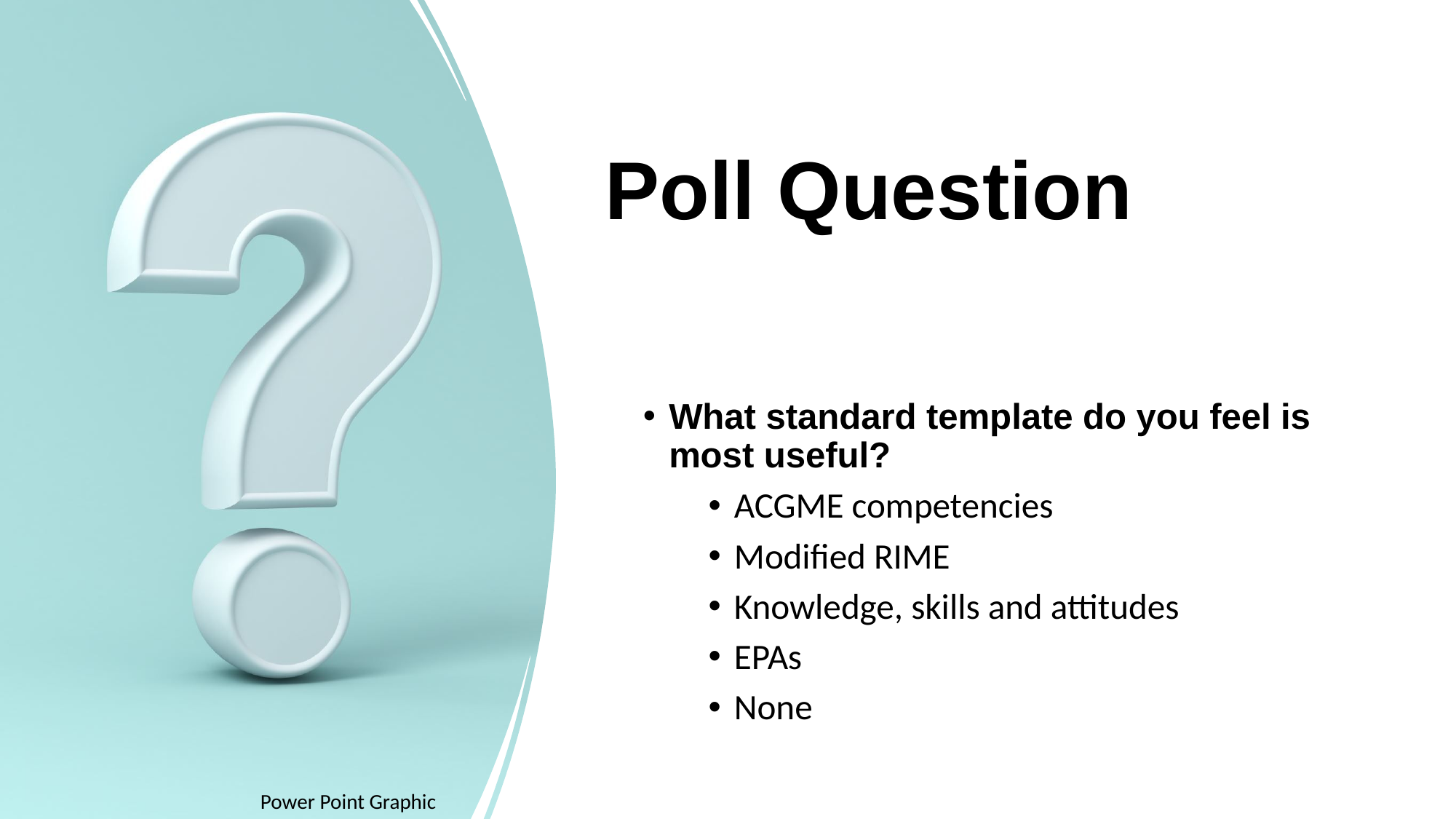

# Poll Question
What standard template do you feel is most useful?
ACGME competencies
Modified RIME
Knowledge, skills and attitudes
EPAs
None
Power Point Graphic

## Slide 30
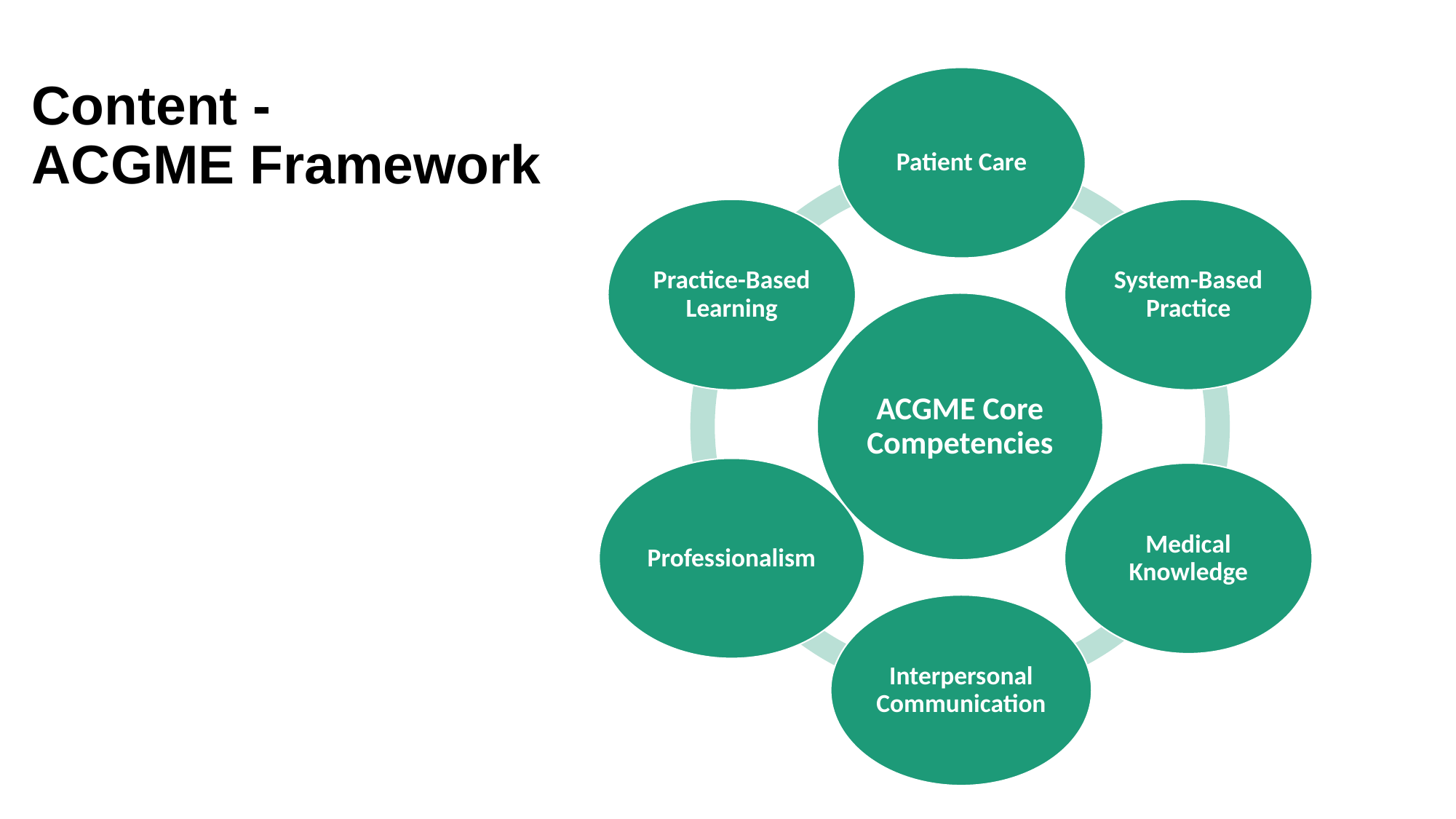

# Content - ACGME Framework

## Slide 31
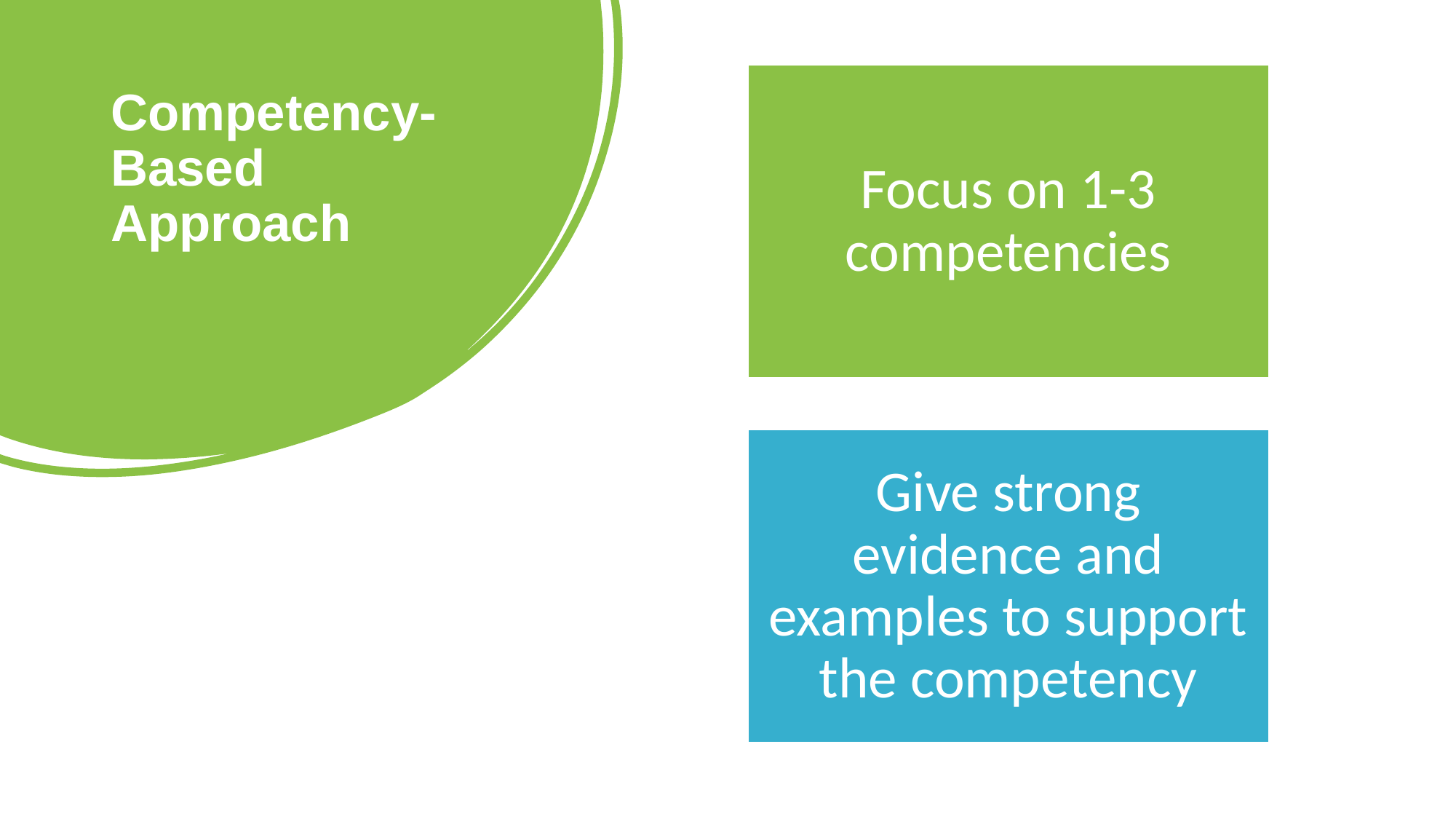

# Competency-Based Approach

## Slide 32
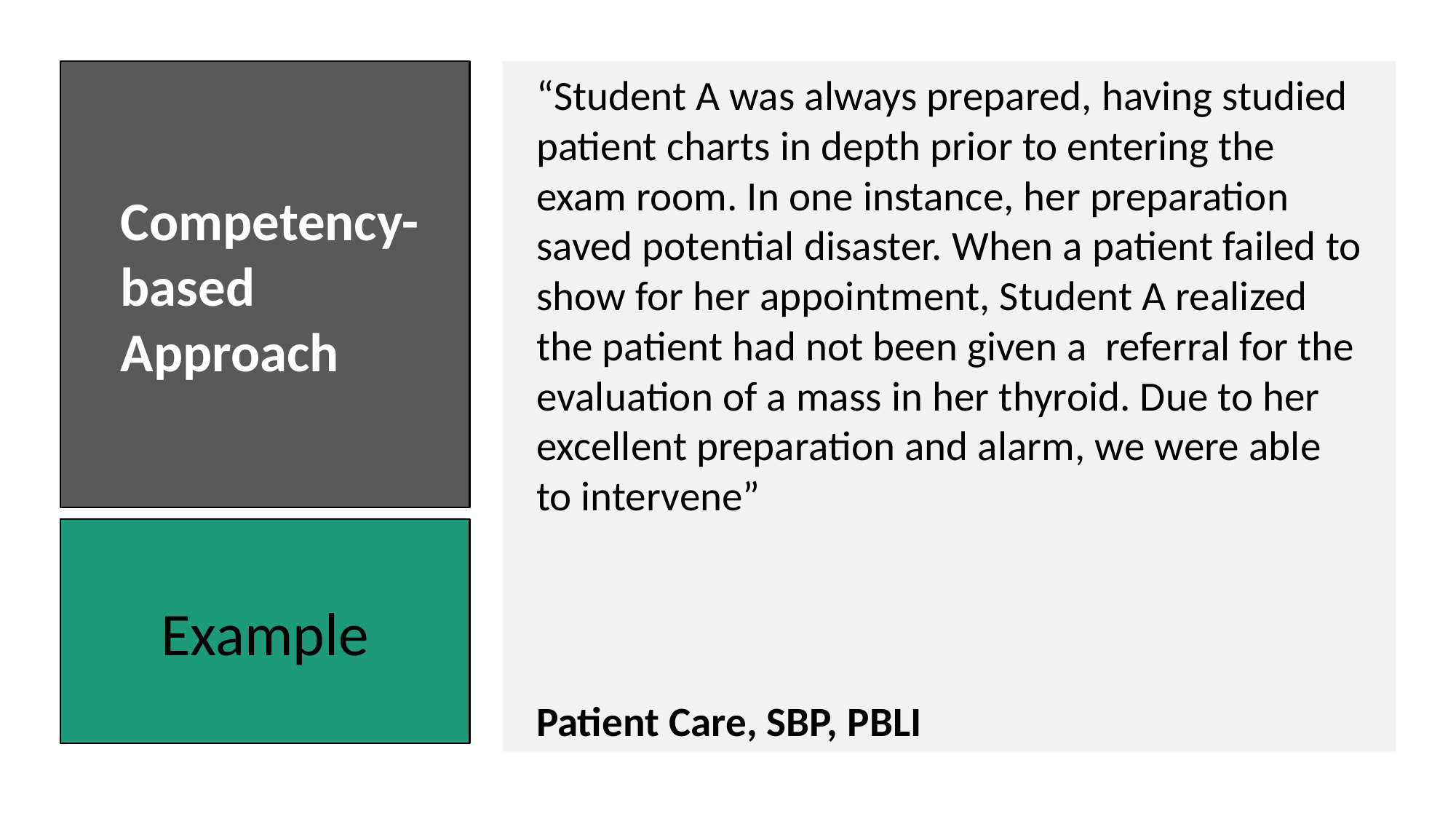

Competency-based Approach
“Student A was always prepared, having studied patient charts in depth prior to entering the exam room. In one instance, her preparation saved potential disaster. When a patient failed to show for her appointment, Student A realized the patient had not been given a referral for the evaluation of a mass in her thyroid. Due to her excellent preparation and alarm, we were able to intervene”
Patient Care, SBP, PBLI
Example

## Slide 33
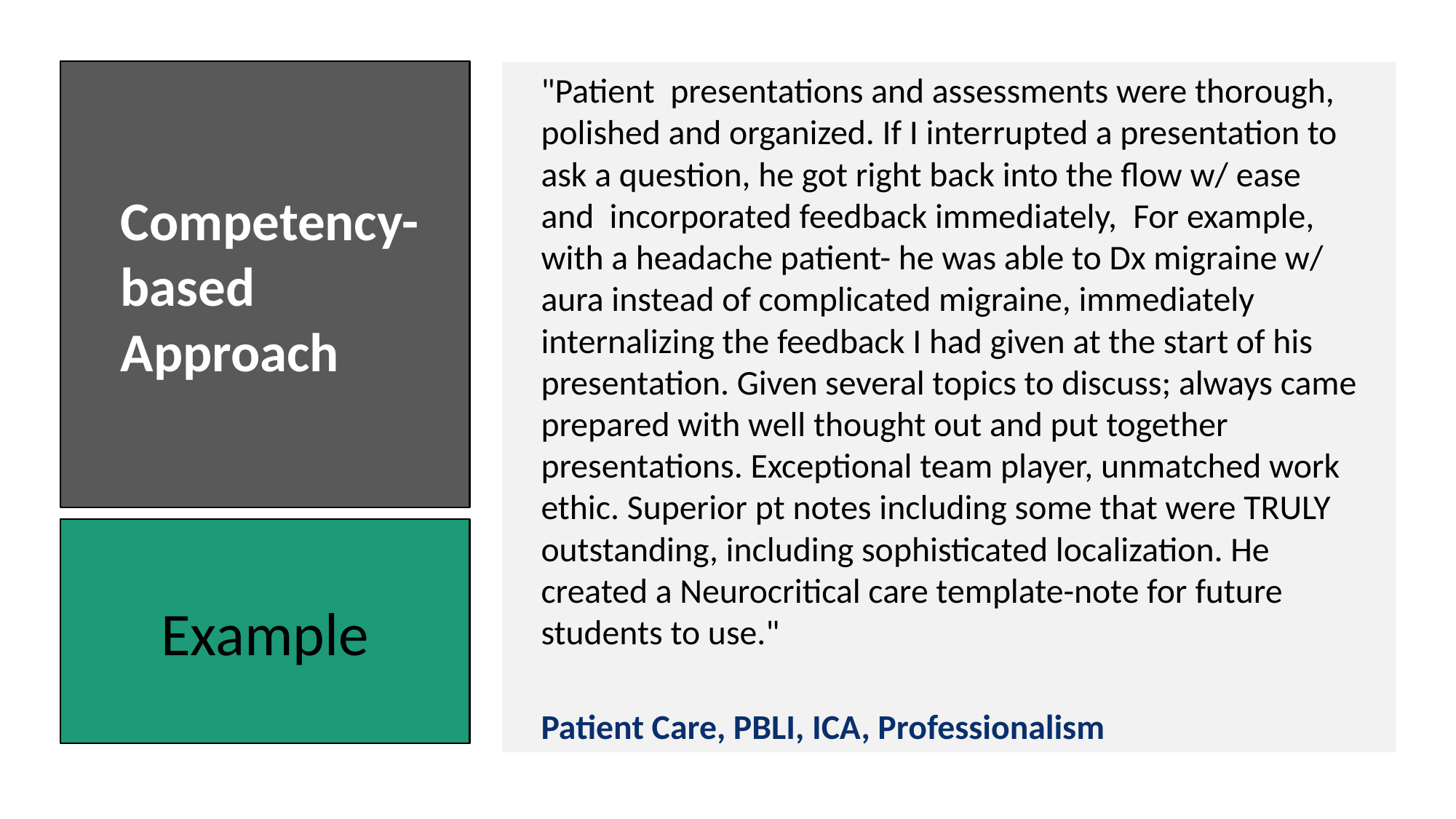

Competency-based Approach
"Patient  presentations and assessments were thorough, polished and organized. If I interrupted a presentation to ask a question, he got right back into the flow w/ ease and  incorporated feedback immediately,  For example, with a headache patient- he was able to Dx migraine w/ aura instead of complicated migraine, immediately internalizing the feedback I had given at the start of his presentation. Given several topics to discuss; always came prepared with well thought out and put together presentations. Exceptional team player, unmatched work ethic. Superior pt notes including some that were TRULY outstanding, including sophisticated localization. He created a Neurocritical care template-note for future students to use."
Patient Care, PBLI, ICA, Professionalism
Example

## Slide 34
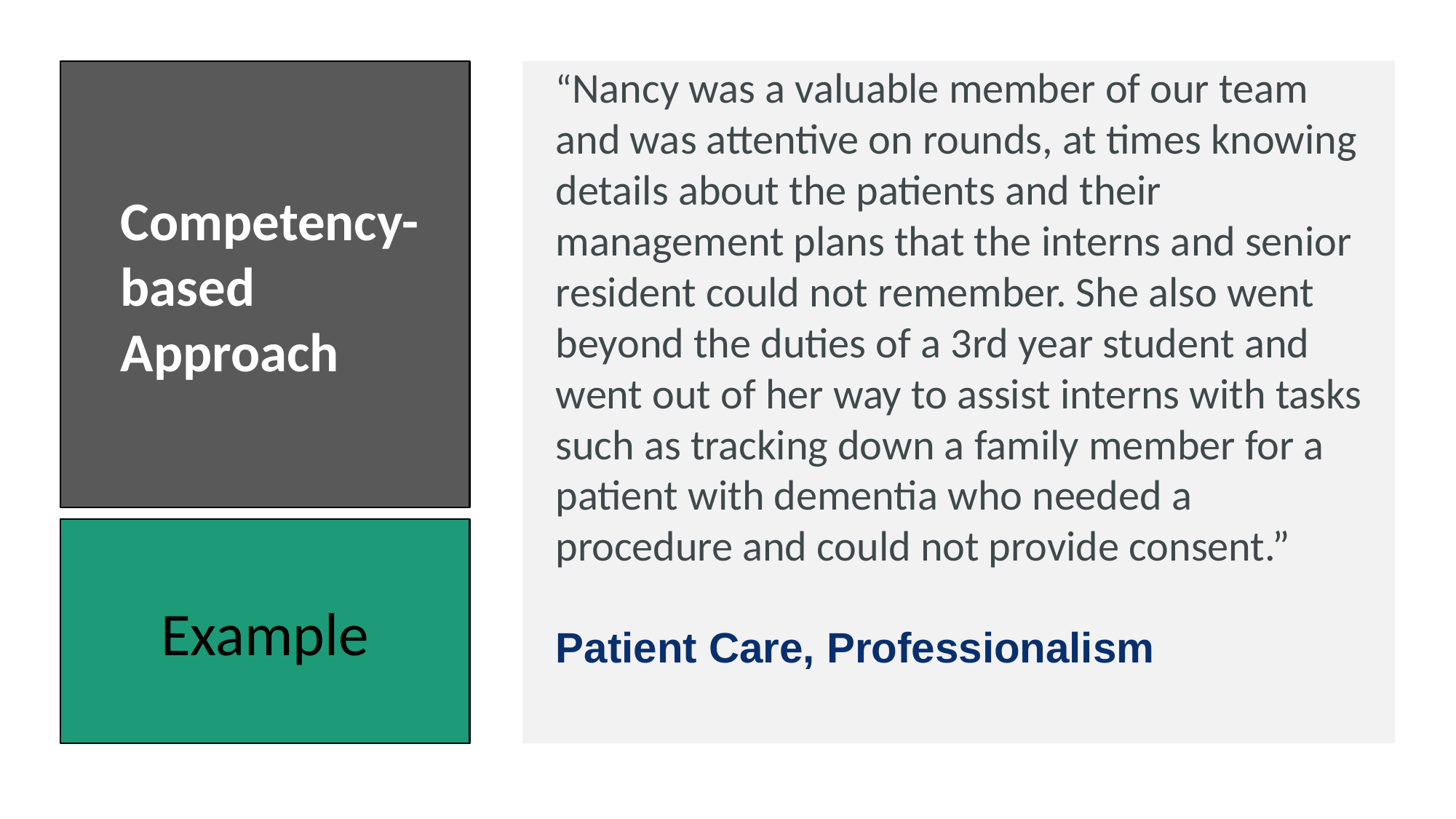

Competency-based Approach
“Nancy was a valuable member of our team and was attentive on rounds, at times knowing details about the patients and their management plans that the interns and senior resident could not remember. She also went beyond the duties of a 3rd year student and went out of her way to assist interns with tasks such as tracking down a family member for a patient with dementia who needed a procedure and could not provide consent.”
Patient Care, Professionalism
Example

## Slide 35
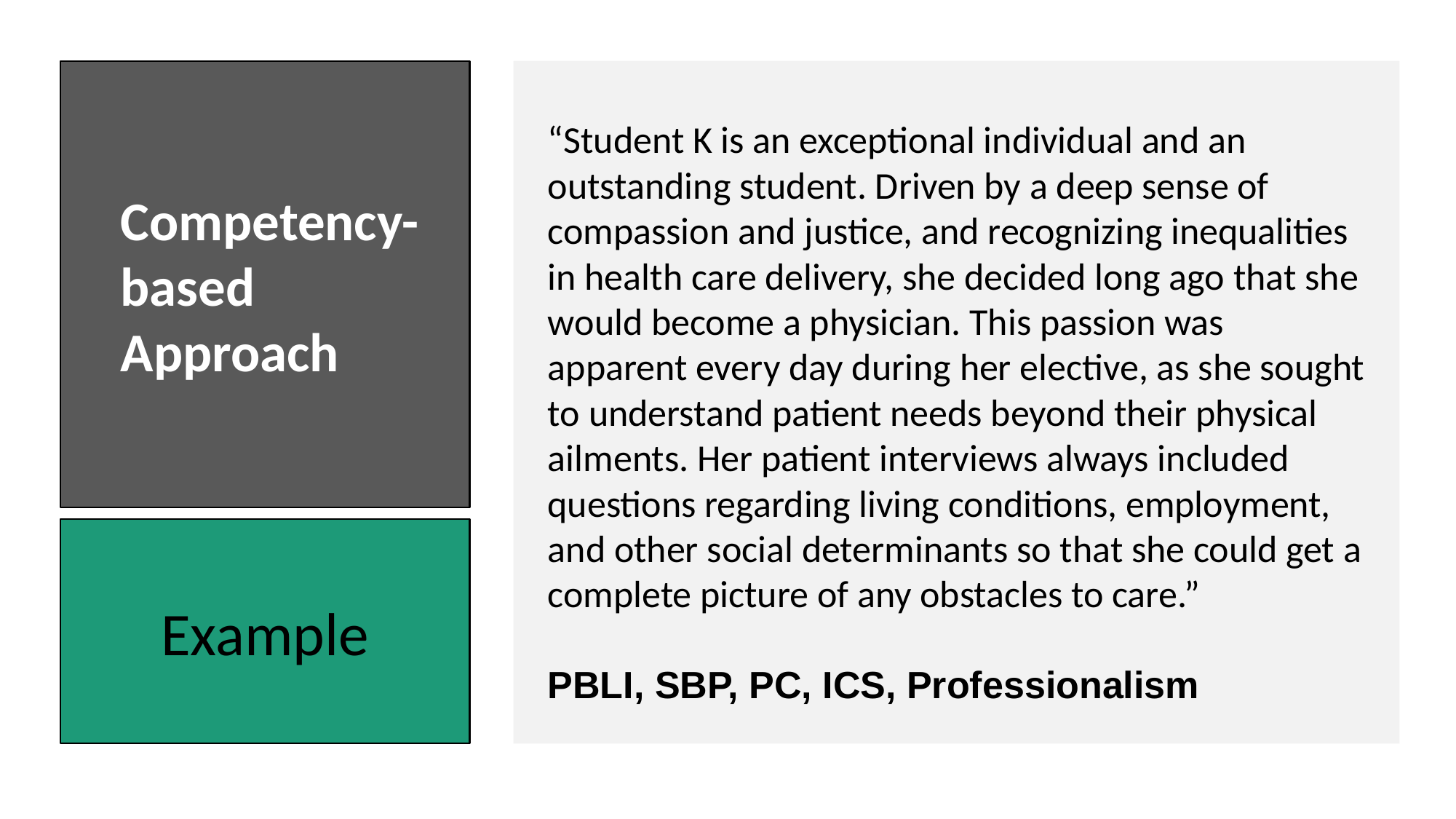

Competency-based Approach
“Student K is an exceptional individual and an outstanding student. Driven by a deep sense of compassion and justice, and recognizing inequalities in health care delivery, she decided long ago that she would become a physician. This passion was apparent every day during her elective, as she sought to understand patient needs beyond their physical ailments. Her patient interviews always included questions regarding living conditions, employment, and other social determinants so that she could get a complete picture of any obstacles to care.”
PBLI, SBP, PC, ICS, Professionalism
Example

## Slide 36
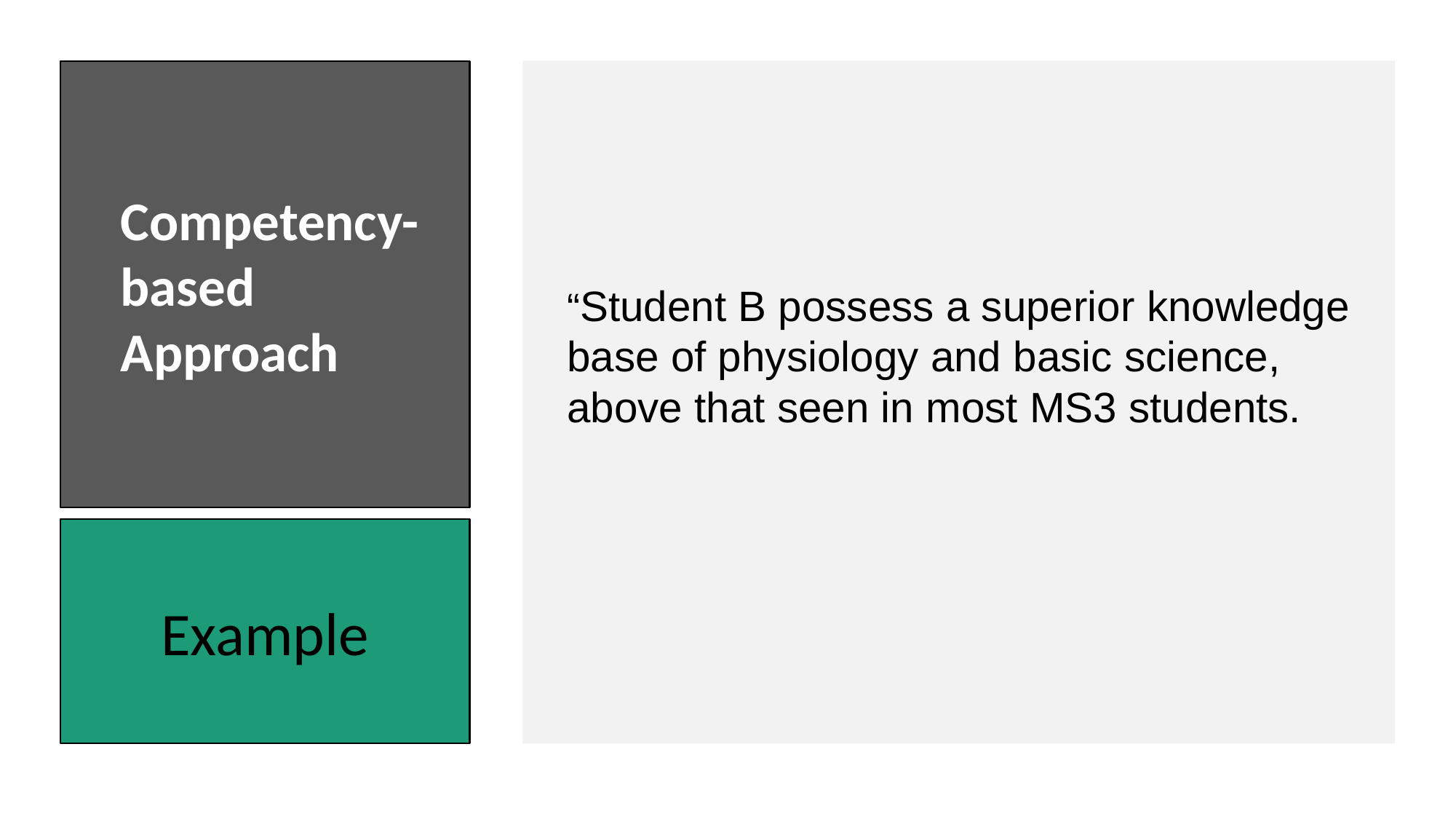

Competency-based Approach
“Student B possess a superior knowledge base of physiology and basic science, above that seen in most MS3 students.
Example

## Slide 37
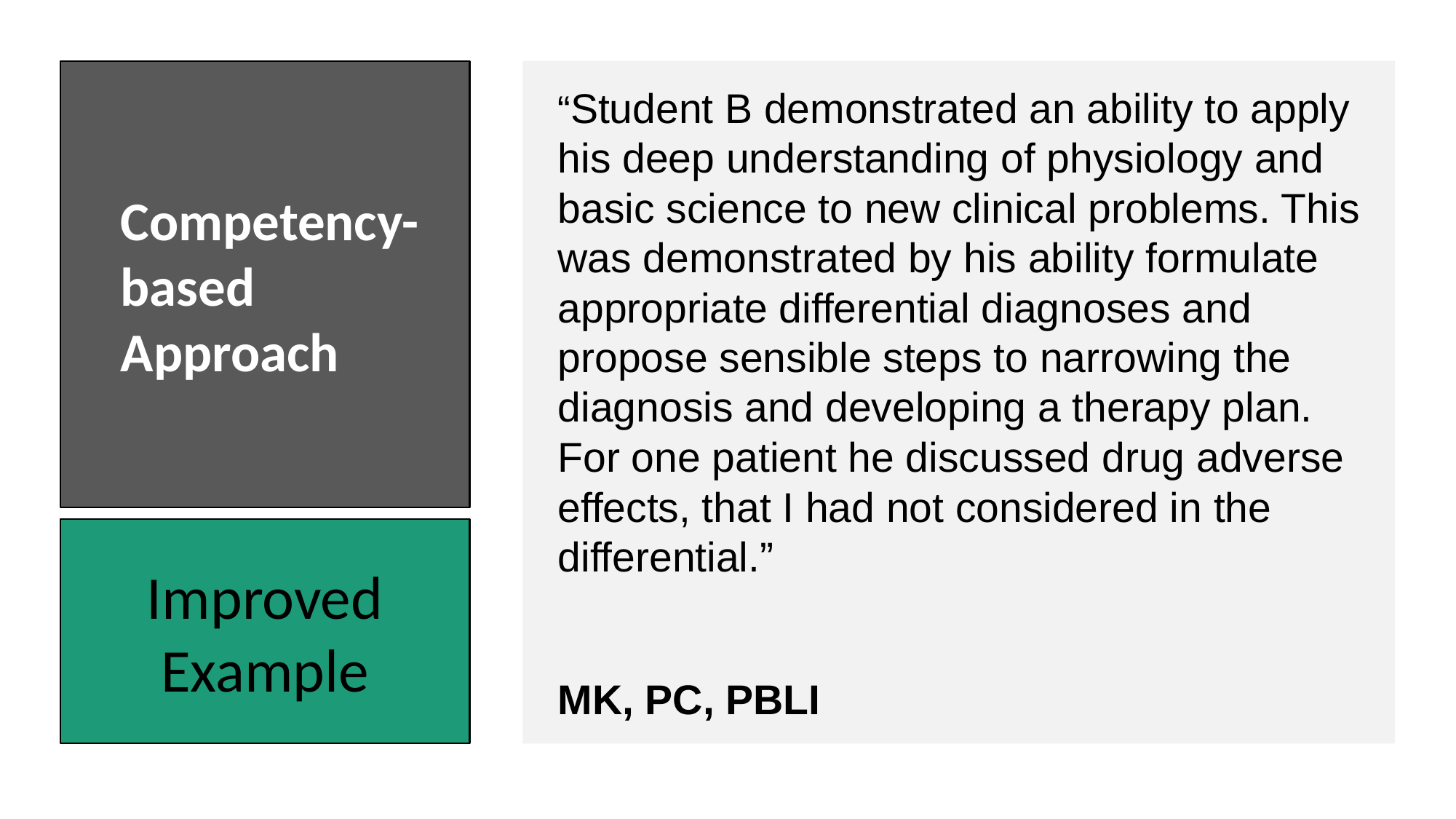

Competency-based Approach
“Student B demonstrated an ability to apply his deep understanding of physiology and basic science to new clinical problems. This was demonstrated by his ability formulate appropriate differential diagnoses and propose sensible steps to narrowing the diagnosis and developing a therapy plan. For one patient he discussed drug adverse effects, that I had not considered in the differential.”
MK, PC, PBLI
Improved Example

## Slide 38
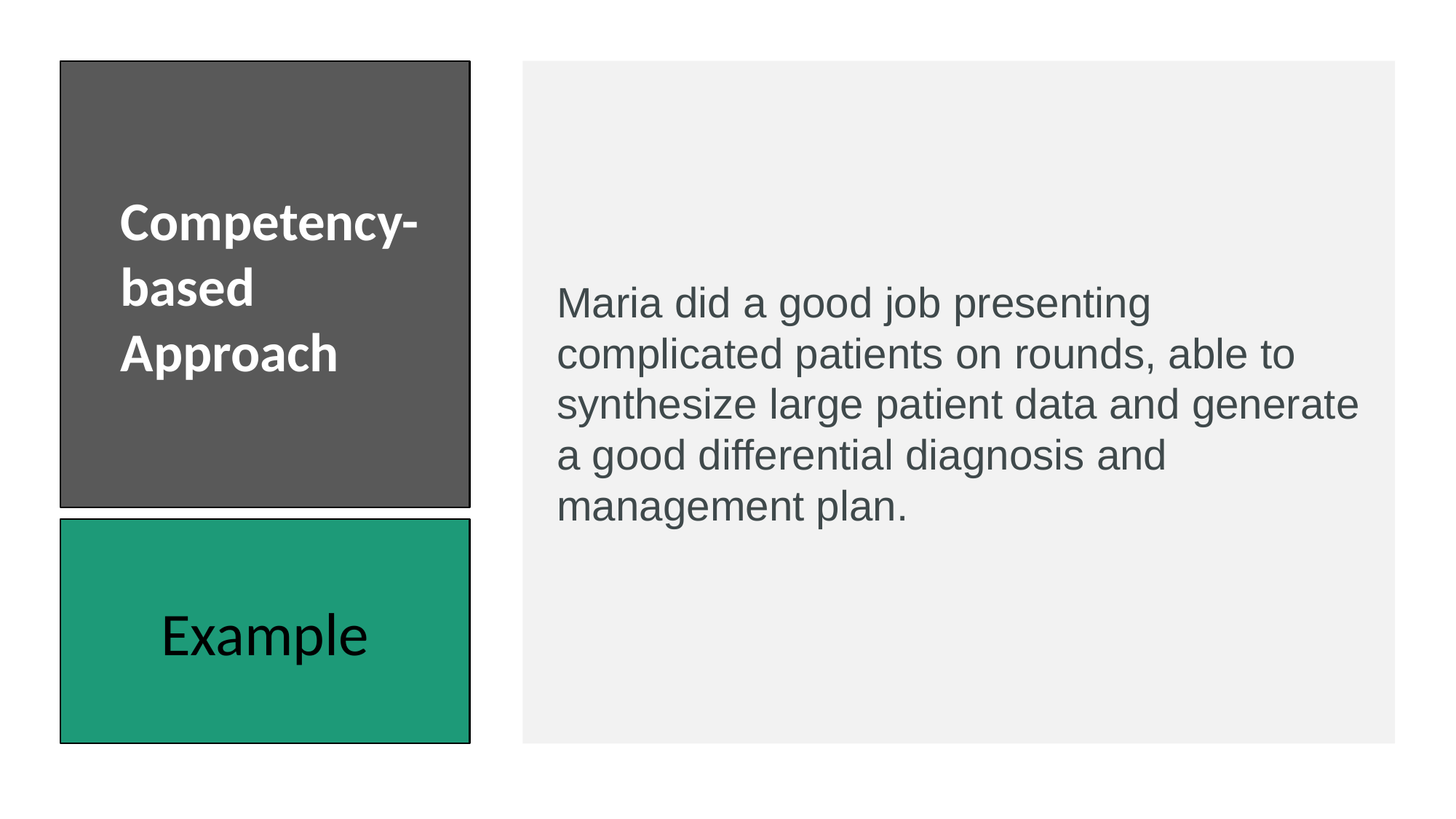

Competency-based Approach
Maria did a good job presenting complicated patients on rounds, able to synthesize large patient data and generate a good differential diagnosis and management plan.
Example

## Slide 39
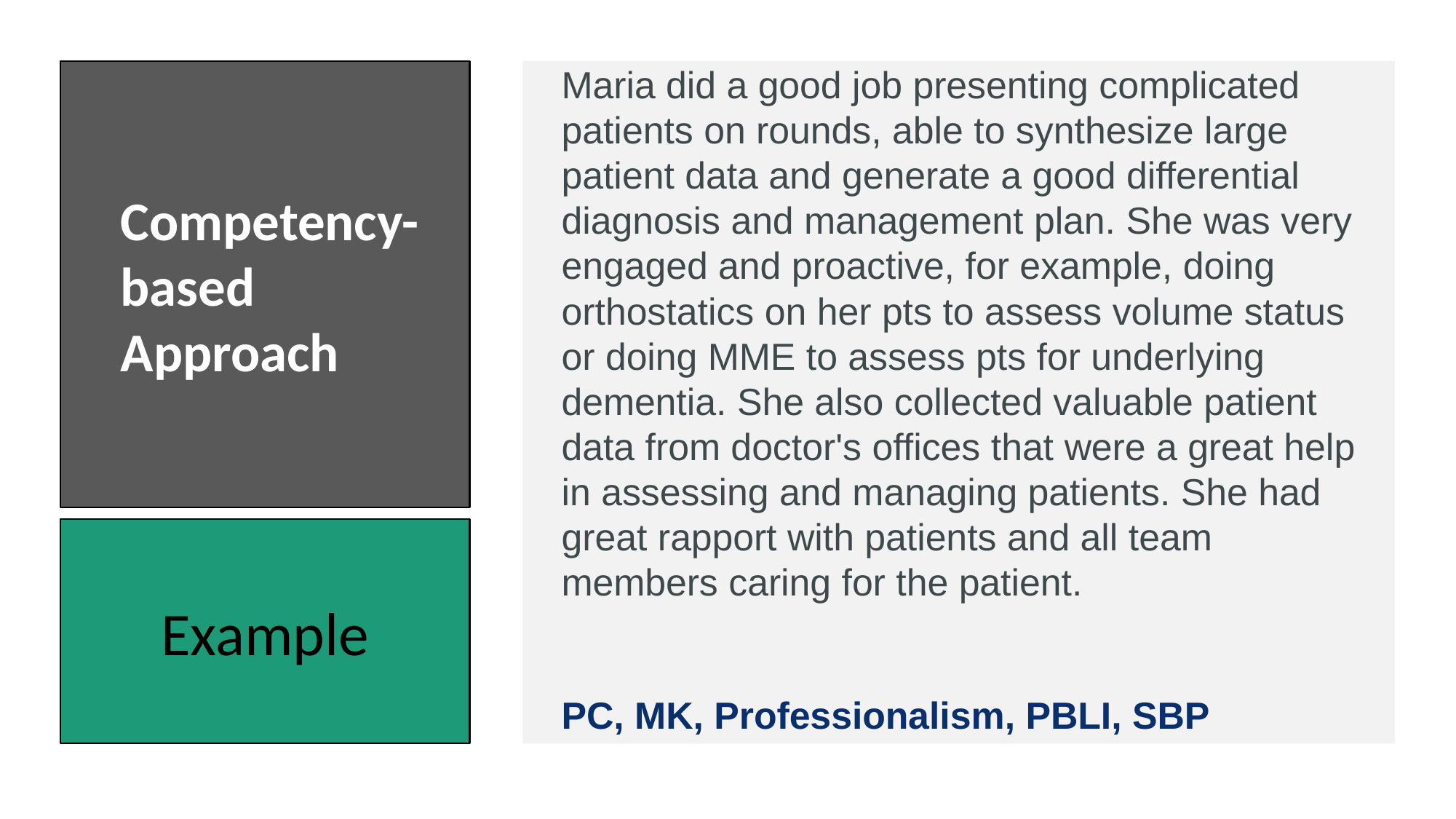

Competency-based Approach
Maria did a good job presenting complicated patients on rounds, able to synthesize large patient data and generate a good differential diagnosis and management plan. She was very engaged and proactive, for example, doing orthostatics on her pts to assess volume status or doing MME to assess pts for underlying dementia. She also collected valuable patient data from doctor's offices that were a great help in assessing and managing patients. She had great rapport with patients and all team members caring for the patient.
PC, MK, Professionalism, PBLI, SBP
Example

## Slide 40
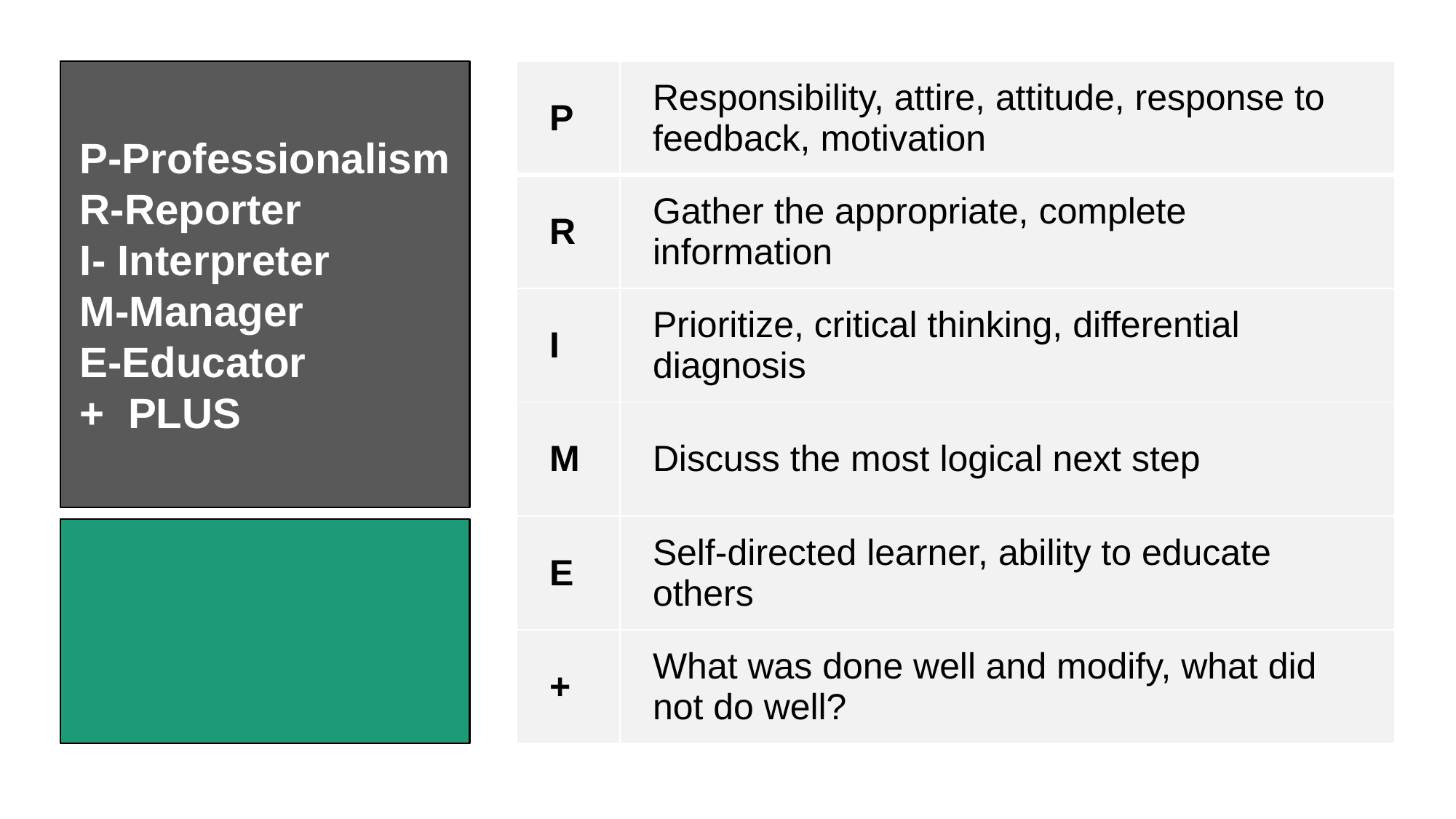

| P | Responsibility, attire, attitude, response to feedback, motivation |
| --- | --- |
| R | Gather the appropriate, complete information |
| I | Prioritize, critical thinking, differential diagnosis |
| M | Discuss the most logical next step |
| E | Self-directed learner, ability to educate others |
| + | What was done well and modify, what did not do well? |
P-Professionalism
R-Reporter
I- Interpreter
M-Manager
E-Educator
+ PLUS

## Slide 41
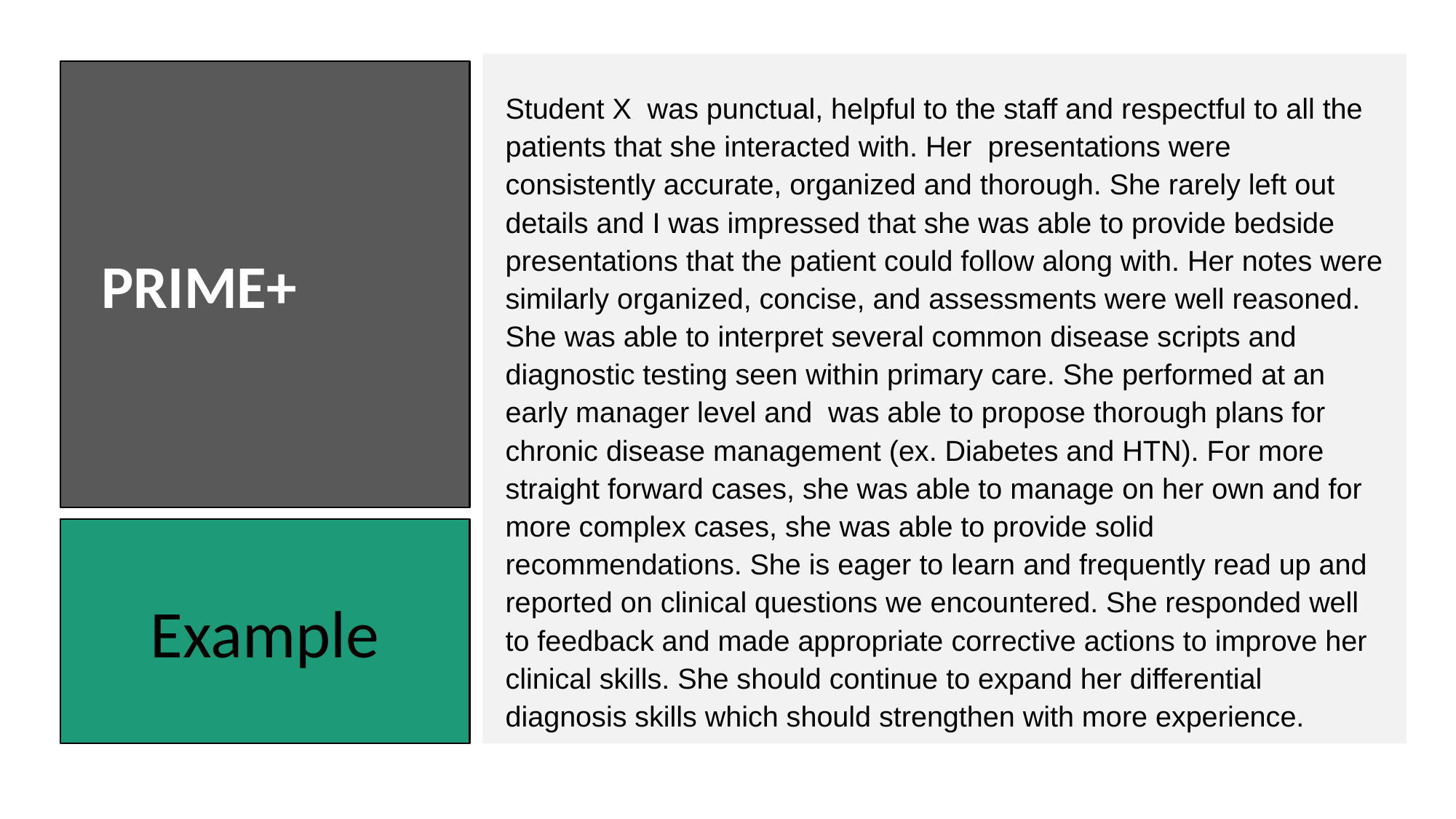

Student X was punctual, helpful to the staff and respectful to all the patients that she interacted with. Her presentations were consistently accurate, organized and thorough. She rarely left out details and I was impressed that she was able to provide bedside presentations that the patient could follow along with. Her notes were similarly organized, concise, and assessments were well reasoned. She was able to interpret several common disease scripts and diagnostic testing seen within primary care. She performed at an early manager level and was able to propose thorough plans for chronic disease management (ex. Diabetes and HTN). For more straight forward cases, she was able to manage on her own and for more complex cases, she was able to provide solid recommendations. She is eager to learn and frequently read up and reported on clinical questions we encountered. She responded well to feedback and made appropriate corrective actions to improve her clinical skills. She should continue to expand her differential diagnosis skills which should strengthen with more experience.
PRIME+
Example

## Slide 42
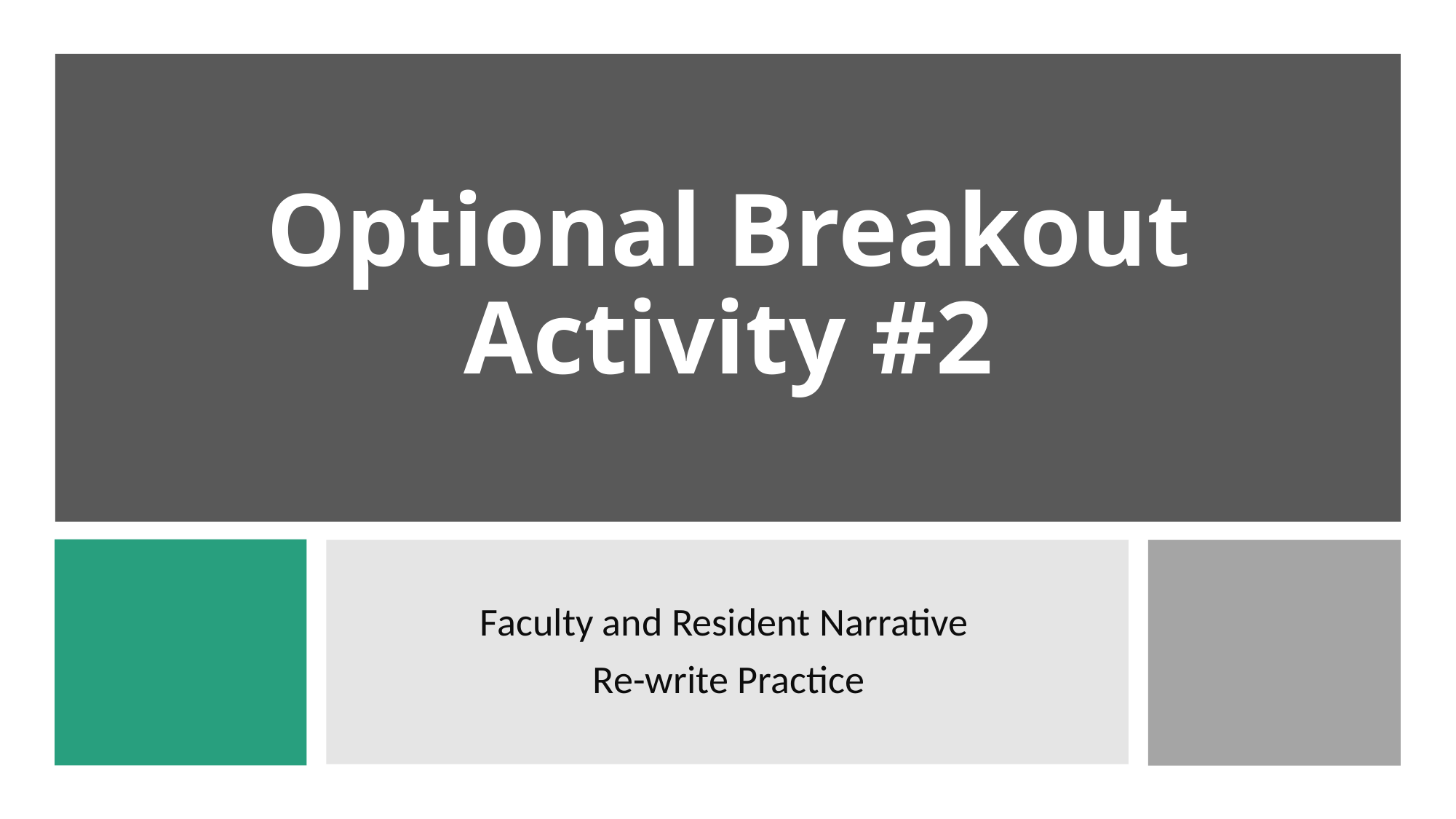

# Optional Breakout Activity #2
Faculty and Resident Narrative
Re-write Practice

## Slide 43
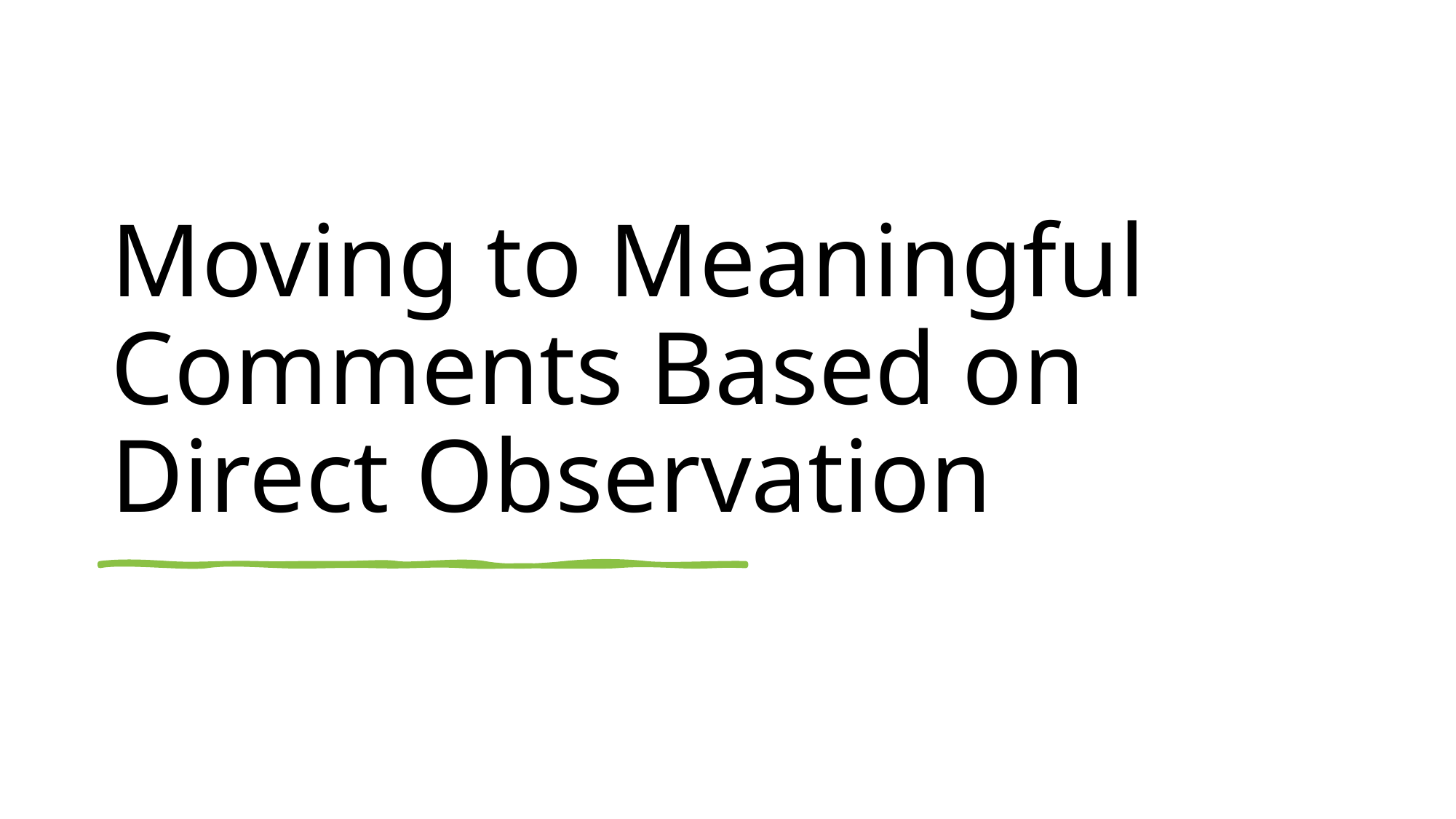

# Moving to Meaningful Comments Based on Direct Observation

## Slide 44
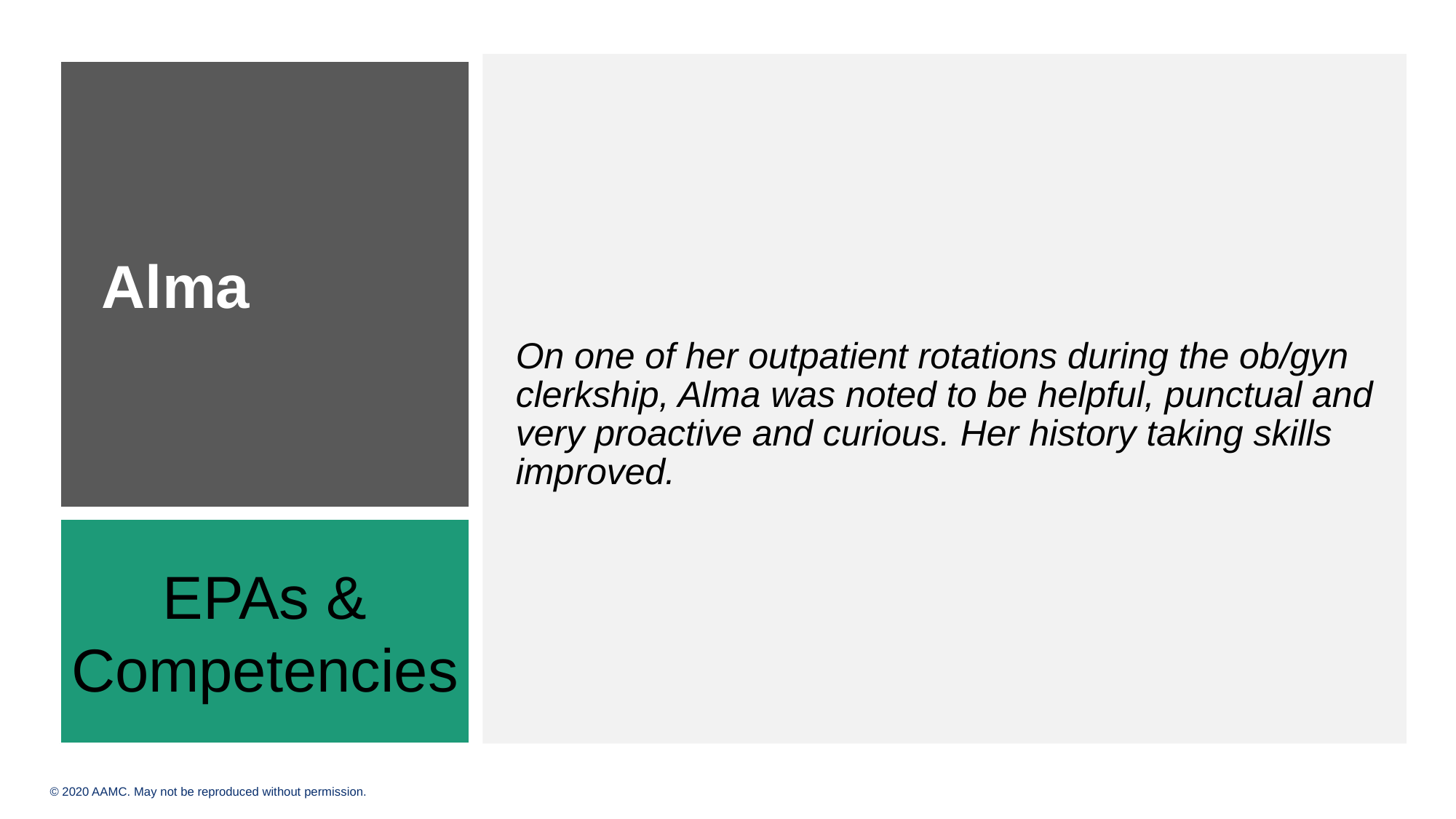

On one of her outpatient rotations during the ob/gyn clerkship, Alma was noted to be helpful, punctual and very proactive and curious. Her history taking skills improved.
Alma
# PRIME +
EPAs & Competencies
Example

## Slide 45
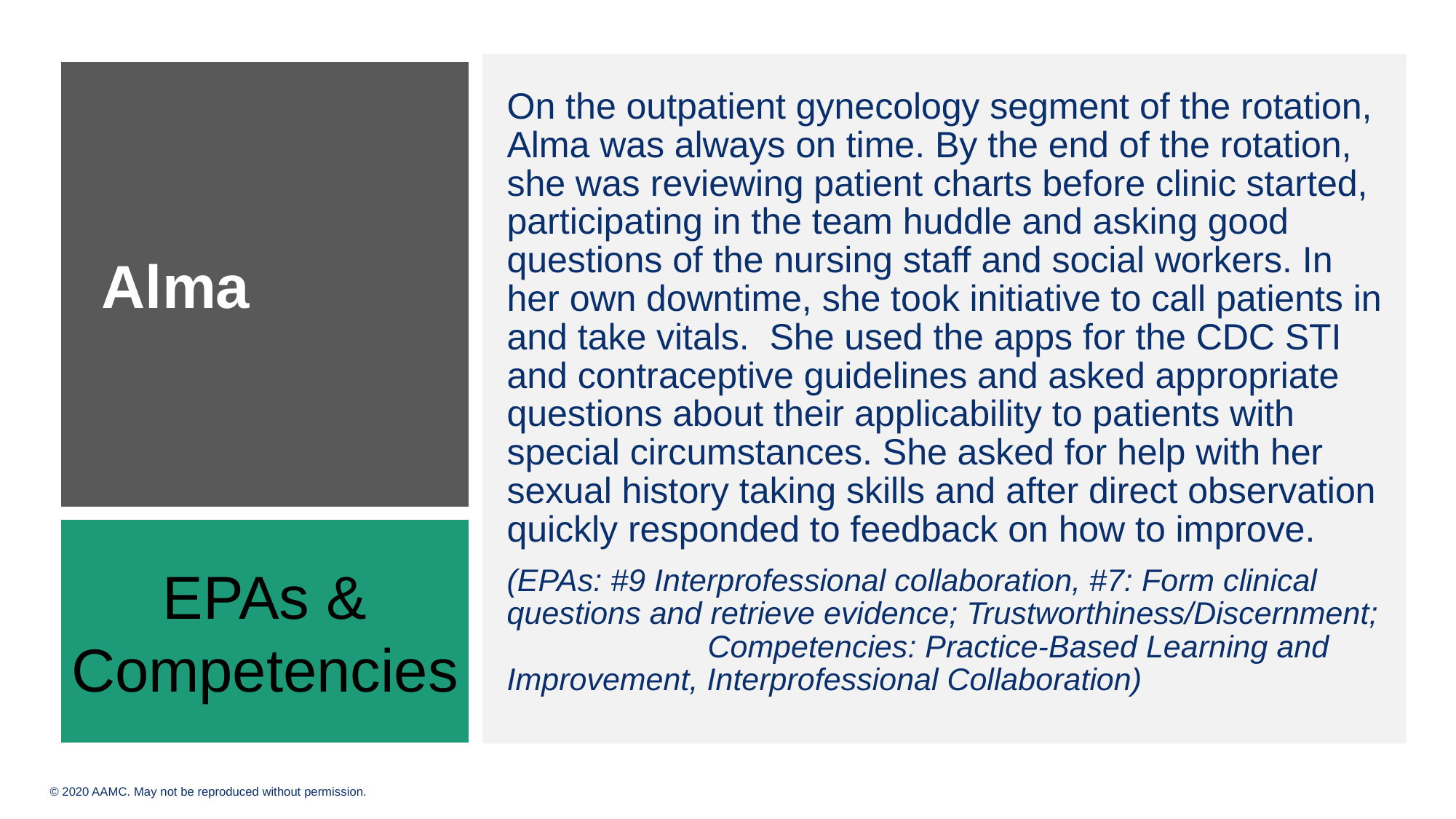

On the outpatient gynecology segment of the rotation, Alma was always on time. By the end of the rotation, she was reviewing patient charts before clinic started, participating in the team huddle and asking good questions of the nursing staff and social workers. In her own downtime, she took initiative to call patients in and take vitals. She used the apps for the CDC STI and contraceptive guidelines and asked appropriate questions about their applicability to patients with special circumstances. She asked for help with her sexual history taking skills and after direct observation quickly responded to feedback on how to improve.
(EPAs: #9 Interprofessional collaboration, #7: Form clinical questions and retrieve evidence; Trustworthiness/Discernment; Competencies: Practice-Based Learning and Improvement, Interprofessional Collaboration)
Alma
# PRIME +
EPAs & Competencies
Example

## Slide 46
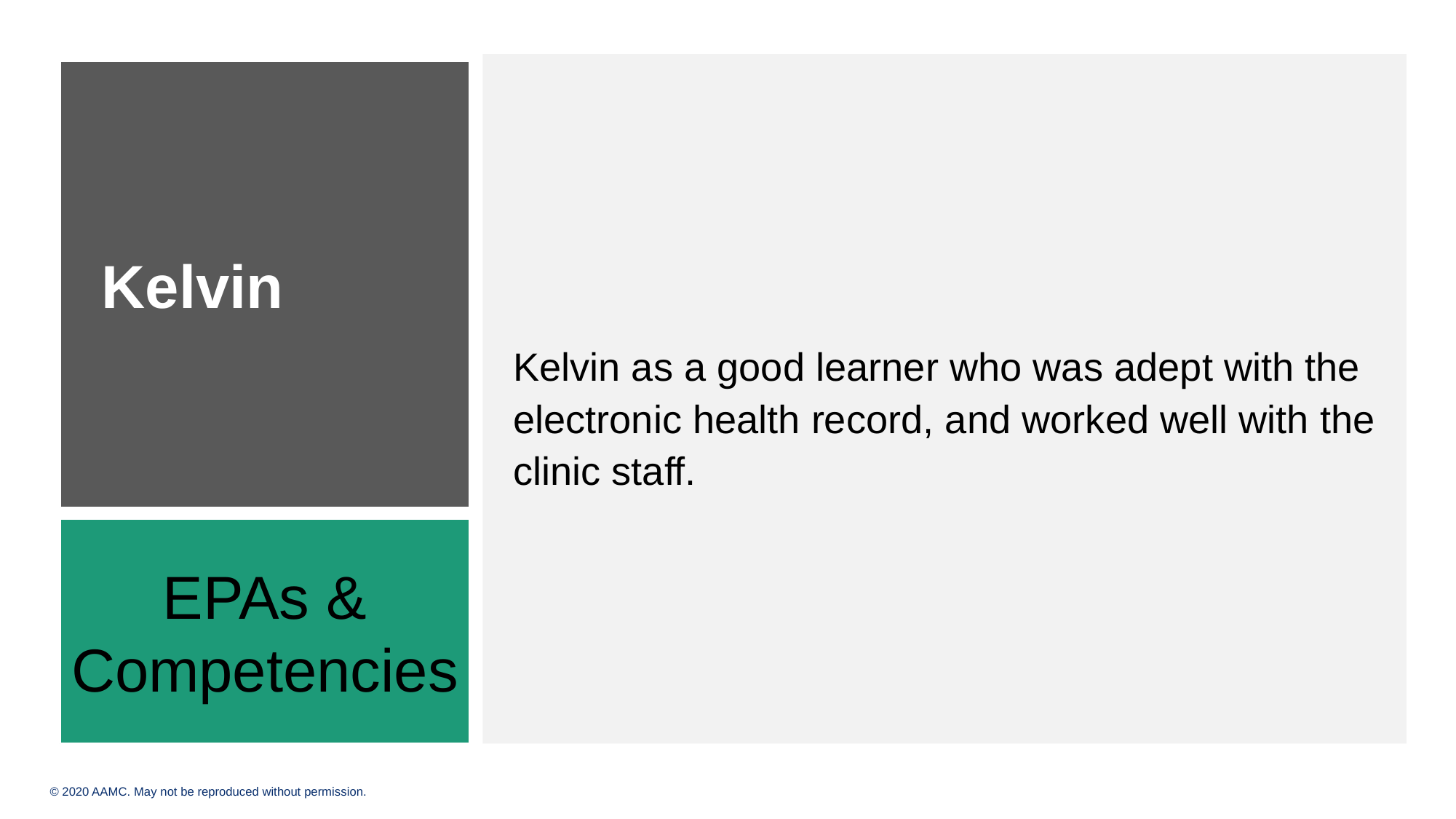

Kelvin as a good learner who was adept with the electronic health record, and worked well with the clinic staff.
Kelvin
# PRIME +
EPAs & Competencies
Example

## Slide 47
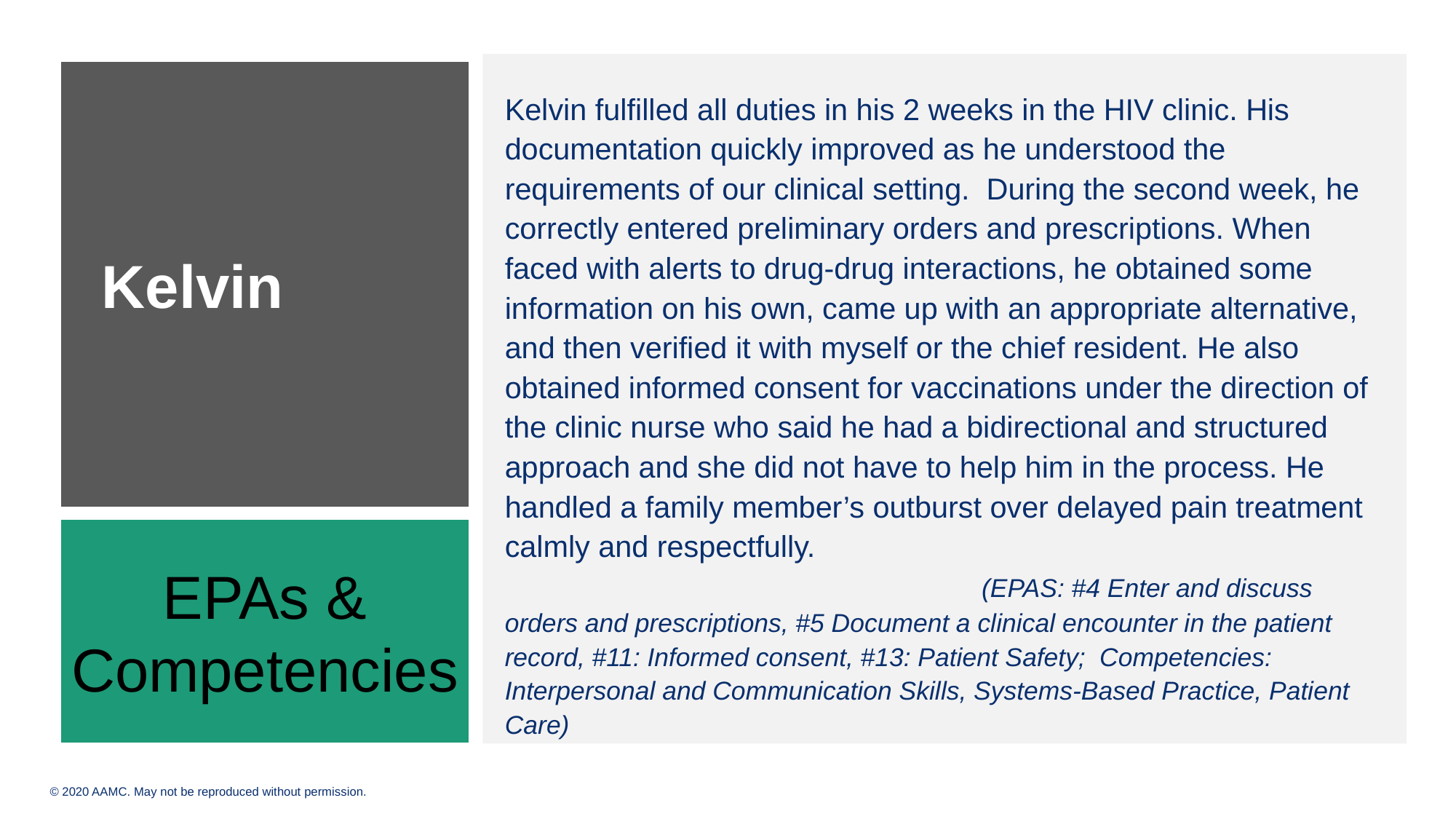

Kelvin fulfilled all duties in his 2 weeks in the HIV clinic. His documentation quickly improved as he understood the requirements of our clinical setting. During the second week, he correctly entered preliminary orders and prescriptions. When faced with alerts to drug-drug interactions, he obtained some information on his own, came up with an appropriate alternative, and then verified it with myself or the chief resident. He also obtained informed consent for vaccinations under the direction of the clinic nurse who said he had a bidirectional and structured approach and she did not have to help him in the process. He handled a family member’s outburst over delayed pain treatment calmly and respectfully. (EPAS: #4 Enter and discuss orders and prescriptions, #5 Document a clinical encounter in the patient record, #11: Informed consent, #13: Patient Safety; Competencies: Interpersonal and Communication Skills, Systems-Based Practice, Patient Care)
Kelvin
# PRIME +
EPAs & Competencies
Example

## Slide 48
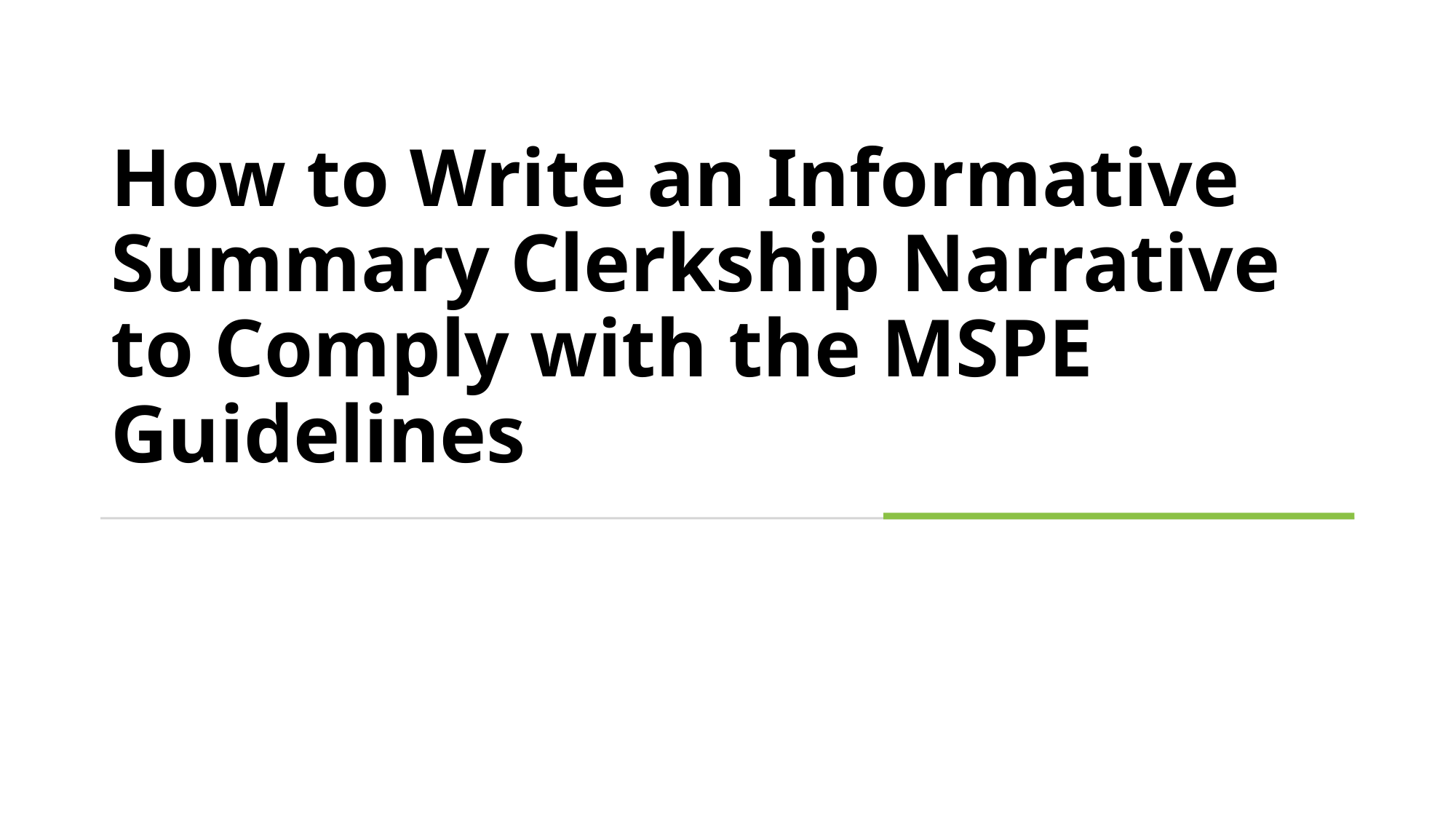

# How to Write an Informative Summary Clerkship Narrative to Comply with the MSPE Guidelines

## Slide 49
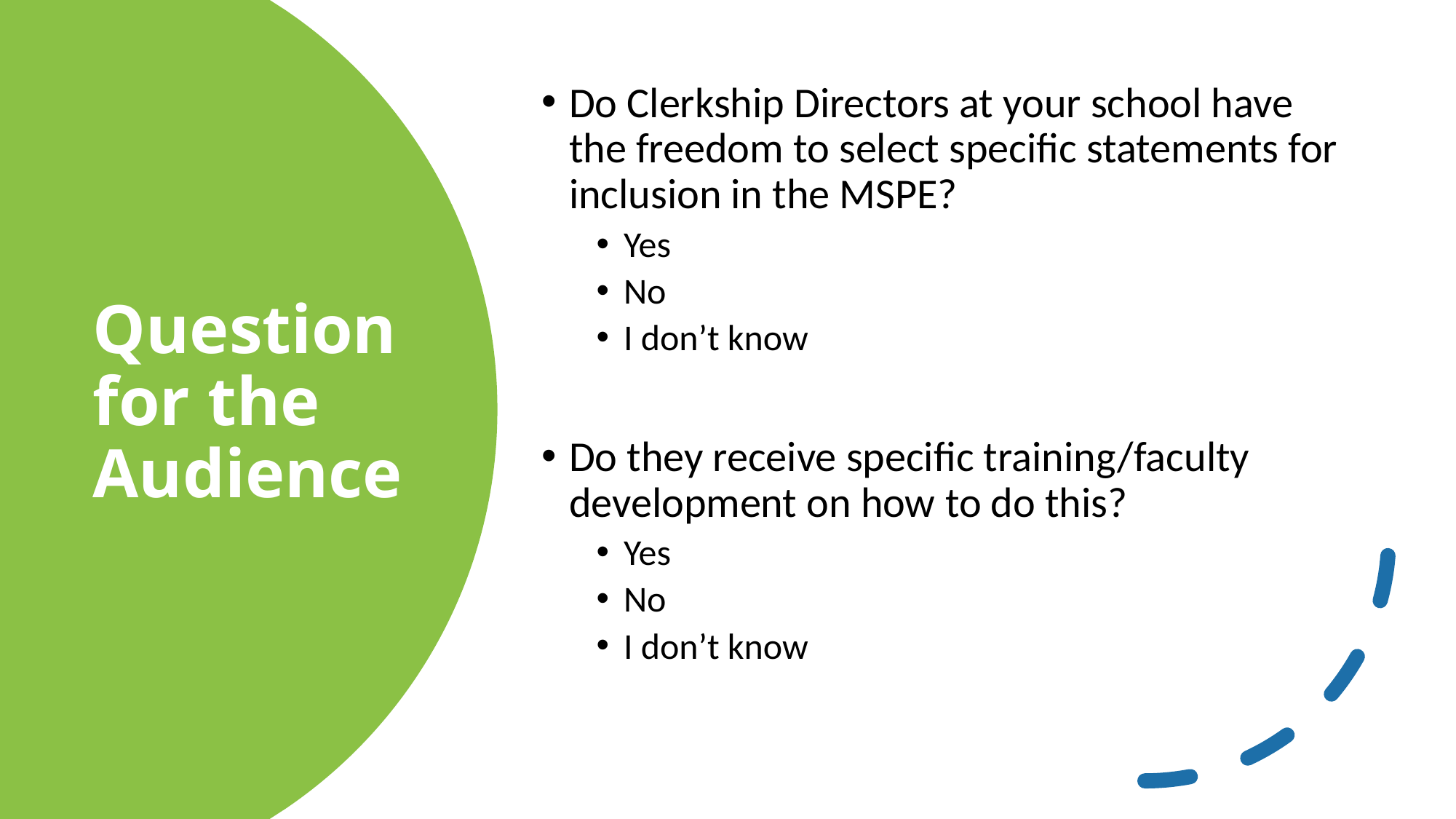

Do Clerkship Directors at your school have the freedom to select specific statements for inclusion in the MSPE?
Yes
No
I don’t know
Do they receive specific training/faculty development on how to do this?
Yes
No
I don’t know
# Question for the Audience

## Slide 50
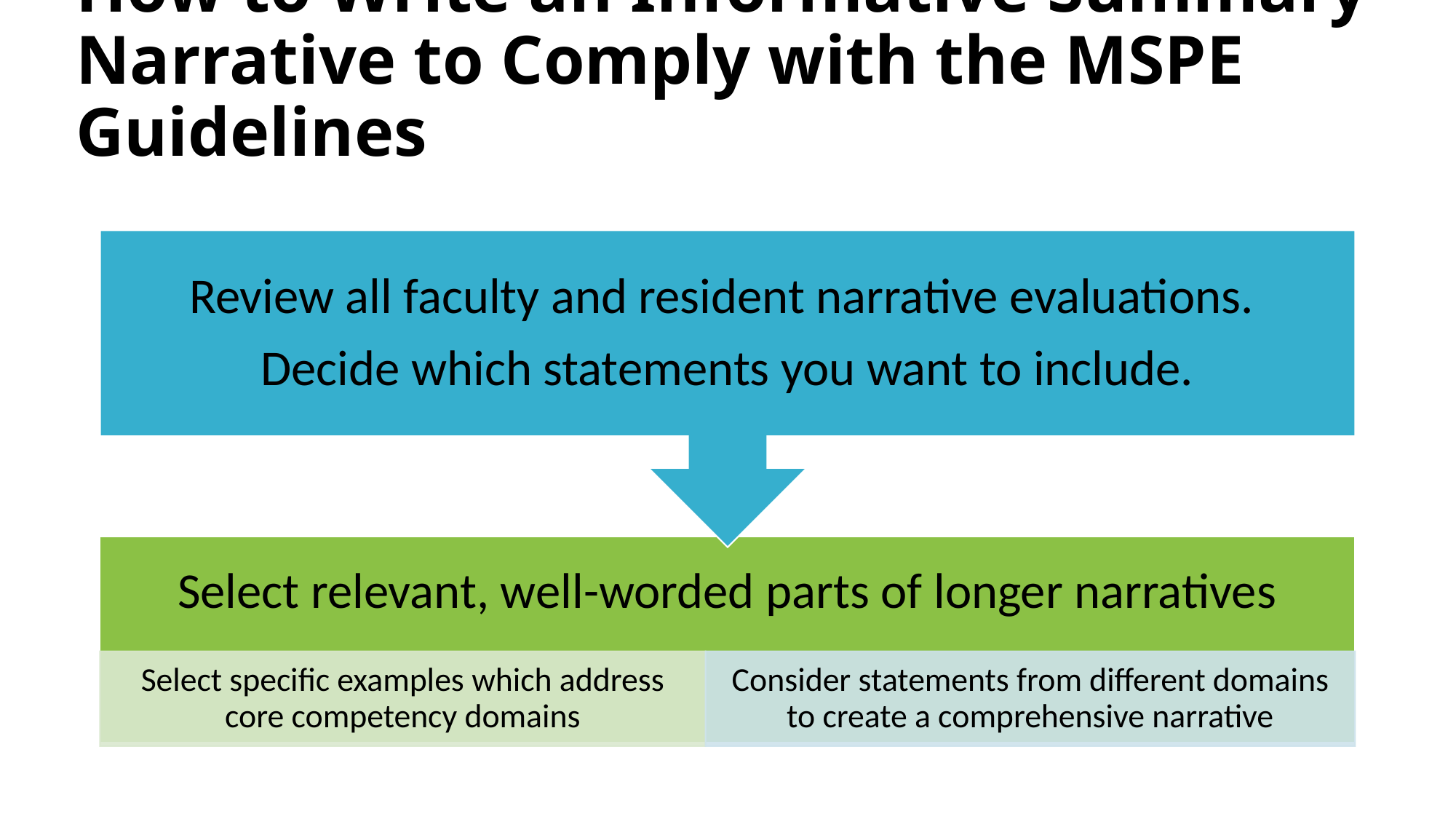

# How to Write an Informative Summary Narrative to Comply with the MSPE Guidelines

## Slide 51
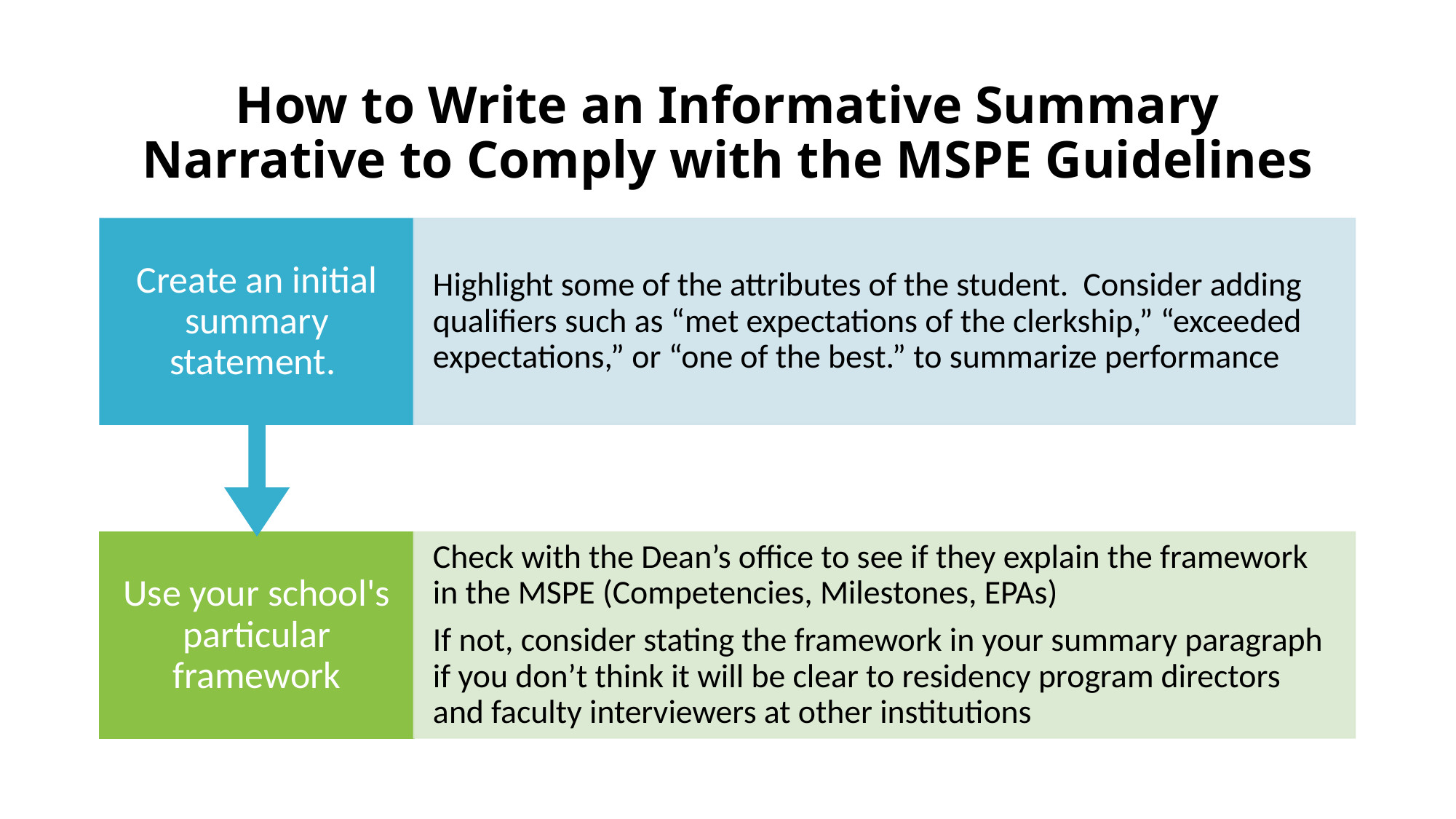

# How to Write an Informative Summary Narrative to Comply with the MSPE Guidelines

## Slide 52
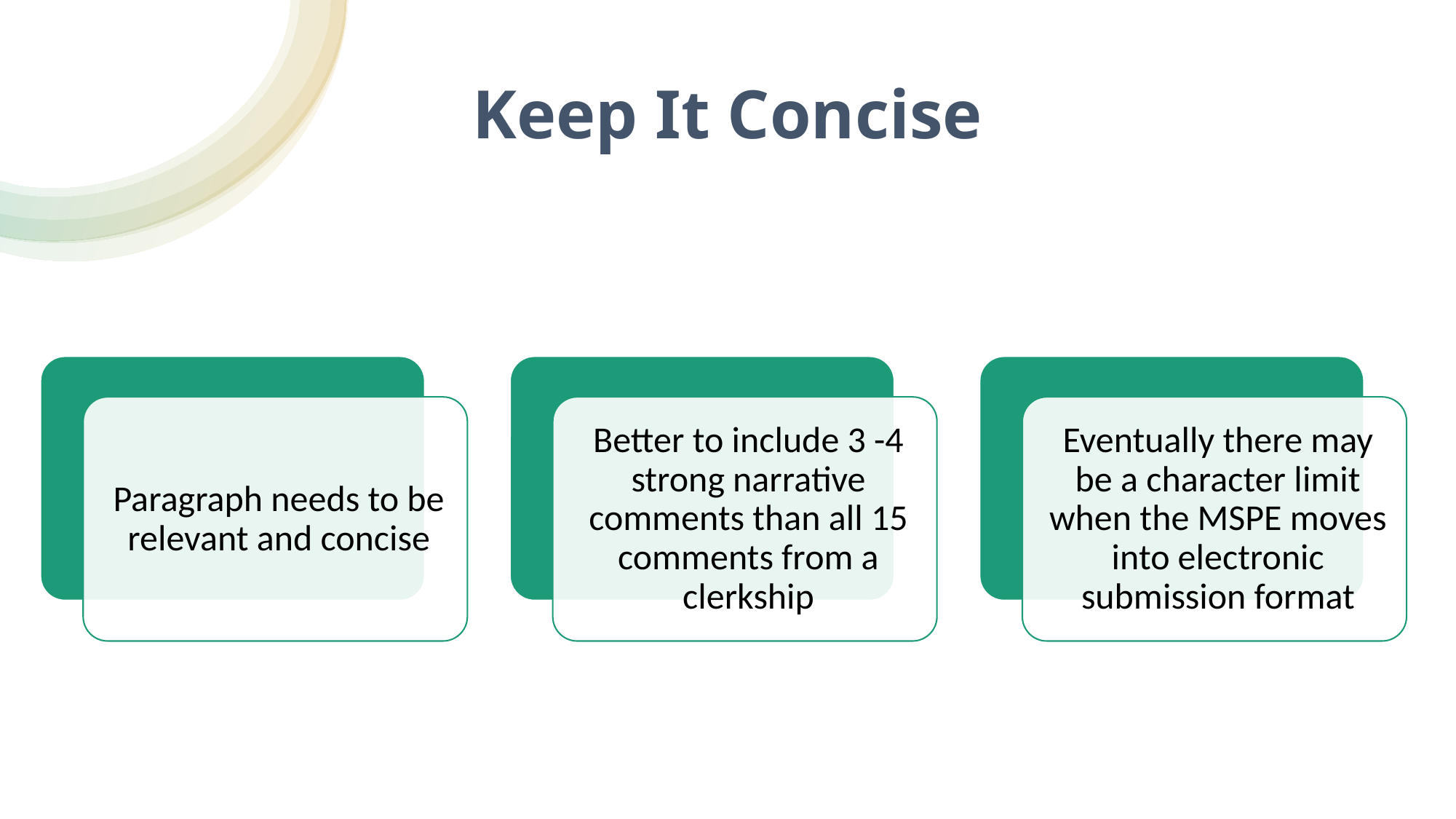

# Keep It Concise

## Slide 53
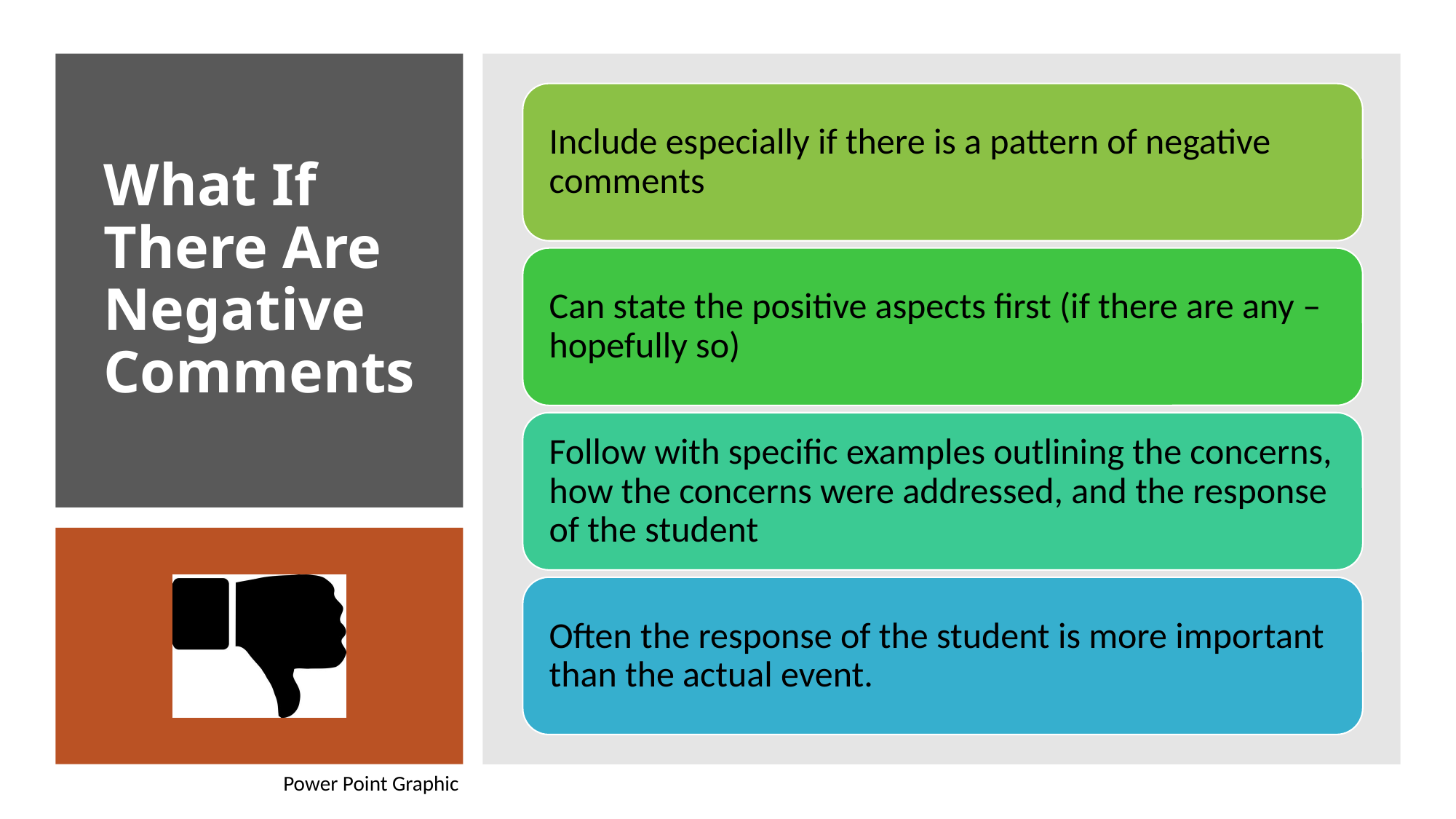

# What If There Are Negative Comments
Power Point Graphic

## Slide 54
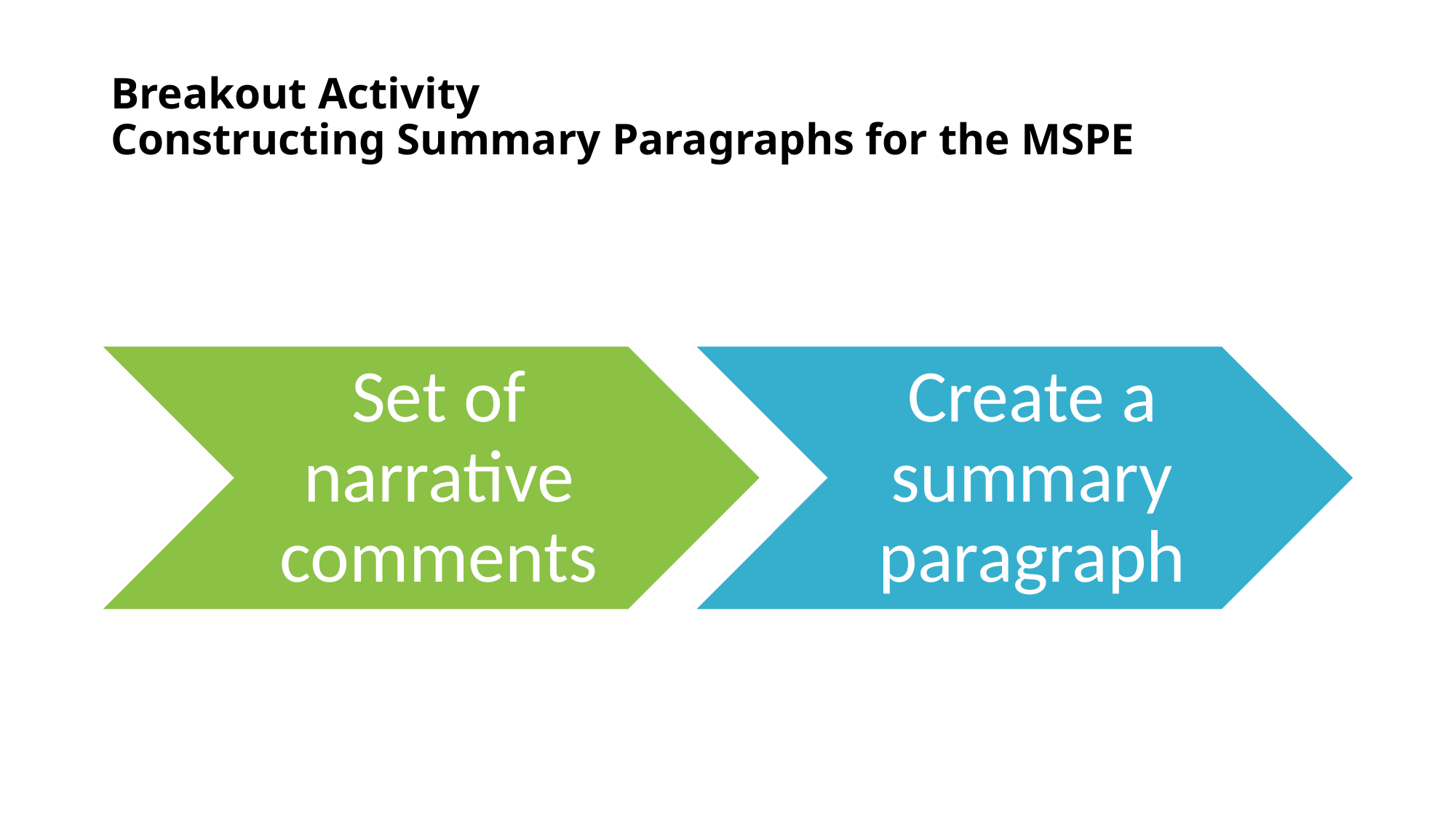

# Breakout Activity Constructing Summary Paragraphs for the MSPE

## Slide 55
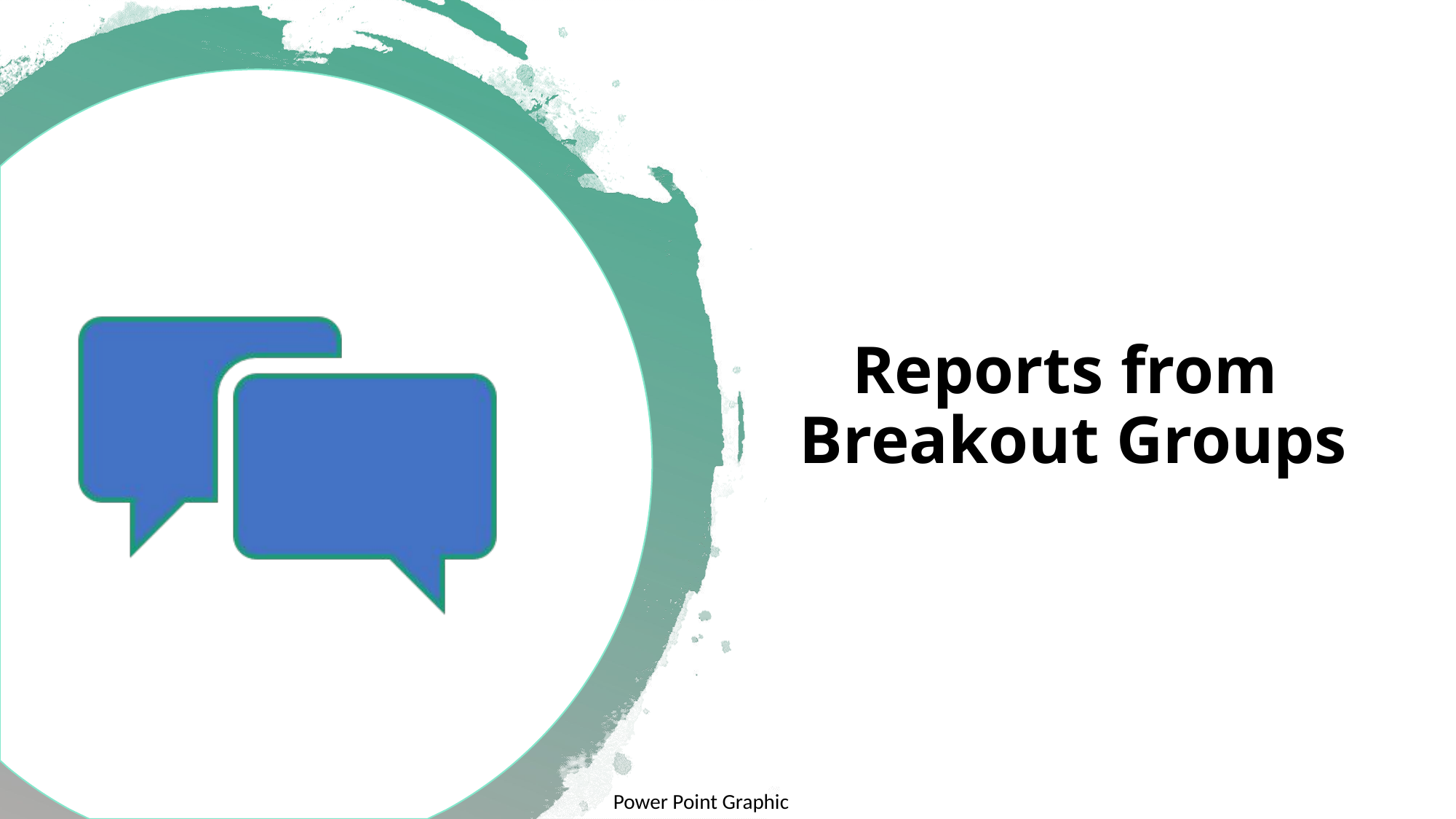

# Reports from Breakout Groups
Power Point Graphic

## Slide 56
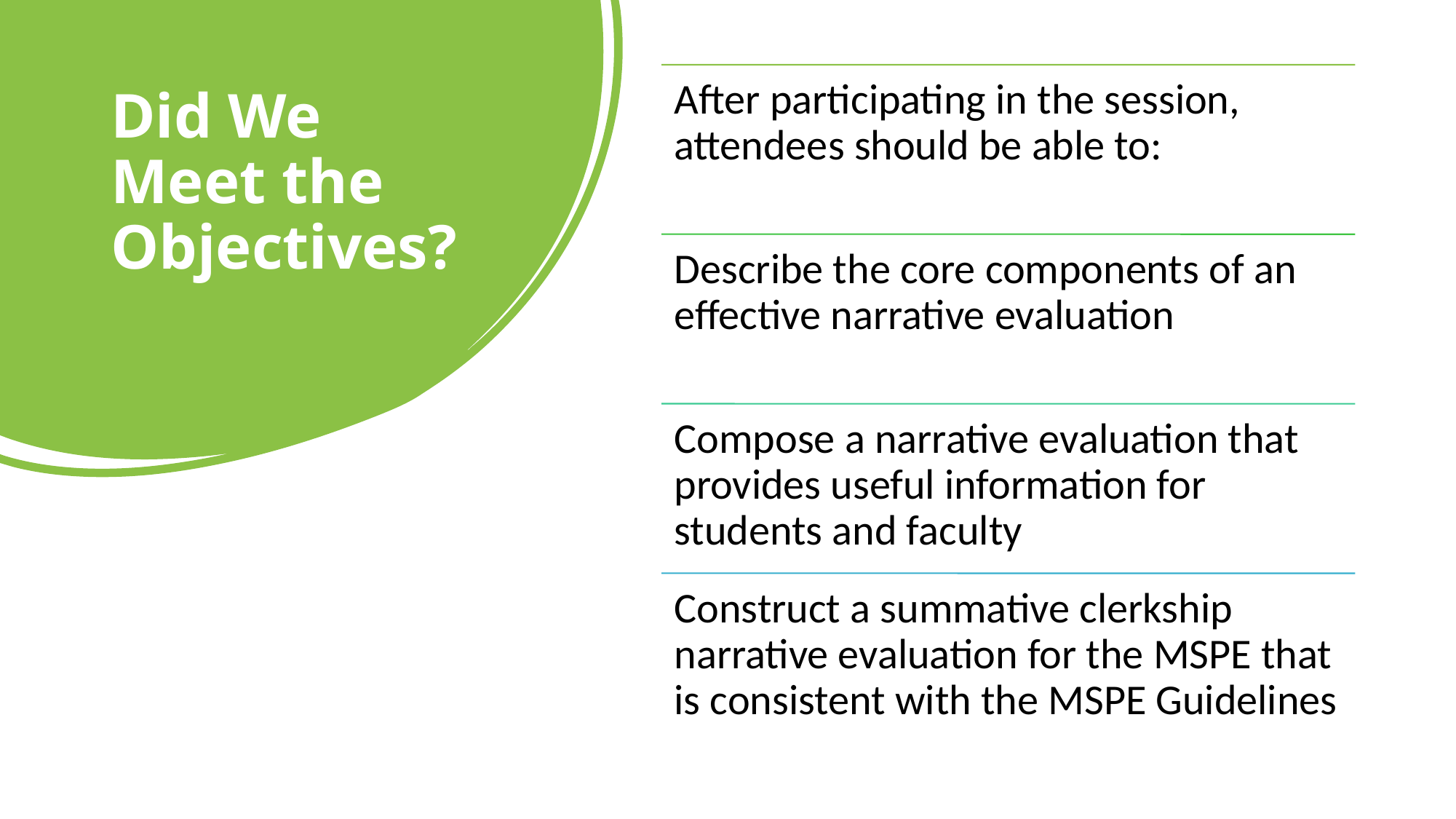

# Did We Meet the Objectives?

## Slide 57
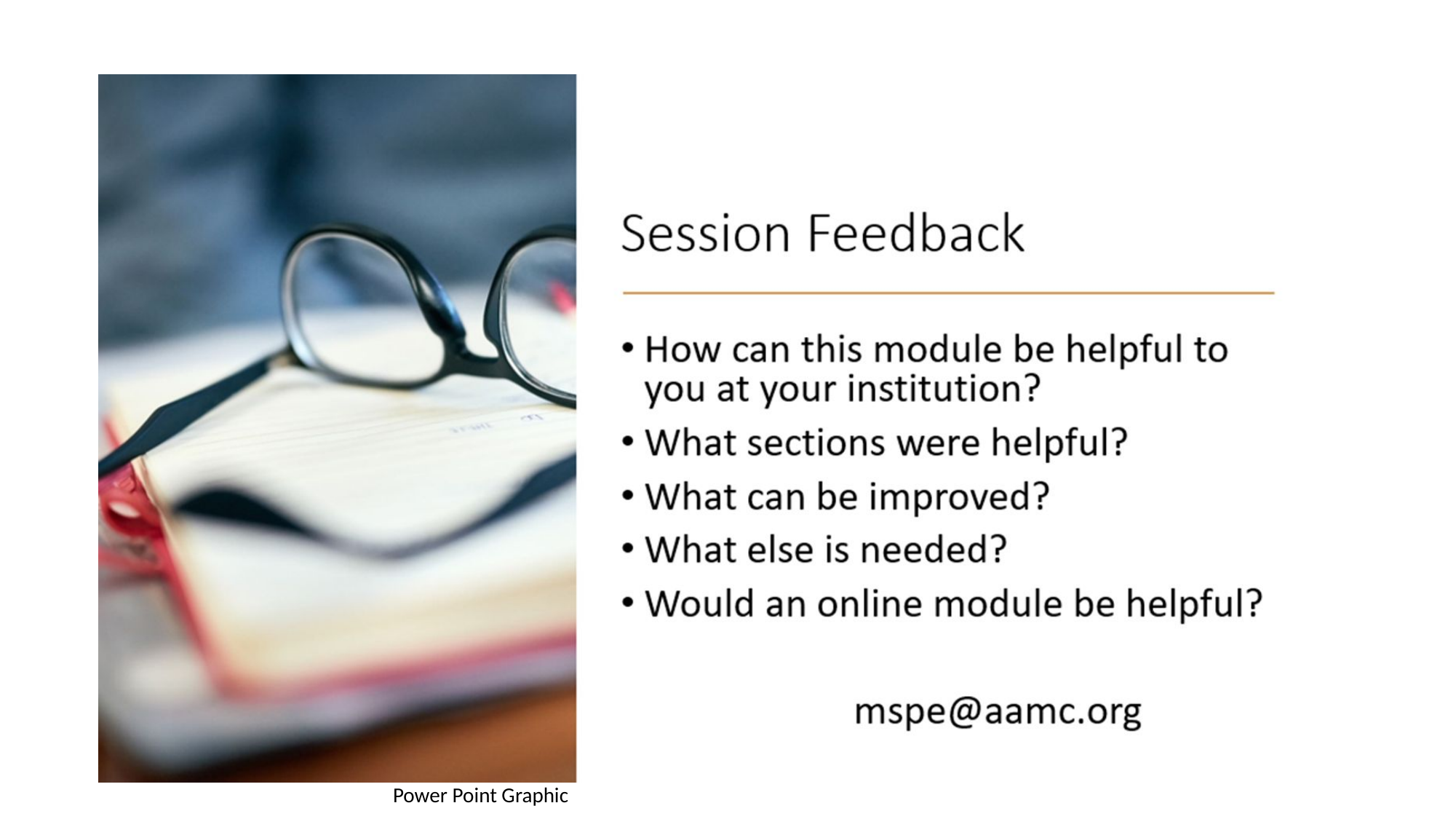

#
Power Point Graphic
