# Supplementary material for: Preparing Effective Narrative Evaluations for the Medical School Performance Evaluation (MSPE)
Source: MedEdPORTAL. 2022 Oct 4;18:11277. doi: 10.15766/mep_2374-8265.11277 (PMC9529862; doi:10.15766/mep_2374-8265.11277)
Supplement: Supplementary file 1 — Narrative Evaluations for the MSPE.pptxFacilitator Guide.docxActivity 1.docxActivity 2.docxActivity 2 Facilitator Guide.docxActivity 3.docxActivity 3 Facilitator Guide.docxEvaluation Form.docx [file mep_2374-8265.11277-s001.zip › B. Facilitator Guide.docx]

**Appendix B: Facilitator Guide**

**Workshop Title**

Preparing Effective Narrative Evaluations for the Medical School Performance Evaluation (MSPE)

**Background**

The American Association of Medical Colleges (AAMC) convened a working group in early 2019 to consider the current state of narrative feedback used to compose the Medical Student Performance Evaluation (MSPE). The intent of the working group was to build upon the work done by the MSPE Task Force that developed the 2016 MSPE Guidelines to enhance the transmission of useful information from UME to GME in the residency application process. This work contributes to move the focus from primarily quantitative measures to more qualitative measures of student performance and to find ways to explicate students’ competencies beyond medical knowledge to include patient care, professionalism, communication, practice-based learning and improvement, and systems-based practice. Additionally, with an improved quality of narrative comments programs will receive more accurate and consistent information about medical students.

**Purpose of Workshop**

This workshop is a faculty and resident development resource designed to result in higher quality narrative evaluations to be used as part of the MSPE. There is a specific section for clerkship directors or other individuals who assist in writing the final narrative paragraph included for each clerkship.

**Educational Objectives**

After participating in the session, attendees should be able to:

1. Describe the core components of an effective narrative evaluation
2. Compose a narrative evaluation that provides useful information for students and faculty, with attention to mitigating bias
3. Construct a summative narrative evaluation for the MSPE that is consistent with the AAMC MSPE Guidelines

**Conceptual Framework**

Deliberate Practice – The Teacher/Facilitator(s) provides immediate feedback during activities, allowing the learner to incorporate feedback to continuously improve their narrative evaluations

**Suggested Timeframe**

100 minutes is recommended to best achieve all educational objectives

A 90-minute format can also be utilized with brief shortening of the break-out activities

60 minutes is another option if clerkship director/summary clerkship narrative section is not covered or covered at a separate time

**Intended Audience(s)**

This workshop is intended for all levels of faculty or residents who complete narrative evaluations. Although these resources can be used in other healthcare fields, the materials are most relevant for those who evaluate medical students as part of their clinical rotations. The final section of this workshop specifically targets clerkship directors and others who combine narrative evaluations to create a narrative summary for inclusion in the MSPE.

**Prerequisites**

Workshop participants should have working familiarity with the ACGME (Accreditation Council for Graduate Medical Education) competencies and/or AAMC Physician Core Competency Reference Set, Entrustable Professional Activities (EPAs), and professionalism/reporter/interpreter/manager/educator (PRIME) frameworks. Facilitators should also have experience with documenting direct observation of clinical performance and experience composing narrative summaries for the MSPE.

**Teacher/Facilitator(s) Responsibilities**

The main facilitator should familiarize themselves with the slide content and notes, as well as various options for break-out activities. Activities should be selected that match the allotted timeframe for the activity. Facilitators should be familiar with the corresponding facilitator guides for each selected break-out activity. Having enough facilitators for each small group is ideal, in order to enhance direct feedback to participants.

**Required Resources**

This workshop can be conducted virtually or in person.

Virtually: Main teacher/facilitator should have easy access to the power point presentation (Appendix A) and all supplemental break-out activities for easy posting in the chat. Another individual should be available to assist with placement of participants in break-out rooms for the activities.

In person: Main teacher/facilitator should display the power point presentation using a laptop/computer and screen. Break-out activity worksheets can be provided to participants for use on laptops or printed. The facilitator guides for the break-out activities include an “answer key” for the various activities so facilitators should have these available for display on the screen or printed to share with participants. If using printed materials, green and yellow highlighters and pens should be provided.

**Implementation:**

This module can be used in its entirety or any of the sections or activities can be used independently or in various combinations to meet the needs of a faculty development session.

Detailed information for each slide is provided in the Notes section of the slide deck (Appendix A).

A suggested timeframe using the slides is included below.

| Activity | Materials | Duration | Time Elapsed |
| --- | --- | --- | --- |
| Background Presentation | Slides 1-5 (Appendix A) | 5 minutes | 5 minutes |
| Program Directors’ Perspective | Slides 6-14 (Appendix A) | 10 minutes | 15 minutes |
| Pitfalls of Narratives Related to Bias | Slides 15-18 (Appendix A) | 3 minutes | 18 minutes |
| Overcoming the Challenges of Writing Effective Narratives | Slides 19-27 (Appendix A) | 10 minutes | 28 minutes |
| Optional Poll Questions | Slides 28-29 (Appendix A) | 5 minutes | 33 minutes |
| Activity #1: Competency-based and PRIME frameworks | Slides 30-41  (Appendices A and C) | 15-20 minutes  (depending on activity selected) | 50 minutes  (48 – 53 minutes) |
| Activity #2: Optional Break-Out  Faculty and Resident  Narrative Re-write Practice | Slide 42  (Appendices A, D and E) | 15 minutes | 65 minutes |
| Moving to Meaningful Narratives Based on Direct Observation | Slides 43 – 47  (Appendix A) | 5 minutes | 70 minutes |
| How to Write an Informative Summary Narrative | Slides 48 – 53  (Appendix A) | 10 minutes | 80 minutes |
| Activity #3: Optional Break-Out Activity  Summary Clerkship Paragraphs | Slides 54-55 (Appendices A, F, and G) | 15 minutes | 95 minutes |
| Session Feedback/Workshop Evaluation | Slide 56-57  (Appendix H) | 5 minutes | 100 minutes |

**Breakout Activity #1** can be completed using the slide set (Slides 32-41) for larger group and inviting audience participation or using the worksheet (Appendix C) in smaller breakout groups of 2 to 4 individuals. **S**ome or all examples may be used as allowed by session purpose, format, and timing.

**Breakout Activity #2** (Appendix D) is best completed in break-out groups of 4 to 6 individuals with facilitators available for each group in order to provide suggestions/feedback on the narratives that the group creates. A facilitator guide is available (Appendix E) for this activity.

**Breakout Activity #3** (Appendix F) should ideally be done in groups of 4 to 6. Groups can be assigned to complete both cases on the worksheet or some groups can be assigned to the first one and others to the second. Based on feedback from other workshops, participants prefer to work on both cases since the first one is more straightforward and allows practice prior to the second one which has negative comments for a student who underwent remediation. The Facilitator’s Guide (Appendix G) will allow the facilitator for each group to provide direct feedback on the drafted summary paragraph from the group and make additional suggestions.

**Assessment of Learning Objectives:**

Educational Objective 1 “Describe the core components of an effective narrative evaluation” can be evaluated in Breakout Activity #1 and #2 by the facilitators observing the participants naming and utilization of these core components presented in evaluating and constructing narratives. In addition, participants can be asked about their ability to describe these core components.

Educational Objective 2 “Compose a narrative evaluation that provides useful information for students and faculty, with attention to mitigating bias” can be directly observed by the facilitators during breakout activities, most specifically Activity #2. In addition, participants can be directly asked about their achievement of this objective.

Educational Objective 3 “Construct a summative narrative evaluation for the MSPE that is consistent with the AAMC MSPE Guidelines” is best evaluated during Activity #3 through direct observation by facilitators. In addition, participants can be directly asked about their achievement of this objective.

**Workshop Evaluation:**

Appendix H provides an Evaluation Form that can be used in the delivery of this workshop and specifically asks about activity as a worthwhile investment in their professional developed, acquisition of new knowledge and skills, application of knowledge and skills and relevance to professional role.

**Extension Activities:**

Faculty and residents can be asked to bring some of their own narratives to this workshop in order to evaluate and create re-writes using the core components and feedback provided. In addition, it would be very interesting to look at narratives pre and post workshop to determine improvements made.

**References:**

1. Association of American Medical Colleges. Recommendations for Revising the Medical Student Performance Evaluation (MSPE). <https://www.aamc>. org/system/files/c/2/470400-mspe-recommendations. pdf. Published 2017. Accessed November 10 2021.
2. National Resident Matching Program, Data Release and Research Committee: Results of the 2020 NRMP Program Director Survey <https://www.nrmp.org/wp-content/uploads/2020/08/2020-PD-Survey.pdf>.  Accessed November 10, 2021.
3. Singer C, Ficalora R, Addams A. Creating a mutually valuable MSPE. Presented at AAMC Continuum Connections: A Joint Meeting of the Group on Student Affairs, Group on Resident Affairs, and the Organization of Student Representatives, and Organization of Resident Representatives. April 30, 2021; Orlando, Florida.
4. Brenner JM, Arayssi T, Conigliaro RL, and Friedman K. (*2019*) The Revised Medical School Performance Evaluation: Does it meet the needs of its readers? *J Grad Med Educ.*2019;11(4):475-478.
5. Saudek K, Treat R, Goldblatt M, et al. Pediatric, surgery, and internal medicine program director interpretations of letters of recommendation. *Acad Med.*2019;94(11):S64-S68.
6. Ross DA, Boatright D, Nunez-Smith M, et al. Differences in words used to describe racial and gender groups in Medical Student Performance Evaluations. *PLoS One*. 2017;12(8):e0181659. Published 2017 Aug 9. doi:10.1371/journal.pone.0181659.
7. Zhang N, Blissett S, Anderson D, et al. Race and gender bias in intern medicine program director letters of recommendation. *J Grad Med Educ.*Published online 2021. <https://doi.org/10.4300/JGME-D-20-00929.1>
8. Ginsburg, S., van der Vleuten, C., Eva, K. W., & Lingard, L. (2016). Hedging to save face: a linguistic analysis of written comments on in-training evaluation reports. Adv Health Sci Educ Theory Pract, 21(1), 175-188.
9. Hanson JL, Rosenberg AA, Lane JL. (2013) Narrative descriptions should replace grades and numerical ratings for clinical performance in medical education in the United States. Frontiers in Psychology.
10. Lye, P. S., Biernat, K. A., Bragg, D. S., & Simpson, D. E. (2001). A pleasure to work with - An analysis of written comments on student evaluations. Ambulatory Pediatrics, 1(3), 128-131.
11. Rojek A, Khanna R, Yim, JWL et al Differences in Narrative Language in Evaluations of Medical Students by Gender and Under-represented Minority Status J Gen Intern Med 2019 May;34(5):684-691.
12. Teherani A, Hauer KE, Fernandez A, How Small Differences in Assessed clinical Performance amplify to Large Differences in Grades and Awards: A cascade With Serious Consequences for Students Underrepresented in Medicine. Acad Med 2018 Sep;93(9):1286-1292
13. Bird JB, Friedman KA, Arayssi T, et al.  Review of the Medical Student Performance Evaluation: analysis of the end users’ perspective across the specialties. Medical Education Online 2021, Vol. 26, 1876315
14. Holmes AV, Peltier CB, Hanson JL, et al. Writing Medical Student and Resident Performance Evaluations: Beyond “Performed as Expected”.  Pediatrics Volume 2014 May; 133(5):766-768.
15. Chan A, Cao A, Kim L et al. Comparison of perceived educational value of an in-person versus virtual medical conference. [Can Med Educ J.](https://www.ncbi.nlm.nih.gov/pmc/articles/PMC8463218/) 2021 Sep; 12(4): 65–69.
