# Supplementary material for: Preparing Effective Narrative Evaluations for the Medical School Performance Evaluation (MSPE)
Source: MedEdPORTAL. 2022 Oct 4;18:11277. doi: 10.15766/mep_2374-8265.11277 (PMC9529862; doi:10.15766/mep_2374-8265.11277)
Supplement: Supplementary file 1 — Narrative Evaluations for the MSPE.pptxFacilitator Guide.docxActivity 1.docxActivity 2.docxActivity 2 Facilitator Guide.docxActivity 3.docxActivity 3 Facilitator Guide.docxEvaluation Form.docx [file mep_2374-8265.11277-s001.zip › C. Activity 1.docx]

**Appendix C – Optional Activity #1: Competency-Based Approach to Clinical Narratives (Can be used with Appendix A slides)**

**Read over the assigned cases and identify the relevant ACGME competencies, Entrustable Professional Activities (EPAs), Knowledge/Skills/Attitude (KSA) or PRIME+ elements**

**ACGME Competencies: Patient Care, Medical Knowledge, Practice-Based Learning and Improvement, Interpersonal and Communication Skills, Professionalism and Ethics, System-Based Practice**

**EPAS: Observable activities that allow preceptors to assess learners on various competencies**

**PRIME:**

**P**- Responsibility, attire, attitude, response to feedback, motivation

**R** – Gather the appropriate, complete information

**I** – Prioritize, critical thinking, differential diagnosis

**M**- Discuss the most logical next step

**E** – Self-directed learner, ability to educate others

**+** - What was done well and modify, what did not do well?

| **Case #1**  **Slide  #32** | “Student A was always prepared, having studied patient charts in depth prior to entering the exam room. In one instance, her preparation saved potential disaster. When a patient failed to show for her appointment, Student A realized the patient had not been given a referral for the evaluation of a mass in her thyroid. Due to her excellent preparation and alarm, we were able to intervene” | **Competencies** |
| --- | --- | --- |
|  |  | **EPAs** |
|  |  | **Other (PRIME/KSA)** |
| **Case #2**  **Slide #33** | "Patient  presentations and assessments were thorough, polished and organized. If I interrupted a presentation to ask a question, he got right back into the flow w/ ease and  incorporated feedback immediately,  For example, with a headache patient- he was able to Dx migraine w/ aura instead of complicated migraine, immediately internalizing the feedback I had given at the start of his presentation. Given several topics to discuss; always came prepared with well thought out and put together presentations. Exceptional team player, unmatched work ethic. Superior pt notes including some that were TRULY outstanding, including sophisticated localization. He created a Neurocritical care template-note for future students to use." | **Competencies** |
|  |  | **EPAs** |
|  |  | **Other (PRIME/KSA)** |
| **Case #3**  **Slide #34** | “Nancy was a valuable member of our team and was attentive on rounds, at times knowing details about the patients and their management plans that the interns and senior resident could not remember. She also went beyond the duties of a 3rd year student and went out of her way to assist interns with tasks such as tracking down a family member for a patient with dementia who needed a procedure and could not provide consent.” | **Competencies** |
|  |  | **EPAs** |
|  |  | **Other (PRIME/KSA)** |
| **Case #4**  **Slide #35** | “Student K is an exceptional individual and an outstanding student. Driven by a deep sense of compassion and justice, and recognizing inequalities in health care delivery, she decided long ago that she would become a physician. This passion was apparent every day during her elective, as she sought to understand patient needs beyond their physical ailments. Her patient interviews always included questions regarding living conditions, employment, and other social determinants so that she could get a complete picture of any obstacles to care.” | **Competencies** |
|  |  | **EPAs** |
|  |  | **Other (PRIME/KSA)** |
| **Case #5**  **Slide #36** | “Student B possess a superior knowledge base of physiology and basic science, above that seen in most MS3 students. | **Competencies** |
|  |  | **EPAs** |
|  |  | **Other (PRIME/KSA)** |
| **Case #6**  **Slide #37** | “Student B demonstrated an ability to apply his deep understanding of physiology and basic science to new clinical problems. This was demonstrated by his ability formulate appropriate differential diagnoses and propose sensible steps to narrowing the diagnosis and developing a therapy plan. For one patient he discussed drug adverse effects, that I had not considered in the differential.” | **Competencies** |
|  |  | **EPAs** |
|  |  | **Other (PRIME/KSA)** |
| **Case #7**  **Slide #38** | Maria did a good job presenting complicated patients on rounds, able to synthesize large patient data and generate a good differential diagnosis and management plan. | **Competencies** |
|  |  | **EPAs** |
|  |  | **Other (PRIME/KSA)** |
| **Case #8**  **Slide #39** | Maria did a good job presenting complicated patients on rounds, able to synthesize large patient data and generate a good differential diagnosis and management plan. She was very engaged and proactive, for example, doing orthostatics on her pts to assess volume status or doing MME to assess pts for underlying dementia. She also collected valuable patient data from doctor's offices that were a great help in assessing and managing patients. She had great rapport with patients and all team members caring for the patient. | **Competencies** |
|  |  | **EPAs** |
|  |  | **Other (PRIME/KSA)** |
| **Case #9**  **Slide #41** | Student X was punctual, helpful to the staff and respectful to all the patients that she interacted with. Her presentations were consistently accurate, organized and thorough. She rarely left out details and I was impressed that she was able to provide bedside presentations that the patient could follow along with. Her notes were similarly organized, concise, and assessments were well reasoned. She was able to interpret several common disease scripts and diagnostic testing seen within primary care. She performed at an early manager level and was able to propose thorough plans for chronic disease management (ex. Diabetes and HTN). For more straightforward cases, she was able to manage on her own and for more complex cases, she was able to provide solid recommendations. She is eager to learn and frequently read up and reported on clinical questions we encountered. She responded well to feedback and made appropriate corrective actions to improve her clinical skills. She should continue to expand her differential diagnosis skills which should strengthen with more experience. | **Competencies** |
|  |  | **EPAs** |
|  |  | **Other (PRIME/KSA)** |
