# Supplementary material for: Preparing Effective Narrative Evaluations for the Medical School Performance Evaluation (MSPE)
Source: MedEdPORTAL. 2022 Oct 4;18:11277. doi: 10.15766/mep_2374-8265.11277 (PMC9529862; doi:10.15766/mep_2374-8265.11277)

**Appendix D**

**Breakout Activity #2**

**Faculty and Resident Narrative Re-write Practice**

Utilizing the checklist below, evaluate the narrative and rewrite it to meet as many of the checklist elements as possible. Each breakout group should choose 2 narratives to evaluate and rewrite.

**Assessing the Critical Elements of High Quality Narratives**

- Provides *specific* comments
- Avoids bias
- Comments seem to be based on *direct observation* of learner by evaluator
- Describes areas of strength with clear examples
- Describes areas for growth/improvement
- Comments related to specific competencies (knowledge for practice, patient care, communication, professionalism, systems-based practice, practice-based learning and improvement, interprofessional collaboration)
- What would make this narrative stronger?
- What would help a program director get to know this student better?

Jessica: “Team player. Excellent knowledge base. Knew her patients well. Always willing to do extra work. Pleasure to have on the rotation. Should continue reading to learn more.”

Evaluate using checklist and then rewrite:

Stuart: “Very professional student. Impressive knowledge base and strong differential diagnoses. Efficient. Comes in early. Great oral presentations.”

Evaluate using the checklist and then rewrite:

Amy: “Amy has done an excellent job on this rotation. She is responsible, always on time, reads about the subjects assigned to her. She was prepared and read for each returning patient and knew guidelines very well. Helpful in clinic.”

Evaluate using checklist and then rewrite:


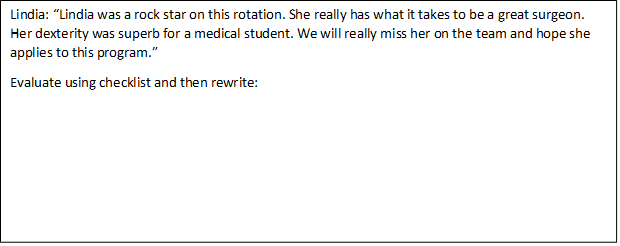

Supplement: Supplementary file 1 — Narrative Evaluations for the MSPE.pptxFacilitator Guide.docxActivity 1.docxActivity 2.docxActivity 2 Facilitator Guide.docxActivity 3.docxActivity 3 Facilitator Guide.docxEvaluation Form.docx [file mep_2374-8265.11277-s001.zip › D. Activity 2.docx]
