# Supplementary material for: Preparing Effective Narrative Evaluations for the Medical School Performance Evaluation (MSPE)
Source: MedEdPORTAL. 2022 Oct 4;18:11277. doi: 10.15766/mep_2374-8265.11277 (PMC9529862; doi:10.15766/mep_2374-8265.11277)
Supplement: Supplementary file 1 — Narrative Evaluations for the MSPE.pptxFacilitator Guide.docxActivity 1.docxActivity 2.docxActivity 2 Facilitator Guide.docxActivity 3.docxActivity 3 Facilitator Guide.docxEvaluation Form.docx [file mep_2374-8265.11277-s001.zip › E. Activity 2 Facilitator Guide.docx]

**Appendix E**

**Optional Breakout Activity #2 Facilitator’s Guide**

**Faculty and Resident Narrative Re-write Practice**

**Facilitator Instructions**

**In-Person:**

1) Prepare copies of Appendix D for participants

2) Have participants pair or work in groups to review provided narratives in Appendix D. Each pair/group should work on 2 of the scenarios. Suggested time 9 minutes

3) Participants should look at the checklist, consider what would make the narrative stronger, and think of other information that would help a program director get to know this student better

4) Participants can write out suggestions on the Appendix D copies

5) Facilitators can review suggested re-writes in large group format (6 minutes) or provide Appendix  E re-writes to the pairs/groups to review on their own. If facilitators are available for each small group, facilitators can share suggestions from Appendix E with the group.

**Virtual:**

1) Paste Appendix D in the chat

2) Utilize breakout rooms to review provided narratives in Appendix D. Each group should work on 2 of the scenarios.  Ideally, there would be a facilitator or lead in each of the breakout rooms.

3) Participants should look at the checklist, consider what would make the narrative stronger, and think of other information that would help a program director get to know this student better

4) Participants can utilize screen share to display the document and provide comments/suggested re-writes.

5) Facilitators can then share suggested re-writes with the group.

Suggested Re-writes. Please note that the groups may suggest more specific examples, additional comments provided by direct observation, comments on suggestions for growth/improvement. The rewrites below are meant to spark conversation and provide a potential example.

**Checkist and Suggested Re-writes**

**Jessica: “Team player.  Excellent knowledge base.  Knew her patients well.  Always willing to do extra work.  Pleasure to have on the rotation. Should continue reading to learn more.”**

**Checklist:** Does not provide specific comments. Comments not clearly based on direct observation of the learner. Lacks examples when describing strengths. Does cover several competencies but not thoroughly. Describes an area for growth but only in terms of “continue reading to learn more.”

**Rewrite:** Jessica demonstrated an exceptional knowledge base. As one example, she reminded the team that an evaluation for colon cancer was necessary prior to taking a patient for gynecologic surgery. She showed attention to detail and knowledge of her patients in answering all questions about her patient’s histories, physicals and lab values. She offered help to each team member still present before leaving for the day. As I discussed with her toward the end of the rotation, the next step for Jessica is to be more concise in her oral presentations.

**Stuart: “Very professional student.  Impressive knowledge base and strong differential diagnoses.  Efficient.  Comes in early.  Great oral presentations.”**

**Checklist:** Does not provide specific comments.  Does cover several competencies but doesn’t provide specific examples to support areas of strength.  Does not describe any areas for growth.

**Rewrite:** Stuart demonstrated professionalism and humanism by recognizing that a patient had transportation difficulties and suggested we try to coordinate follow up with her other subspecialty appointments. He is also developing excellent clinical judgment. For example, for a 3-year-old patient in the ER, he quickly recognized a worsening in respiratory status while taking the history and notified the nurse immediately. For the majority of the patients he saw, he was able to logically present the pertinent positives and negatives for most likely and can’t miss possibilities and propose the correct diagnostic workup.

**Amy: “Amy has done an excellent job on this rotation.  She is responsible, always on time, reads about the subjects assigned to her.  She was prepared and read for each returning patient and knew guidelines very well.  Helpful in clinic.”**

**Checklist:** This one is a little more thorough than the others in that it provides examples of the student reading on patients and knowing guidelines. Additional examples to support “excellent job” would strengthen the narrative, specifically comments that are based on direct observation.  Several competencies are addressed.

**Rewrite:** On the outpatient segment of the rotation, Amy was always on time. By the end of the rotation, she was reviewing patient charts before clinic started, participating in the team huddle and asking good questions of the nursing staff and social workers. In her own downtime, she took initiative to call patients in and take vitals.  She used the apps for the CDC STI and contraceptive guidelines and asked appropriate questions about their applicability to patients with special circumstances. She asked for help with her sexual history taking skills and after direct observation quickly responded to feedback on how to improve.

**Lindia: “Lindia was a rock star on this rotation. She really has what it takes to be a great surgeon.  Her dexterity was superb for a medical student. We will really miss her on the team and hope she applies to this program.”**

**Checklist:** Comments are superlative but lack specificity on why she is a “rock star” and “has what it takes to be a great surgeon.” Specific examples would be helpful here, especially if the supervisor directly observed her dexterity and when. The comment on hoping she applies to the program is positive but not supported by any direct observations or additional information.

**Rewrite**: Lindia is always prepared and read in depth for each case – for example, she was able to describe each major step of a lap chole and identify all of the relevant anatomy during the case with almost no prompting.  She performed IV insertion correctly in the OR. She also demonstrated adept handling of camera and efficient skin suturing.  Was able to answer all questions about her patients’ clinical course.  Her resident allowed her to do sign-outs on her patients during the second half of the rotation and said she included all of the important background information and correct assessments and recommendations. Her next step there is to be more concise. Very respectful and always willing to help.
