# Supplementary material for: Preparing Effective Narrative Evaluations for the Medical School Performance Evaluation (MSPE)
Source: MedEdPORTAL. 2022 Oct 4;18:11277. doi: 10.15766/mep_2374-8265.11277 (PMC9529862; doi:10.15766/mep_2374-8265.11277)
Supplement: Supplementary file 1 — Narrative Evaluations for the MSPE.pptxFacilitator Guide.docxActivity 1.docxActivity 2.docxActivity 2 Facilitator Guide.docxActivity 3.docxActivity 3 Facilitator Guide.docxEvaluation Form.docx [file mep_2374-8265.11277-s001.zip › G. Activity 3 Facilitator Guide.docx]

**Appendix G**

**Breakout Activity #3: Facilitator’s Guide**

**Writing Clerkship Summary Paragraphs**

**In-Person:**

1) Prepare copies of Appendix F for participants

2) Bring green highlighters and pens for the groups

3) Have participants work in groups to complete Appendix F

4) A facilitator for each group is ideal or if only one or two facilitators, they can walk around to the various groups

5) Facilitator can share Appendix G information with participants in their individual groups or in large group format

**Virtual:**

1) Paste Appendix F in the chat

2) Utilize breakout rooms to create small groups

3) Have one of the participants (or a facilitator) display Appendix F on the screen and follow the instructions for highlighting, striking, underlining competencies, and writing as described in the instructions for Appendix F

5) Facilitators can then share suggested re-writes with the individual groups or bring everyone back together in large group

**Example 1:**

- “~~John is a good medical student and will make a good pediatrician.~~ He is compassionate and has a positive attitude.” Professionalism
- ~~“John is a pleasure to have in clinic and on the team.”~~
- “John actively reads about his patient’s diagnoses and other potential differential diagnoses. He has a **strong biomedical science knowledge** background that he is able to apply to clinical care.”
- “John demonstrates a work ethic well above other students. He truly cares about the wellbeing of his patients and goes above and beyond to assure all labs and consults are followed up on. For one patient, his realization that the Infectious Disease team wanted to place a PICC line in a patient resulted in him letting the team know that the patient would need to be NPO after midnight, saving the patient an extra day in the hospital.” **Professionalism and Patient Care**
- “John actively engaged in rounds, taking notes on ALL patients, not just his own. He was quick to pick-up new patients when his patients were discharged. His presentations were thorough yet effectively communicated pertinent positives and negatives; he took the time to formulate a broad differential and plan on each and every patient.” **Communication**
- “John demonstrated tremendous care, compassion, and engagement. One afternoon when I went to see a new admission, I saw him in the room with one of our school-aged patients who had been diagnosed with transverse myelitis. He was playing Connect-Four with her talking with her some about her illness and her fears and concerns about being in the hospital. He would make an excellent pediatrician.” **Patient Care and Communication**
- “John took the time one day to meet with the nutritionist, to better learn about infant formulas and calories in order to better care for one of his failure to thrive patients. The nutritionist found me to let me know how respectful he was of her knowledge and appreciative of her time to help him learn.” **Patient Care, Interprofessional Communication, Practice-Based Learning and Improvement**
- ~~“Good job! Enjoyed having you on the team.”~~

**Potential Summary Paragraphs for Part 1:**

John received “above expectations” marks in the areas of medical knowledge, patient care, and practice-based learning and improvement. He was “excellent” in professionalism and communication. Specific comments from the attendings and residents who worked with him include:

Add green quotes. Consider adding others if competency not otherwise represented.

OR a different initial summary statement:

John was noted to be actively engaged on inpatient rounds, possess a strong biomedical science knowledge background that he is able to apply clinically, and demonstrate a positive attitude. Specific comments from his rotation include:

Add green quotes. Consider adding others if competency not otherwise represented.

**Example 2:**

- “Chris is a good student. He saw 3 -4 infants per day on his newborn nursery rotation and was effective in his ‘discharge talk’ with families.” **Communication**

“Chris often arrived late to clinic and missed the morning clinic lecture three times. When I asked him why he was late, he apologized and said he overslept and on another occasion, he said didn’t feel well but was better now and able to come in.” **Professionalism**

- “Student met expectations in his one week spent in the resident clinic. His medical knowledge base is exceptional and he quickly learned the importance of anticipatory guidance using Bright Futures materials.” **Medical Knowledge**
- “Chris was extremely engaged during his week in a private pediatric office. He went in to see patients on his own, and then presented the information to me in a thorough and logical manner. He demonstrated a solid knowledge of common pediatric problems such as otitis media, asthma, and skin and musculoskeletal complaints.” **Professionalism, Communication**
- “Chris did not fully engage with the inpatient pediatric team. There were multiple occasions where we (the residents) could not locate him and he took over 30 minutes sometimes to respond to text messages. We had to ask him to pick up patients each morning, even when his patients had been discharged the evening before. I met with him after the first week to give him feedback, and he did begin to pick up additional patients the second week and seemed to know the patients better. However, I am still concerned about his overall lack of motivation.” **Professionalism**

**Chris remediated two weeks of his pediatric rotation. Comments from the remediation include:**

- “Chris arrived on time each day, pre-rounding on all of his patients and communicating with the residents about any patient changes overnight. He seemed to know his patients well and carried a comparable load to other students on the team.” **Professionalism and Communication**
- “Chris gave an excellent presentation on “infants with hypotonia and potential diagnoses” to the team. He actively engaged on rounds and in learning sessions”

**Potential summary paragraph for Activity 2:** (yellow highlights are added by the Clerkship Director)

- Chris has a solid base of medical knowledge and received some positive evaluations specifically in the outpatient clinic and nursery but two evaluators in the inpatient setting noted that he did not fully engage during his Pediatric rotation. The outpatient clinic physician stated, “Student met expectations in his one week spent in the resident clinic. His medical knowledge base is exceptional and he quickly learned the importance of anticipatory guidance using Bright Futures materials.” Another community attending stated, “Chris was extremely engaged during his week in a private pediatric office. He went in to see patients on his own, and then presented the information to me in a thorough and logical manner. He demonstrated a solid knowledge of common pediatric problems such as otitis media, asthma, and skin and musculoskeletal complaints.” In the nursery, “Chris is a good student. He saw 3 -4 infants per day on his newborn nursery rotation and was effective in his ‘discharge talk’ with families.” The issues raised in the inpatient setting included tardiness to morning lectures, not responding to text messages in a timely manner, and failure to see new patients. A specific comment stated.” I met with him after the first week to give him feedback, and he did begin to pick up additional patients the second week and seemed to know the patients better. However, I am still concerned about his overall lack of motivation.” As the clerkship director, I met with Chris at the end of the rotation and he disclosed some personal issues that impacted his performance and we decided he would repeat two weeks of inpatient pediatrics. There were no concerns raised during that time and in fact, he received the following comments. “Chris arrived on time each day, pre-rounding on all of his patients and communicating with the residents about any patient changes overnight. He seemed to know his patients well and carried a comparable load to other students on the team.” Chris gave an excellent presentation on “infants with hypotonia and potential diagnoses” to the team. He actively engaged on rounds and in learning sessions.
