# Supplementary material for: Preparing Effective Narrative Evaluations for the Medical School Performance Evaluation (MSPE)
Source: MedEdPORTAL. 2022 Oct 4;18:11277. doi: 10.15766/mep_2374-8265.11277 (PMC9529862; doi:10.15766/mep_2374-8265.11277)
Supplement: Supplementary file 1 — Narrative Evaluations for the MSPE.pptxFacilitator Guide.docxActivity 1.docxActivity 2.docxActivity 2 Facilitator Guide.docxActivity 3.docxActivity 3 Facilitator Guide.docxEvaluation Form.docx [file mep_2374-8265.11277-s001.zip › H. Evaluation Form.docx]

**Appendix H**

**Evaluation Form (adapted with permission from COMSEP)**

| **Satisfaction, Format and Value** | Strongly Agree  5 | Agree  4 | 3 | Disagree 2 | Strongly  Disagree 1 |
| --- | --- | --- | --- | --- | --- |
| Overall, the workshop was effective. |  |  |  |  |  |
| Overall, the speakers were effective. |  |  |  |  |  |
| The format of this activity was appropriate for its content. |  |  |  |  |  |
| This activity was a worthwhile investment in my professional development. |  |  |  |  |  |

| **Objectives and Learning** | Strongly Agree  5 | Agree  4 | 3 | Disagree 2 | Strongly  Disagree 1 |
| --- | --- | --- | --- | --- | --- |
| I learned new knowledge and skills from this activity. |  |  |  |  |  |
| I will apply the knowledge and skills. |  |  |  |  |  |
| This activity is relevant to my professional role. |  |  |  |  |  |

| **Overall Experience** | Excellent | Very Good | Good | Fair | Poor | Very poor |
| --- | --- | --- | --- | --- | --- | --- |
| Session as a whole was: |  |  |  |  |  |  |
| Content of session was: |  |  |  |  |  |  |
| Presentation of materials was: |  |  |  |  |  |  |
| Use of session time was: |  |  |  |  |  |  |
| Handouts (if applicable) were: |  |  |  |  |  |  |

Did the session meet your expectations?

**Yes**  **Somewhat**  **No**

Would you recommend this session to another faculty member?

**Yes**  **No**

What did you like best about this workshop?

What did you like least about this workshop? How can it be improved?
